# Estimation of Effect Heterogeneity in Rare Events Meta-Analysis

**DOI:** 10.1007/s11336-021-09835-5

**Published:** 2022-02-08

**Authors:** Heinz Holling, Katrin Jansen, Walailuck Böhning, Dankmar Böhning, Susan Martin, Patarawan Sangnawakij

**Affiliations:** 1 Institute of Psychology, University of Münster, Fliednerstr. 21, 48149 Münster, Germany; 2 University of Southampton, Southampton, UK; 3 Thammasat University, Bangkok, Thailand

**Keywords:** heterogeneity variance, count data analysis, nonparametric mixture models, meta-analysis, generalised linear mixed models, rare events

## Abstract

The paper outlines several approaches for dealing with meta-analyses of count outcome data. These counts are the accumulation of occurred events, and these events might be rare, so a special feature of the meta-analysis is dealing with low counts including zero-count studies. Emphasis is put on approaches which are state of the art for count data modelling including mixed log-linear (Poisson) and mixed logistic (binomial) regression as well as nonparametric mixture models for count data of Poisson and binomial type. A simulation study investigates the performance and capability of discrete mixture models in estimating effect heterogeneity. The approaches are exemplified on a meta-analytic case study investigating the acceptance of bibliotherapy.

**Supplementary Information:**

The online version contains supplementary material available at 10.1007/s11336-021-09835-5.

Meta-analyses are used to analyse and integrate the results of several studies investigating the same research question, providing a less costly and more powerful alternative to a large new single study. For a general introduction into meta-analysis, refer to Borenstein et al. (2009) or Schulze et al. (2003), for example, and specifically for psychology see Bonett and Price (2014, 2015). The following meta-analytic setting, tailored for event data, was considered in Böhning et al. (2015) and shall be the focus of this paper. In *k* independent studies, counts of events are observed in an intervention and control group. This setting can be described by a random count variable $Y_{ij}$. The index *i* indicates the study *i* for $i=1,2,\ldots ,k$, where *k* denotes the number of available studies. Also, $j=1$ denotes the intervention group and $j=0$ the control group. $Y_{ij}$ represents the number of events in study *i* and group *j*, whereas $n_{ij}$ denotes the sample size in study *i* and group *j*. The latter is considered as non-random and is also called the number at risk.

A conventional two-stage meta-analysis proceeds as follows. In the first stage, an estimate of an effect size such as the relative risk $\widehat{RR_i} =\frac{Y_{i1}/n_{i1}}{Y_{i0}/n_{i0}}$ or odds ratio ${\widehat{OR}}_i = \frac{Y_{i1}/(n_{i1}-Y_{i1})}{Y_{i0}/(n_{i0}-Y_{i0})}$ is computed for each study. Then, in a second stage, these estimates are further analysed, for example, by providing a summary measure $\sum _i w_i {\hat{\theta }}_i /\sum w_i$ where ${\hat{\theta }}_i$ is often taken on the log-scale for the risk or odds ratio. Here, the $w_i$ are weights and often chosen proportional to the inverse variance of ${\hat{\theta }}_i$, where the latter is the estimated risk ratio or odds ratio. In contrast, we focus here on a one-stage approach which directly models the observed counts. This approach has several benefits, as it allows for the inclusion of zero-count studies which either need to be excluded in the two-stage approach or zeros need to be replaced by a smoothing constant as their effect sizes and associated variances are not defined. Also, it involves working with more appropriate distributions such as the Poisson or binomial distribution and avoids normal approximations involved in the two-stage approach.

The meta-analytic one-stage approach is closely connected to multilevel analysis and modelling as studies introduce a natural hierarchical level in the data. As pointed out in Hox et al. (2017), meta-analysis can be viewed as an example of multilevel analysis which is prominently used in the social sciences. In particular, it is a two-level approach where the first level is the sample of studies from the population of all possible studies and the second level is the sample of the study participants. Approaches differ depending on what study-specific information is available. In the conventional approach, it is assumed that only a summary measure such as an odds ratio, relative risk or correlation coefficient is available accompanied by some uncertainty measure. If patient-level data are available, Skrondal and Rabe-Hesketh (2004, 299–307) suggest to model these in a multilevel approach. Riley et al. (2010) point out the value and beneficiaries of individual participant data meta-analysis. In practice, however, the problem remains to obtain access to individual participant data of all retrieved relevant studies. To address the issue that only summary information is available for some studies, whereas for others individual participant data are available, Riley et al. (2008) suggest approaches to combine these different types of information. In our setting, we are in between the two extreme scenarios of either having only summary measures for all individual participant data as we have more than a summary measure—there are four cell frequencies which allow various choices of the effect measure—but we are also clearly not in the situation of an individual patient data meta-analysis.

In summary, we outline the major novel aspects of the paper in the following:

- Rare events meta-analysis experiences serious drawbacks if conducted following a conventional pathway. Effect measures such as risk or odds ratio might be undefined, as would be the associated approximate variance estimates, unless continuity corrections are invoked with unclear bias potential. In addition, the within-study normality assumption for the effect measure might be seriously in doubt. Here, it is suggested to use count model approaches such as generalised linear and generalised mixed models, as they have been developed and well-investigated for counts and found to perform considerably well.
- More importantly, finite mixture models are suggested to replace the parametric (normal) random effects distribution. We see this as an important step towards creating a new generation of two-level nonparametric meta-analytic approaches. Here we propose to allow mixing on baseline and, potentially, on the effect parameter itself. In addition, we demonstrate in simulation work that these methods can be used successfully in identifying the underlying risk structure.

The paper is organised as follows. Section 1 contains a case study which introduces the setting and its issues. Section 2 presents the conventional log-linear and logistic modelling adapted for meta-analytic applications, followed by Sect. 3 which discusses how baseline heterogeneity can be modelled. Section 4 introduces mixed log-linear and logistic regression modelling to cope with effect heterogeneity. Finally, in Sect. 5, the parametric normal random effects distribution is replaced by a nonparametric random effect which is estimated by means of a discrete mixture model. All models and approaches are illustrated using the case study. Section 6 adds a simulation study which mirrors the case study data in its design and illustrates the capability of mixture models in identifying heterogeneity. The paper ends in Sect. 7 with a short discussion.

## Case Study on Bibliotherapy vs. Control for Acceptability of the Intervention

We use meta-analytic data on the acceptability of bibliotherapy compared with control groups in the treatment of children and adolescents with depression and/or anxiety from eight studies to illustrate the application of the risk ratio and odds ratio. These data were provided by Yuan et al. (2018). Here, bibliotherapy or so-called book therapy is a treatment approach to mental health. This method is often used to support several conditions via therapy, because of its ease of use, low cost and greater privacy. The control condition comprises wait-list control, non-treatment control, treatment as usual, and psychological placebo. However, the question arises whether bibliotherapy is favourable for the acceptability of the treatment plan for a diagnosis of depression and/or anxiety. According to Yuan et al. (2018), acceptability is defined as all-cause discontinuation, i.e. the proportion of patients who discontinued treatment for any reason. According to this definition, high proportions occur when acceptability is low.

**Fig. 1:**
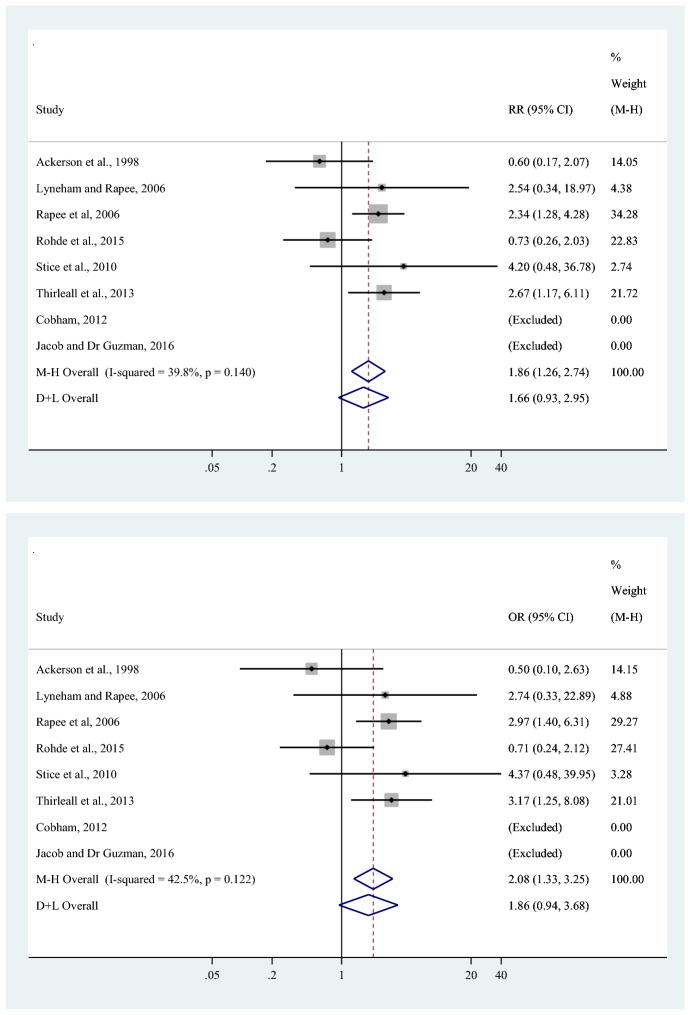
Forest plots of bibliotherapy and control conditions for acceptability, risk ratio (upper panel) and odds ratio (lower panel) are reported.

Meta-analytic data of bibliotherapy and control conditions for acceptability used in this example are given in Table 1. We can see that in many studies, only a few participants discontinued treatment, compared to the total number of participants. Moreover, the data contain studies with zero events in both arms (double-zero studies). Thus, when we use the traditional inverse variance-weighted average method in meta-analysis for combining the risk ratios and odds ratios, the two double-zero studies will be excluded before the analysis, as is shown by the forest plot in Fig. 1. Under homogeneity of the effect size (the associated tests of homogeneity have a *p*-value of 0.14 for the risk ratio and 0.12 for the odds ratio), the estimated overall risk ratio and overall odds ratio are given by 1.86 and 2.08, respectively. These are obtained with the Mantel–Haenszel estimator which allows zero-containing studies, and of which details are given in the following section. It can therefore be hypothesised that all-cause discontinuation is observed more often in bibliotherapy than in control conditions, indicating lower acceptability of bibliotherapy in children and adolescents with depression and/or anxiety. This question will now be further investigated in the following sections.

## Log-Linear and Logistic Regression Models as an Extension of the Relative Risk and Odds Ratio

In the following, we look at the relative risk and odds ratio, and how they generalise to log-linear and logistic regression. Details can be found in Jewell (2004), for example.

Conditional upon study *i*, the relative risk is modelled using the log-linear model

1 $$\begin{aligned} \log E(Y_{ij}) =\log n_{ij} + \alpha _i + \beta _i \times j, \end{aligned}$$

where $j=0$ represents the control group and $j=1$ the intervention group. Hence, $\exp (\beta _i) = \frac{E(Y_{i1}/n_{i1})}{E(Y_{i0}/n_{i0})}$ represents the risk ratio in study *i*, as can be seen by substituting in $j=0$ and $j=1$ into (1) and taking differences. The log-linear model (1) is often combined with a conditional Poisson assumption, although alternatives and extensions such as the negative binomial model are possible (Hilbe, 2012). If we replace the expected values by their observed counterparts, we obtain the *empirical* risk ratio $\frac{Y_{i1}/n_{i1}}{Y_{i0}/n_{i0}}$ for study *i*, assuming that $Y_{i0}$ is positive.

A second measure is the odds ratio. Here, conditional upon study *i*, the odds ratio is modelled using the logistic model

2 $$\begin{aligned} \log \frac{E(Y_{ij})}{n_{ij}-E(Y_{ij}) } = \alpha _i + \beta _i \times j, \end{aligned}$$

where $j=0$ represents the control group and $j=1$ the intervention group. Hence, $\exp (\beta _i) =\frac{E(Y_{i1})/(n_{i1}-E(Y_{i1}))}{E(Y_{i0})/(n_{i0}-E(Y_{i0}))}$ represents the odds ratio in study *i*, as can be seen by substituting in $j=0$ and $j=1$ into (2) and taking differences. The logistic model (2) is often combined with a conditional binomial assumption. Again, alternatives are possible including the beta-binomial model as suggested in Kuss (2015).

Again, if we replace the expected values by their observed counterparts, we find the *empirical* odds ratio $\frac{Y_{i1}/(n_{i1}- Y_{i1})}{ Y_{i0}/(n_{i0}- Y_{i0})}$ for study *i*, assuming that $0< Y_{ij} < n_{ij}$ holds for all *i* and $j=0,1$.

## Baseline Heterogeneity as Fixed or Random Effects and Mantel–Haenszel Estimation

Here, we consider a specific case of models (1) and (2), namely the case of *effect homogeneity*. In other words, we consider that $\beta _i=\beta$ for all *i*. We would still like to keep a specific intercept value $\alpha _i$, which is called the *baseline heterogeneity* as it refers to the risk or odds in the control group, which represents the baseline population. Two approaches are possible. In the first approach, a parameter estimate ${\hat{\alpha }}_i$ is fitted for each baseline parameter $\alpha _i$ of study *i*. This is sometimes called a *contrast-based* approach. In the second approach $\alpha _i$ is assumed to arise from a distribution, often a normal distribution $\alpha _i \sim N(\alpha ,\sigma ^2_\alpha )$. This is called an *arm-based* approach.

We emphasise here the difference between a contrast-based approach and an arm-based approach–which refers largely to the way the intercept is modelled. In an approach, where the study factor is ignored, there is high potential for confounding. We illustrate this issue in the discussion with a synthetic example. As there is agreement that the study factor should be adjusted for, the question arises how this can be best accomplished. One opinion is that one should avoid situations where interventions are compared across studies, which can be achieved by entering study as a main effect. The concern here is not whether the main effect parameters of the study factor (baseline parameters) are estimated validly (a Neyman–Scott problem may occur referring to the issue of consistently estimating infinitely many nuisance parameters), the question is whether we can achieve an unconfounded estimate of the effect of interest. Another opinion is that an unconfounded estimate of the effect can be achieved by treating the main effect of study as a random effect (which is considered critically by proponents of the first opinion as interventions are at risk of being compared across studies). We continue here with both approaches and will see that they both lead to identical effect estimates.

**Table 1:** Meta-analytic data on bibliotherapy and control conditions for acceptability.

Study, year	Bibliotherapy		Control	
	Events	Total	Events	Total
Ackerson et al. (1998)	3	15	5	15
Cobham (2012)	0	20	0	12
Jacob and De Guzman (2016)	0	15	0	15
Lyneham and Rapee (2006)	9	78	1	22
Rapee et al. (2006)	29	90	12	87
Rohde et al. (2015)	6	128	8	124
Stice et al. (2010)	4	80	1	84
Thirlwall et al. (2013)	29	125	6	69

**Table 2:** Effect estimates under fixed and random baseline heterogeneity as well as Mantel–Haenszel estimation (MHE).

Model	Log-linear model			Logistic model		
	AIC	BIC	${\widehat{RR}}$, $95 \%$ CI	AIC	BIC	${\widehat{OR}}$, $95 \%$ CI
Fixed	69.22	76.18	1.84 [1.22, 2.77]	68.90	75.85	2.09 [1.33, 3.27]
Random	84.43	86.75	1.84 [1.23, 2.76]	84.77	87.08	2.08 [1.33, 3.23]
MHE			1.86 [1.26, 2.74]			2.08 [1.33, 3.25]

We also mention here the Mantel–Haenszel approach (Mantel & Haenszel, 1959; Jewell, 2004; Greenland & Robbins, 1985) which provides effect estimates of $\theta$ without any need to model the baseline heterogeneity. For the risk ratio, the Mantel–Haenszel estimator is defined as

$$\begin{aligned} {\widehat{RR}}_{\text{ MH }}= \frac{\sum _i Y_{i1}n_{i0}/n_i}{\sum _i Y_{i0}n_{i1}/n_i} \end{aligned}$$

and for the odds ratio

$$\begin{aligned} {\widehat{OR}}_{\text{ MH }}= \frac{\sum _i Y_{i1}(n_{i0}-Y_{i0})/n_i}{\sum _i Y_{i0}(n_{i1}-Y_{i1})/n_i}, \end{aligned}$$

where $n_i=n_{i0}+n_{i1}$. Note that these Mantel–Haenszel estimators are weighted estimators of the study-specific risk ratios ${\widehat{RR}}_i$ and odds ratios ${\widehat{OR}}_i$, respectively, as they can be written in the form ${\widehat{RR}}_{\text{ MH }}=$ $\sum _i w_i {\widehat{RR}}_i / \sum _i w_i$ and ${\widehat{OR}}_{\text{ MH }}$ $=\sum _i w_i {\widehat{OR}}_i$ $/ \sum _i w_i$ using the Mantel–Haenszel weights $w_i = Y_{i0}n_{i1}/n_i$ for the risk ratio and $w_i = Y_{i0}(n_{i1}-Y_{i1})/n_i$ for the odds ratio, respectively, assuming that these weights exist, i.e. $0< Y_{ij} < n_{ij}$ for all *i* and $j=0,1$ (Mantel & Haenszel, 1959).

Table 2 shows the performance and model evaluations for the case study data. Note that we have included the values of the *Akaike information criterion* (AIC) and *Bayesian information criterion* (BIC) for each model considered here. The overall message here is that all approaches perform similarly. Estimating log-linear and logistic models with fixed and random intercepts, i.e. without and with baseline heterogeneity, we find that the risk of all-cause discontinuation is significantly increased by a factor of 1.84 for bibliotherapy and that the odds for all-cause discontinuation are significantly increased by a factor of 2.09 in bibliotherapy. In addition, confidence intervals are fairly similar.

## Effect Heterogeneity as Mixed Effects Model

To model effect heterogeneity of the effect measure $\beta _i$ in the mixed model approach, it is assumed that $\beta _i \sim N(\beta ,\tau ^2)$. If there is no heterogeneity, i.e. $\tau ^2=0$, the overall effect can be summarised as a single value. For this setting and the risk ratio with baseline heterogeneity modelled as a fixed effect, the likelihood takes the form

3 $$\begin{aligned} \prod _i \int _{\beta _i} [ Po(y_{i0}|\exp (\log n_{i0} + \alpha _i)) \times Po(y_{i1}|\exp (\log n_{i1} + \alpha _i) +\beta _i))] \phi (\beta _i|\beta ,\tau ^2) d\beta _i \end{aligned}$$

where $Po(y|\mu ) = \exp (-\mu )\mu ^y/y!$ is the Poisson density. $\phi (\beta _i|\beta ,\tau ^2)$ is the normal density with mean $\beta$ and variance $\tau ^2$. Similarly, we obtain for the odds ratio

4 $$\begin{aligned} \prod _i \int _{\beta _i} [ Bi(y_{i0}|n_{i0}, \text{ expit }(\alpha _i)) \times Bi(y_{i1}|n_{i1}, \text{ expit }(\alpha _i +\beta _i))] \phi (\beta _i|\beta ,\tau ^2) d\beta _i \end{aligned}$$

where $\text{ expit }(\eta ) =\exp (\eta )/(1+\exp (\eta ))$ and $Bi(y|n,\mu ) ={n \atopwithdelims ()y} \mu ^y(1-\mu )^{n-y}$ is the binomial density. Note that in these likelihoods, the baseline parameters $\alpha _i$ are treated as unknown but fixed quantities. If we model them as random quantities as well, we obtain the following nested likelihood for the odds ratio in the binomial model

5 $$\begin{aligned} \prod _i \int _{\alpha _i} \big \{\int _{\beta _i} [ Bi(y_{i0}|n_{i0}, \text{ expit }(\alpha _i))&\times Bi(y_{i1}|n_{i1}, \text{ expit }(\alpha _i +\beta _i))]\nonumber \\&\times \phi (\beta _i|\beta ,\tau ^2) d\beta _i \big \} \phi (\alpha _i|\alpha ,\sigma ^2_{\alpha }) d\alpha _i. \end{aligned}$$

Likewise, the nested likelihood for the risk ratio in the log-linear model can be obtained as

$$\begin{aligned} \prod _i \int _{\alpha _i} \big \{\int _{\beta _i} [ Po(y_{i0}|\exp (\log n_{i0} + \alpha _i))&\times Po(y_{i1}|\exp (\log n_{i1} + \alpha _i) +\beta _i))]\\&\times \phi (\beta _i|\beta ,\tau ^2) d\beta _i \big \} \phi (\alpha _i|\alpha ,\sigma ^2_{\alpha }) d\alpha _i. \end{aligned}$$

A key difference between the fixed and random baseline heterogeneity models is the number of parameters. Whereas the former has $k+2$ parameters, the latter has only 4. Note that in the fixed baseline heterogeneity model the number of parameters grows with the number of studies, whereas the number remains unchanged in the case of the random baseline heterogeneity model.

In Table 3, we see the results of the model fitting for both models. Whereas the effect estimates differ only slightly across the two models, heterogeneity variance estimates show considerable differences. In the fixed baseline heterogeneity model, the variance of the effect heterogeneity estimate is zero.

For comparison, we included in Table 3 the estimates of the standard inverse variance model along with the heterogeneity variance estimate of DerSimonian and Laird (1986), which is based on the two-stage analysis. First, the $\chi ^2$-statistic

$$\begin{aligned} Q=\sum _{i=1}^k w_i({\hat{\theta }}_i -{\bar{\theta }})^2 \end{aligned}$$

is computed, where $w_i=1/\sigma _i^2$ and $\sigma _i^2$ is the estimated variance of the effect measure of interest, here the log-relative risk or log-odds ratio. Furthermore, ${\bar{\theta }}$ is the log Mantel–Haenszel summary estimate of the respective measure of interest. Then, the DerSimonian–Laird estimator for $\tau ^2$ is given as

$$\begin{aligned} {\hat{\tau }}^2 = \frac{Q-(k-1)}{\sum _i w_i-(\sum _i w_i^2)/\sum _i w_i} \end{aligned}$$

with the understanding that the estimator is truncated to 0 if it becomes negative. The DerSimonian–Laird estimate in Table 3 is also positive, but larger than the heterogeneity variance estimate under random baseline heterogeneity.

**Table 3:** Effect estimates under fixed and random baseline heterogeneity with effect heterogeneity modelled by a normal distribution $\beta _i \sim N(\beta ,\tau ^2)$ as well as the Inverse Variance model (IV); DL stands for the DerSimonian–Laird estimate of the heterogeneity variance.

Model	Log-linear model				Logistic model			
	AIC	BIC	${\widehat{RR}}$, $95 \%$ CI	$\hat{\tau ^2}$, $95 \%$ CI	AIC	BIC	${\widehat{OR}}$, $95 \%$ CI	${\hat{\tau }}^2$, $95 \%$ CI
Fixed	71.22	78.95	1.84 [1.22, 2.77]	0.00 [0, 0.43]	70.90	78.63	2.09 [1.33, 3.27]	0.00 [0, 0.59]
Random	86.29	89.38	1.73 [1.00, 3.00]	0.07 [0, 1.10]	86.06	89.15	1.83 [0.97, 3.45]	0.17 [0, 1.33]
IV DL			1.66 [0.93, 2.95]	0.19 [0, 3.16]			1.86 [0.94, 3.86]	0.29 [0, 4.19]

## Nonparametric Heterogeneity Modelling for the Log-Linear and Logistic Model

### The Log-Linear Model with Heterogeneity

In the following, we lay out how a nonparametric random effects approach can be developed using nonparametric mixture models. Key theoretical results can be found in Lindsay (1995), whereas computational validity of maximum likelihood estimation is provided in Böhning (1982, 1989). An introduction into mixture models is given in Böhning (2000) and applications in psychology are provided in Doebler and Holling (2015), Holling et al. (2012) and Malzahn et al. (2000).

The modelling approach that we are presenting for heterogeneity estimation is detailed as follows. Recall that model (1) is given by:

$$\begin{aligned} \log E(Y_{ij}) = \alpha _i +\beta _i \times j+ \log n_{ij}. \end{aligned}$$

Also recall that the log-risk ratio of this model in the *i*th study is given by $\beta _i$ and corresponds to $\exp (\beta _i)= RR_i$. In addition, heterogeneity can now be separated into *baseline* heterogeneity—the variability in the intercept $\alpha _i$—and heterogeneity in the effect measure—the variability in the slope $\beta _i$. The presence of effect homogeneity is characterised by $\beta _i=\beta$ for all studies $i=1, 2,\ldots , k$. In the previous section, we modelled heterogeneity using a generalised linear mixed model approach which takes $\alpha _i \sim N(\alpha ,\sigma ^2_\alpha )$ and $\beta _i \sim N(\beta ,\sigma ^2_\beta )$. Now, instead of assuming a normal (or other parametric) distribution, we leave the distribution of $(\alpha _i,\beta _i)$ unspecified. From the foundations of nonparametric maximum likelihood estimation, the maximum likelihood estimator maximising the mixture log-likelihood with mixing distribution *Q*

6 $$\begin{aligned} \ell (Q) =\sum _{i,j} \log \left[ \int p(y_{ij};\exp (\alpha _i +\beta _i \times j+ \log n_{ij})) Q(d\alpha _i,d\beta _i)\right] \end{aligned}$$

is always discrete (Lindsay, 1983, 1995). Here, $p(y;\lambda ) = \exp (-\lambda )\lambda ^y/y!$ is the Poisson discrete mass function for $y=0, 1,\ldots$ and $\lambda > 0$. Hence, there is no limitation of generality if we replace (3) by

7 $$\begin{aligned} \ell (Q) =\sum _{i,j} \log \left[ \sum _{s=1}^S p(y_{ij};\exp (\alpha _s +\beta _s \times j+ \log n_{ij})) q_s\right] . \end{aligned}$$

The log-likelihood (4) is evidently a discrete mixture log-likelihood with weights $q_1, q_2,\ldots , q_S$ being positive and summing up to 1. Unfortunately, it is not known which value for *S* should be chosen. This is known as *the number of components problem*. A typical solution is to start with $S=1$ and then sequentially increase the number of components by one until no further increase in the log-likelihood is detected. Specifically, for a given value of *S*, the log-likelihood (7) is maximised using the EM algorithm (Dempster et al., 1977; McLachlan & Krishnan, 2007). More details on computational and algorithmic approaches for mixture likelihood problems can be found in Böhning (2000).

We will denote the maximum likelihood estimate of the parameters $\alpha _s, \beta _s$ and $q_s$ for $s=1, 2,\ldots , S$ as

$$\begin{aligned} {\hat{Q}} = \begin{pmatrix}{\hat{\alpha }}_1 &{}\cdots &{}{\hat{\alpha }}_S\\ {\hat{\beta }}_1 &{}\cdots &{}{\hat{\beta }}_S\\ {\hat{q}}_1 &{}\cdots &{}{\hat{q}}_S \end{pmatrix}. \end{aligned}$$

Note that ${\hat{Q}}$ is a mixing distribution jointly on the intercept $\alpha$ and the slope (log-risk ratio) $\beta$, in other words it is a discrete distribution giving weights ${\hat{q}}_s$ to intercept and slope combinations $({\hat{\alpha }}_s,{\hat{\beta }}_s)$. Having the maximum likelihood estimate available, we are then able to give a nonparametric estimate of the heterogeneity variance of the log-risk ratio as

$$\begin{aligned} {\hat{\tau }}^2 = \sum _{s=1}^S ({\hat{\beta }}_s - {\bar{\beta }})^2{\hat{q}}_s, \end{aligned}$$

where ${\bar{\beta }} = \sum _{s=1}^S {\hat{q}}_s {\hat{\beta }}_s$. This variance is of particular interest in meta-analysis as its size indicates the amount of heterogeneity in effect size across studies. Of course, other variances such as the baseline heterogeneity variance in the $\alpha _s$ can also be considered.

### The Logistic Model with Heterogeneity

The basic logistic model takes the form

8 $$\begin{aligned} \log \frac{E(Y_{ij})}{n_{ij}-E(Y_{ij}) } = \alpha _i + \beta _i \times j, \end{aligned}$$

where $j=0$ represents the control group and $j=1$ the intervention group and $\beta _i$ is the log-odds ratio in the *i*th study. The discrete mixture likelihood now becomes

9 $$\begin{aligned} \ell (Q) =\sum _{i,j} \log \left[ \sum _{s=1}^S p(y_{ij};n_{ij},\text{ expit }(\alpha _s +\beta _s \times j)) q_s\right] , \end{aligned}$$

where $$p(y;n,\mu )= \left( {\begin{array}{c}n\\ y\end{array}}\right) \mu ^y (1-\mu )^{n-y}$$ and $\text{ expit }(x) = \exp (x) /[1+\exp (x)]$ for any real *x*.

Table 4 presents the results of the mixture model analysis for the log-linear and the logistic model. We see that the best model (lowest AIC and BIC) is provided by the two-component model with a homogeneous relative risk estimate of $\exp (0.61)=1.84$ for the log-linear mixture model, which is not far off the estimate we have for the log-linear model with baseline heterogeneity and a homogeneous effect given in Table 2. For the logistic mixture model, the preferred model is also a two-component model with a homogeneous odds ratio estimate of $\exp (0.72) = 2.05$. Detailed results of the two-component mixture models are given in Table 5.

**Table 4:** Likelihoods, AIC and BIC, mean and variance of the mixing distribution for the fitted mixture models in the example.

Model	S	Log-likelihood	AIC	BIC	$\hat{\bar{\beta }}$	$\hat{\tau }^2$
Log-linear with effect heterogeneity	1	$-$57.70	119.30	120.90	0.63	0.00
	2	$-$37.30	84.50	88.40	0.51	0.02
	3	$-$36.50	88.90	95.10	0.73	0.22
Log$-$linear without effect heterogeneity	1	$-$57.70	119.30	120.90	0.63	0.00
	2	$-$37.40	82.80	85.90	0.61	0.00
	3	$-$37.10	86.20	90.90	0.60	0.00
Logistic with effect heterogeneity	1	$-$61.70	127.50	129.00	0.71	0.00
	2	$-$37.50	84.90	88.80	0.59	0.04
	3	$-$36.60	89.10	95.30	0.81	0.23
Logistic without effect heterogeneity	1	$-$61.70	127.50	129.00	0.71	0.00
	2	$-$37.80	83.60	86.70	0.72	0.00
	3	$-$37.40	86.90	91.50	0.71	0.00

### Model Estimation

All model fitting and analysis were conducted using R (R Core Team, 2020). Mixed models were fitted using the lme4 package (Bates et al., 2015). For models with both baseline and effect heterogeneity, a warning indicated that convergence could not be obtained with the default settings of the glmer-function, and thus, the argument control=glmerControl(optimizer="bobyqa",optCtrl=list(maxfun=2e5)) was added when fitting these models. Mixture models were fitted using the flexmix package (Grün & Leisch, 2007, 2008), which uses the EM algorithm to fit finite mixtures of generalised linear regressions. Specifically, we used the function stepFlexmix, which fits the model repeatedly for different numbers of classes and returns the maximum likelihood solution for each. For starting values, observations were allocated randomly to the initial classes for each run of the algorithm. This was repeated a number of times to achieve independence of estimates from starting values. The number of repetitions for this process was set to nrep = 10, since model estimation did not improve further for higher numbers of repetitions.

**Table 5:** Parameter estimates of weights, intercepts and slopes in the two classes mixture model.

Model	Class *s*	$\hat{q}_s$	$\hat{\alpha }_s$	$\hat{\beta }_s$
Log-linear with effect heterogeneity	1	0.62	$-$3.24	0.41
	2	0.38	$-$2.01	0.68
Log-linear without effect heterogeneity	1	0.62	$-$3.37	0.61
	2	0.38	$-$1.96	0.61
Logistic with effect heterogeneity	1	0.62	$-$3.21	0.44
	2	0.38	$-$1.86	0.84
Logistic without effect heterogeneity	1	0.62	$-$3.40	0.72
	2	0.38	$-$1.78	0.72

## Simulation Study

To assess the performance of nonparametric mixture models for meta-analysis with and without effect heterogeneity, we conducted two simulation studies: one simulation study for which the selection of simulation parameters was inspired by the example which is described in Sect. 1 and an additional simulation study with a larger number of conditions for which parameter values were varied systematically. In the following, we give a detailed description of the first of these two simulation studies. We will then conclude this section with a short summary of the second simulation study. A detailed description the second simulation study is available in the supplementary material of this article. Both simulation studies were implemented in R (R Core Team, 2020) and run on the computing cluster PALMA II (https://www.uni-muenster.de/ZIV/Technik/Server/HPC.html) at the University of Münster. Computations were parallelised using the *doParallel* package (Microsoft Corporation & Steve Weston, 2020).

### Data Generation

The simulation conditions under which the data for our first simulation study were generated based on the results from the analysis of the example in Sect. 1: specifically, we designed a baseline condition in which observations from $k = 8$ studies with an average sample size of 60 per study and group were generated from two classes (i.e. $S = 2$). We decided to include further simulation conditions with either a larger number of studies ($k = 50$) or a larger average sample size per group (${\bar{n}}_{ij} = 600$), or both. Each of these four simulation conditions was implemented with heterogeneous effects (i.e. $\beta _1 \ne \beta _2$, conditions 1–4) and with homogeneous effects (i.e. $\beta _1 = \beta _2$, conditions 5–8). All conditions are summarised in Table 6. The parameter values for $q_s$, $\alpha _s$ and $\beta _s$, $s = 1,2$, that we used in our simulation were chosen to mirror the estimates obtained from the mixture models estimated for the example (compared to Table 5). For each condition, 5500 replications were generated. The data for each replication were simulated as follows. First, the class *s* of each study was sampled from a $Bi(1, q_1)$ distribution, with $q_1 = 0.62$. Second, the sample size $n_{ij}$ for each group within a study was sampled from a $Po({\bar{n}}_{ij})$ distribution. Then, two separate data sets were generated: for the first data set, the parameter estimates $\hat{\alpha }_s$ and $\hat{\beta }_s$ of the log-linear mixture model were used to generate the observations for each study. For the second data set, observations were generated using the estimates $\hat{\alpha }_s$ and $\hat{\beta }_s$ of the logistic mixture model. This was necessary since strict effect homogeneity ($\beta _1 = \beta _2$) could not be obtained simultaneously for the log relative risk and the log odds ratio. By generating separate data sets, we ensured that for the first data set, effect heterogeneity was present for conditions 1–4 and effects were homogeneous for conditions 5–8 in terms of the log relative risk, while for the second data set, this was the case in terms of the log odds ratio. Thus, for conditions 1–4, the values for $\alpha _s$ and $\beta _s$ used in the simulation were obtained from their respective estimates from the log-linear mixture model with heterogeneous effects for the first data set and from the logistic mixture model with heterogeneous effects for the second data set. For conditions 5–8, $\alpha _s$ and $\beta _s$ were obtained from their respective estimates from the log-linear mixture model with homogeneous effects for the first data set and from the logistic mixture model with homogeneous effects for the second data set. Finally, for each data set, the observations for each group within each study were drawn from a $Bi(n_{ij}, p_{j,s})$ distribution, where $p_{j,s}$ was determined from $\alpha _s$ and $\beta _s$.

### Model Fitting

For each simulated meta-analysis, log-linear mixture models with and without effect heterogeneity as well as logistic mixture models with and without effect heterogeneity were fitted with $S = 1, S= 2$ and $S= 3$ classes, resulting in 2 (log-linear/logistic) $\times 2$ (effect heterogeneity/effect homogeneity) $\times 3$ (1/2/3 classes) $= 12$ models. Note, however, that the models with $S = 1$ with and without effect heterogeneity are identical, thus reducing the number of models to be evaluated to 10. The first data set (see above) was used to estimate the log-linear mixture models, while the second data set was used to fit the logistic mixture models. Just like for the example, the mixture models were fitted with the flexmix package using the stepFlexmix function with nrep = 10.

### Performance Evaluation

Model performance was evaluated in terms of model selection and parameter estimation. Log-linear and logistic mixture models were evaluated separately. Regarding model selection, the Akaike Information criterion (AIC) and Bayesian Information Criterion (BIC) were used to determine the preferred model, thereby taking into account both model fit and model complexity. The AIC and BIC are widely used criteria for model selection (Burnham & Anderson, 2002; Konishi & Kitagawa, 2008). Vrieze (2012) compares AIC and BIC in latent variable models and points out that the BIC consistently chooses the true model if it is among the candidate models considered.

Here, we first evaluated how often the model which was specified correctly in terms of effect heterogeneity and number of classes was preferred by AIC and BIC, respectively. Then, parameter estimation was evaluated in terms of mean, median and standard deviation of $\hat{\bar{\beta }}$ and $\hat{\tau }^2$.

### Simulation Results

Before the simulation results were calculated, we excluded trials in which one of the following warnings had occurred: “glm.fit: fitted probabilities numerically 0 or 1 occurred”, “glm.fit: algorithm did not converge”. A total of 82 simulation trials belonging to the first condition were excluded from the analysis, one trial was excluded in the second condition, 120 trials were excluded in the fifth condition, and two trials were excluded in the sixth condition. In the other conditions, no trials were excluded.

Table 7 summarises the results with regard to model selection. In the second column, the numbers of simulation trials which remained after exclusion are given for each condition. In the third and fourth columns, the relative number of simulation trials is displayed in which the log-linear mixture model which was correctly specified in terms of both the number of classes and effect size heterogeneity (yes/no) was preferred by the AIC and BIC, respectively. The same figures are given for the logistic mixture models in columns five and six. Model selection performance was quite variable for conditions where the true effect was heterogeneous (condition 1–4): for the first and second simulation conditions, the correctly specified model was favoured by the AIC and BIC in an unsatisfactorily low number of simulation trials for both the log-linear and the logistic mixture model. In the third condition, selection performance seems entirely satisfactory only for the logistic model, while in the fourth condition, the correct model was almost always favoured by both fit indices and for both types of mixture models.

Tables 8 and 9 provide information on the relative frequencies of each model being favoured by the AIC and BIC, respectively, per condition and separately for log-linear and logistic mixture models. From these figures, it becomes apparent that both AIC and BIC performed well in selecting the correct number of classes (i.e. $S = 2$), but often mistakenly favoured a model with homogeneous effects instead of a model with heterogeneous effects. For conditions where the true effect was homogeneous (i.e. $\beta _1 = \beta _2$, conditions 5–8), Table 7 reveals that both AIC and BIC performed satisfactorily. However, the BIC clearly outperformed the AIC, achieving almost perfect selection performance in conditions with a larger number of studies (i.e. conditions 6 and 8).

**Table 6:** Conditions used in the design of the simulation.

Condition	Homogeneous ($\beta _1 = \beta _2$)	*k*	${\bar{n}}_{ij}$	Data set	$\alpha _1$	$\alpha _2$	$\beta _1$	$\beta _2$
1	No	8	60	1	$-$3.24	$-$2.01	0.41	0.68
2	No	50	60	1	$-$3.24	$-$2.01	0.41	0.68
3	No	8	600	1	$-$3.24	$-$2.01	0.41	0.68
4	No	50	600	1	$-$3.24	$-$2.01	0.41	0.68
5	Yes	8	60	1	$-$3.37	$-$1.96	0.61	0.61
6	Yes	50	60	1	$-$3.37	$-$1.96	0.61	0.61
7	Yes	8	600	1	$-$3.37	$-$1.96	0.61	0.61
8	Yes	50	600	1	$-$3.37	$-$1.96	0.61	0.61
1	No	8	60	2	$-$3.21	$-$1.86	0.44	0.84
2	No	50	60	2	$-$3.21	$-$1.86	0.44	0.84
3	No	8	600	2	$-$3.21	$-$1.86	0.44	0.84
4	No	50	600	2	$-$3.21	$-$1.86	0.44	0.84
5	Yes	8	60	2	$-$3.40	$-$1.78	0.72	0.72
6	Yes	50	60	2	$-$3.40	$-$1.78	0.72	0.72
7	Yes	8	600	2	$-$3.40	$-$1.78	0.72	0.72
8	Yes	50	600	2	$-$3.40	$-$1.78	0.72	0.72

**Table 7:** Proportions of correct model selection.

Condition	No. trials	Log-linear		Logistic	
		AIC	BIC	AIC	BIC
1	5418	0.21	0.14	0.26	0.19
2	5499	0.50	0.24	0.66	0.46
3	5500	0.63	0.54	0.81	0.77
4	5500	0.97	0.99	0.94	1.00
5	5380	0.81	0.87	0.79	0.87
6	5498	0.83	0.97	0.77	0.97
7	5500	0.83	0.89	0.80	0.88
8	5500	0.84	0.98	0.79	0.97

**Table 8:** Relative frequencies of models being favoured by AIC or BIC for log-linear mixture models.

Effect		Homogeneous			Heterogeneous	
Conditions	Criterion	$S = 1$	$S = 2$	$S = 3$	$S = 2$	$S = 3$
1	AIC	0.03	0.74	0.01	0.21	0.01
	BIC	0.03	0.82	0.01	0.14	0.00
2	AIC	0.00	0.46	0.01	0.50	0.02
	BIC	0.00	0.76	0.00	0.24	0.00
3	AIC	0.02	0.33	0.01	0.63	0.02
	BIC	0.02	0.43	0.00	0.54	0.01
4	AIC	0.00	0.00	0.00	0.97	0.03
	BIC	0.00	0.01	0.00	0.99	0.00
5	AIC	0.03	0.81	0.01	0.14	0.01
	BIC	0.03	0.87	0.01	0.09	0.00
6	AIC	0.00	0.83	0.01	0.14	0.02
	BIC	0.00	0.97	0.00	0.03	0.00
7	AIC	0.02	0.83	0.01	0.13	0.01
	BIC	0.02	0.89	0.01	0.08	0.00
8	AIC	0.00	0.84	0.01	0.14	0.01
	BIC	0.00	0.98	0.00	0.02	0.00

Results with respect to the estimation of $\bar{\beta }$ are given in Table 10 for the log-linear mixture models and Table 11 for the logistic mixture models: For each simulation condition, the tables contain the true value of $\bar{\beta }$ along with the mean, median and standard deviation of $\hat{\bar{\beta }}$ across simulation trials. Please note that for conditions 1–4, a model with a heterogeneous effect and $S = 2$ would be correctly specified, while for conditions 5–8, a model with a homogeneous effect and $S = 2$ would be correctly specified. Hence, the sixth column contains the results of the correctly specified model for conditions 1–4, while the fourth column contains the results of the correctly specified model for conditions 5–8.

**Table 9:** Relative frequencies of models being favoured by AIC or BIC for logistic mixture models.

Effect		Homogeneous			Heterogeneous	
Conditions	Criterion	$S = 1$	$S = 2$	$S = 3$	$S = 2$	$S = 3$
1	AIC	0.02	0.67	0.02	0.26	0.03
	BIC	0.02	0.77	0.01	0.19	0.01
2	AIC	0.00	0.24	0.02	0.66	0.08
	BIC	0.00	0.53	0.00	0.46	0.00
3	AIC	0.02	0.14	0.00	0.81	0.03
	BIC	0.02	0.20	0.00	0.77	0.01
4	AIC	0.00	0.00	0.00	0.94	0.06
	BIC	0.00	0.00	0.00	1.00	0.00
5	AIC	0.02	0.79	0.03	0.14	0.02
	BIC	0.02	0.87	0.01	0.09	0.01
6	AIC	0.00	0.77	0.04	0.15	0.04
	BIC	0.00	0.97	0.00	0.03	0.00
7	AIC	0.02	0.80	0.02	0.14	0.02
	BIC	0.02	0.88	0.01	0.09	0.01
8	AIC	0.00	0.79	0.03	0.14	0.03
	BIC	0.00	0.97	0.00	0.03	0.00

Since the results were similar for the log-linear and logistic mixture models, we will describe them simultaneously. In general, $\bar{\beta }$ was estimated with a low mean and median bias, in particular by the model which was correctly specified, for all but the first condition. In the first condition, there was a slight positive bias in the estimation of $\bar{\beta }$, even for the correctly specified model. However, it should be noted that this condition was particularly challenging since it was characterised by both a low number of studies and a small sample size along with the presence of effect heterogeneity. In conditions where the true effect was heterogeneous, models in which the effect was specified as homogeneous overestimated the true $\bar{\beta }$ on average. However, in conditions with a truly homogeneous effect, bias in the estimation of $\bar{\beta }$ was low even if a model with heterogeneous treatment effects was specified. The only exception of this can be found in condition 5, where a model with $S = 3$ classes and heterogeneous effects on average overestimated $\bar{\beta }$. With regard to the standard deviation of $\hat{\bar{\beta }}$, reasonably small standard deviations were obtained in conditions with large numbers of studies and large sample sizes (i.e. conditions 4 and 8) for all models considered. In conditions in which either the sample size or the number of studies was small (i.e. conditions 2, 3, 6 and 7), standard deviations were notably larger, but still acceptable for all models. Large standard deviations were obtained in conditions in which both the number of studies and the sample sizes were small (i.e. conditions 1 and 5), in particular for the models with heterogeneous effects.

**Table 10:** Log-linear mixture model: estimation of $\bar{\beta }$.

Effect		Homogeneous			Heterogeneous	
Condition	Value	$S = 1$	$S = 2$	$S = 3$	$S = 2$	$S = 3$
1 (True $\bar{\beta } =$ 0.51)	Mean $\hat{\bar{\beta }}$	0.60	0.60	0.60	0.53	0.72
	Median $\hat{\bar{\beta }}$	0.60	0.60	0.60	0.53	0.56
	SD $\hat{\bar{\beta }}$	0.21	0.21	0.21	0.40	1.39
2 (True $\bar{\beta } =$ 0.51)	Mean $\hat{\bar{\beta }}$	0.60	0.60	0.60	0.51	0.53
	Median $\hat{\bar{\beta }}$	0.60	0.60	0.60	0.51	0.52
	SD $\hat{\bar{\beta }}$	0.08	0.08	0.08	0.10	0.16
3 (True $\bar{\beta } =$ 0.51)	Mean $\hat{\bar{\beta }}$	0.59	0.59	0.59	0.51	0.51
	Median $\hat{\bar{\beta }}$	0.60	0.60	0.60	0.52	0.52
	SD $\hat{\bar{\beta }}$	0.08	0.08	0.08	0.09	0.09
4 (True $\bar{\beta } =$ 0.51)	Mean $\hat{\bar{\beta }}$	0.60	0.60	0.60	0.51	0.51
	Median $\hat{\bar{\beta }}$	0.60	0.60	0.60	0.51	0.51
	SD $\hat{\bar{\beta }}$	0.03	0.03	0.03	0.04	0.04
5 (True $\bar{\beta } =$ 0.61)	Mean $\hat{\bar{\beta }}$	0.62	0.62	0.62	0.65	0.91
	Median $\hat{\bar{\beta }}$	0.61	0.61	0.61	0.62	0.66
	SD $\hat{\bar{\beta }}$	0.21	0.20	0.20	0.46	1.54
6 (True $\bar{\beta } =$ 0.61)	Mean $\hat{\bar{\beta }}$	0.61	0.61	0.61	0.61	0.64
	Median $\hat{\bar{\beta }}$	0.61	0.61	0.61	0.61	0.62
	SD $\hat{\bar{\beta }}$	0.08	0.08	0.08	0.10	0.18
7 (True $\bar{\beta } =$ 0.61)	Mean $\hat{\bar{\beta }}$	0.61	0.61	0.61	0.61	0.61
	Median $\hat{\bar{\beta }}$	0.61	0.61	0.61	0.61	0.61
	SD $\hat{\bar{\beta }}$	0.07	0.06	0.06	0.08	0.08
8 (True $\bar{\beta } =$ 0.61)	Mean $\hat{\bar{\beta }}$	0.61	0.61	0.61	0.61	0.61
	Median $\hat{\bar{\beta }}$	0.61	0.61	0.61	0.61	0.61
	SD $\hat{\bar{\beta }}$	0.03	0.02	0.02	0.03	0.03

Finally, Table 12 displays the results with regard to the estimation of $\tau ^2$. For each condition, the true value of $\tau ^2$ is given along with the mean and median value of $\hat{\tau }^2$ across simulation trials. When the correct number of classes was specified (i.e. $S = 2$), $\tau ^2$ was estimated with a low mean and median bias for conditions with a relatively large number of studies ($k = 50$) or a relatively large sample size (${\bar{n}}_{ij} = 600$), both when true heterogeneity was present (conditions 2, 3 and 4) and when it was absent (conditions 6, 7 and 8). When $S = 3$ classes were specified, heterogeneity was on average overestimated in the second and sixth conditions despite a large number of studies. In conditions where a low number of studies were combined with a small sample size (conditions 1 and 5), mean bias of $\hat{\tau }^2$ was particularly large and indicates an overestimation of heterogeneity. The fact that the median bias was smaller suggests that the large mean values of $\hat{\tau }^2$ were caused by a few outliers. In the respective conditions, we also obtained extremely large standard deviations of $\hat{\tau }^2$. In order to examine whether these issues were caused by few extreme outliers or whether they mirrored general problems in the estimation of $\tau ^2$, we computed several quantiles of the empirical distribution of $\hat{\tau }^2$. The results of these computations can be found in the online supplement. In short, we found that for log-linear and logistic models with three components, as many as $10 \%$ of all simulation replications yielded unrealistically large values of $\hat{\tau }^2$ in conditions with small numbers of studies and small sample sizes. In the same conditions, such estimation problems were less pronounced, but still evident for log-linear and logistic models with two components. For conditions with small sample sizes, these problems were restricted to models with three components, and for all other conditions, they were less evident. However, it should be noted that by unrealistically large values, we refer to values between about 8 and about 2904. Even more conditions tended to be affected by large numbers of simulation replications in which $\hat{\tau }^2$ was large compared to the true value of $\tau ^2$.

**Table 11:** Logistic mixture model: estimation of $\bar{\beta }$.

Effect		Homogeneous			Heterogeneous	
Condition	Value	$S = 1$	$S = 2$	$S = 3$	$S = 2$	$S = 3$
1 (True $\bar{\beta } =$ 0.59)	Mean $\hat{\bar{\beta }}$	0.66	0.70	0.70	0.60	0.72
	Median $\hat{\bar{\beta }}$	0.67	0.71	0.71	0.60	0.63
	SD $\hat{\bar{\beta }}$	0.24	0.25	0.25	0.29	1.21
2 (True $\bar{\beta } =$ 0.59)	Mean $\hat{\bar{\beta }}$	0.67	0.71	0.71	0.59	0.62
	Median $\hat{\bar{\beta }}$	0.67	0.71	0.71	0.59	0.60
	SD $\hat{\bar{\beta }}$	0.09	0.09	0.09	0.11	0.18
3 (True $\bar{\beta } =$ 0.59)	Mean $\hat{\bar{\beta }}$	0.66	0.69	0.69	0.59	0.59
	Median $\hat{\bar{\beta }}$	0.67	0.70	0.70	0.59	0.60
	SD $\hat{\bar{\beta }}$	0.10	0.10	0.10	0.11	0.11
4 (True $\bar{\beta } =$ 0.59)	Mean $\hat{\bar{\beta }}$	0.67	0.71	0.71	0.59	0.59
	Median $\hat{\bar{\beta }}$	0.67	0.71	0.71	0.59	0.59
	SD $\hat{\bar{\beta }}$	0.04	0.04	0.04	0.04	0.04
5 (True $\bar{\beta } =$ 0.72)	Mean $\hat{\bar{\beta }}$	0.69	0.72	0.73	0.74	0.96
	Median $\hat{\bar{\beta }}$	0.69	0.72	0.72	0.73	0.77
	SD $\hat{\bar{\beta }}$	0.23	0.23	0.23	0.34	1.30
6 (True $\bar{\beta } =$ 0.72)	Mean $\hat{\bar{\beta }}$	0.68	0.72	0.72	0.72	0.76
	Median $\hat{\bar{\beta }}$	0.68	0.72	0.72	0.72	0.74
	SD $\hat{\bar{\beta }}$	0.09	0.09	0.09	0.11	0.21
7 (True $\bar{\beta } =$ 0.72)	Mean $\hat{\bar{\beta }}$	0.68	0.72	0.72	0.72	0.72
	Median $\hat{\bar{\beta }}$	0.68	0.72	0.72	0.72	0.72
	SD $\hat{\bar{\beta }}$	0.07	0.07	0.07	0.08	0.09
8 (True $\bar{\beta } =$ 0.72)	Mean $\hat{\bar{\beta }}$	0.68	0.72	0.72	0.72	0.72
	Median $\hat{\bar{\beta }}$	0.68	0.72	0.72	0.72	0.72
	SD $\hat{\bar{\beta }}$	0.03	0.03	0.03	0.03	0.03

### Summary of the Second Simulation Study

In the second of our simulation studies, we simulated conditions with two and three components and varied (i) the number of studies, (ii) the size of samples within studies, (iii) the component weights $q_s$, (iv) the component baseline probabilities $p_{0,s}$, and (v) the value of $\tau ^2$. The values which were chosen for these parameters are given in Table 13.

Just like for the first simulation study, the results of the second simulation study were evaluated in terms of model selection performance using the AIC and BIC, as well as performance with regard to the estimation of $\bar{\beta }$ and $\tau ^2$. Furthermore, we evaluated the estimation of $\hat{\beta }_s$ for the correctly specified model. We found that both log-linear and logistic mixture models almost always performed well in terms of model selection and parameter estimation when sample sizes within studies were large. In these situations, the BIC yielded better results in terms of model selection than the AIC. For smaller sample sizes, model selection performance depended on the number of studies and on how well the components were separated in terms of the difference between the baseline probabilities $p_{0,s}$ or in terms of the component effects $\beta _s$. Almost unbiased parameter estimates with small variances could be achieved in conditions with small sample sizes when either effects were truly homogeneous, or when the correctly specified model was selected in conditions with heterogeneous effects and the number of studies was large. In the supplementary material, we give detailed descriptions of the design and the results of this simulation study.

**Table 12:** Mixture models estimated with heterogeneous effect: estimation of $\tau ^2$.

Condition	Value	Log-linear		Logistic	
		$$s = 2$$	$$s = 3$$	$$ s = 2$$	$$s = 3$$
1	True $\tau ^2$	0.02	0.02	0.04	0.04
	Mean $\hat{\tau }^2$	0.43	9.44	0.14	7.57
	Median $\hat{\tau }^2$	0.03	0.14	0.05	0.19
	SD $\hat{\tau }^2$	5.37	38.61	1.12	21.32
2	True $\tau ^2$	0.02	0.02	0.04	0.04
	Mean $\hat{\tau }^2$	0.02	0.28	0.05	0.37
	Median $\hat{\tau }^2$	0.02	0.04	0.04	0.08
	SD $\hat{\tau }^2$	0.03	1.46	0.04	1.61
3	True $\tau ^2$	0.02	0.02	0.04	0.04
	Mean $\hat{\tau }^2$	0.02	0.03	0.04	0.05
	Median $\hat{\tau }^2$	0.02	0.02	0.03	0.04
	SD $\hat{\tau }^2$	0.02	0.03	0.03	0.04
4	True $\tau ^2$	0.02	0.02	0.04	0.04
	Mean $\hat{\tau }^2$	0.02	0.02	0.04	0.04
	Median $\hat{\tau }^2$	0.02	0.02	0.04	0.04
	SD $\hat{\tau }^2$	0.01	0.01	0.01	0.01
5	True $\tau ^2$	0.00	0.00	0.00	0.00
	Mean $\hat{\tau }^2$	0.50	10.33	0.16	7.78
	Median $\hat{\tau }^2$	0.02	0.12	0.03	0.15
	SD $\hat{\tau }^2$	6.37	59.29	2.37	21.99
6	True $\tau ^2$	0.00	0.00	0.00	0.00
	Mean $\hat{\tau }^2$	0.01	0.33	0.01	0.42
	Median $\hat{\tau }^2$	0.00	0.02	0.00	0.04
	SD $\hat{\tau }^2$	0.01	1.58	0.01	1.77
7	True $\tau ^2$	0.00	0.00	0.00	0.00
	Mean $\hat{\tau }^2$	0.01	0.02	0.01	0.02
	Median $\hat{\tau }^2$	0.00	0.01	0.00	0.01
	SD $\hat{\tau }^2$	0.01	0.02	0.01	0.03
8	True $\tau ^2$	0.00	0.00	0.00	0.00
	Mean $\hat{\tau }^2$	0.00	0.00	0.00	0.01
	Median $\hat{\tau }^2$	0.00	0.00	0.00	0.00
	SD $\hat{\tau }^2$	0.00	0.01	0.00	0.01

**Table 13:** Simulation parameters of the second simulation study.

Parameter	Values (conditions with $S = 2$)	Values (conditions with $S = 3$)
*k*	15, 25, 40	15, 25, 40
$n_0$	50, 500	50, 500
$\tau ^2$	0, 0.36	0, 0.36
$q_s$	$q_1 \in \{0.3, 0.5, 0.7\}$	$q_1 = q_2 = q_3 = 1/3$
$p_{0,s}$	$(p_{0,1}, p_{0,2})\in \{(0.05, 0.1), (0.1, 0.05), (0.05, 0.2), (0.2, 0.05)\}$	$(p_{0,1}, p_{0,2}, p_{0,3})\in \{(0.05, 0.1, 0.2), (0.05, 0.2, 0.1), (0.1, 0.05, 0.2), (0.1, 0.2, 0.05), (0.2, 0.05, 0.1), (0.2, 0.1, 0.05)\}$

## Discussion and Conclusions

In this paper, we presented alternatives to conventional two-stage approaches for meta-analysis from the family of generalised linear mixed models and nonparametric mixture models. These alternatives overcome the shortcomings of conventional inverse variance-weighted two-stage models, where studies with zero-counts cannot be included when adding a smoothing constant. In addition, they allow separate modelling of baseline heterogeneity and effect heterogeneity, while the Mantel–Haenszel approach rests on the assumption of homogeneity. In contrast with the log-linear and logistic mixed models presented, nonparametric mixture models do not require specification of the random effects distribution. In particular, these models avoid the assumption of a normal random effects distribution, as this assumption cannot be easily investigated. This is also correct for alternative random effect distributions such as the Gamma or the Beta distribution, as these are mainly chosen for mathematical convenience as they allow closed form solutions for the marginal integrals (which is not the case for the normal random effect distribution). The nonparametric approach circumvents this issue entirely.

We return to the issue that consideration of the study level variation can be crucial, and simply pooling the data across studies would have considerable confounding potential. Let us consider the following synthetic example. We generate two types of studies. In study type A, we have a baseline risk of 0.5 with 100 persons at risk in the control group. Ten persons are at risk in the treatment group where the risk is also 0.5, so that the risk ratio is 1. In study type B, we have a baseline risk of 0.1 with 10 persons at risk in the control group. One hundred persons are at risk in the treatment group for which the risk is also 0.1. In both studies, the risk ratio is 1; in other words, there is no effect present. We generate 20 studies of type A and 20 of type B, to keep the scenario realistic. Common sense would tell us, independent of whether you favour the arm-based or contrast-based approach, that any decent analysis would come to the conclusion of no effect. The pooled analysis provides a risk ratio of 0.28 with 95% confidence interval (0.25, 0.32), so a clear and significant effect. Including the baseline parameter as a fixed main effect in the Poisson model yields a risk ratio (CI) of 1.01 (0.83, 1.22) and the Mantel–Haenszel estimate is 1.01 (0.83, 1.22), identical to the former up to two decimal places. Including the baseline parameter as a random effect yields a risk ratio of 0.83 (0.67,1.02), also avoiding the strong confounding effect of the unadjusted effect. Of course, the case study was constructed to make this point, by choosing strong baseline risk variation and highly unbalanced intervention and control groups in the studies, which are typical conditions for the occurrence of confounding. Another possibility would be to eliminate the baseline nuisance parameter prior to any further modelling. This approach is laid out in detail in Böhning et al. (2008) for the profile likelihood.

We consider the risk ratio (or odds ratio) in our setting. Although the risk difference has considerable benefits and is indeed statistically easier to treat, it is not without reason that the statistical (and primarily the clinical) community often favours the risk (odds) ratio. One of the major reasons is that the latter is invariant towards study duration (making the reasonable assumption that duration is identical in intervention and control groups), whereas this is not the case for the risk difference. In other words, the risk (odds) ratio is a relative effect measure, whereas the risk difference is an absolute one. Alternative meta-analytic approaches for the risk and odds ratio were laid out by Bonett and Price (2014, 2015). They do this, at least in the way they measure effect, in a similar way as the conventional meta-analytic approaches presented here. This means that for each study the effect measure is calculated before it is further analysed. Here we do not follow this scheme but rather work directly with the accrued counts of cases among those under risk, in the groups to be compared; in other words, we are working with four cell counts per study. We find this the most appropriate approach in situations experiencing high sparsity including the occurrence of zero studies. We quote from the much appreciated work of Alan Agresti (2013: 507):

A challenging situation for meta-analysis is when the outcome of interest has very low probability. Some tables may have empty cells for one or both treatments.

Bonett and Price (2015:395) develop their approach with the help of smoothing constants (1/4 in the case of the RR, 1/2 in the case of the OR); otherwise, the effect measures would not be defined for some studies. Now, there is considerable concern on the use of smoothing constants as they might add bias of considerable size. We refer to the work of Sweeting et al. (2004), Bradburn et al. (2007), Kuss (2015), or Chang and Hoaglin (2017). Indeed, the Mantel–Haenszel estimate for the RR is 1.86 (close to the Poisson regression model including study as factor), which is quite different from 1.38 reached with the help of smoothing constants, underlining the concerns of adding smoothing values. We think that Poisson and Binomial models with their generalisations are the appropriate approach to deal with these situations as the occurrence of zero counts are part of their natural domain, whereas effect estimates per study are not permissible and need special attentions (such as adding pseudo-values). It is of course a cause of concern that standard errors might be too small when using weighted or unweighted mean marginal estimates. For example, the variance of the Mantel–Haenszel estimate is constructed using the formula developed in Greenland and Robbins (1985), which is done under the assumption of homogeneity of effect. This is precisely why we think it is more appropriate to incorporate heterogeneity into the modelling which can be done using mixed Poisson (or Binomial) regression or using nonparametric mixture models as suggested here.

Potential applications of the models presented could be meta-analyses of randomised controlled trials (RCTs) comparing a psychological intervention with control conditions with outcomes such as complete recovery, remission, response, dropout rates, acceptability or adverse events. For such meta-analyses, it can be beneficial to separately model baseline heterogeneity and effect heterogeneity: RCTs differ in their types of control conditions (see, for example, the case study presented above), which makes it reasonable to expect heterogeneity in the baseline population. Also, although inclusion criteria for the treatment condition are often more narrowly defined, there are still various methodological aspects which can differ across studies, such as treatment duration and intensity, treatment material, and whether patients with comorbidities were included or not, which potentially introduces effect heterogeneity. Both generalised linear mixed models and nonparametric mixture models allow for such heterogeneity to be accounted for.

Furthermore, these models can easily be fitted using common statistical software such as R (R Core Team, 2020). While software packages for generalised linear mixed models are commonly used in psychology (e.g. *lme4*), software packages for nonparametric mixture models (such as the *flexmix* package) do not enjoy a similar degree of popularity. Also, there is a lack of simulation studies comparing nonparametric mixture models to generalised linear mixed models. The latter were found to perform well in various simulation studies and for different meta-analytic settings (Beisemann et al., 2020; Jackson et al., 2017). The results from our simulation studies reveal that when the assumptions of the nonparametric mixture model are fulfilled and enough studies with reasonably large sample sizes are available, nonparametric mixture models provide good estimates of both the pooled effect and heterogeneity. Furthermore, we found that established criteria, such as the AIC and the BIC, can be applied for model selection. When the aforementioned requirements with regard to sample sizes and numbers of studies are met, these criteria perform well both in selecting the correct number of components and in selecting the model which is correctly specified in terms of effect heterogeneity. Of course, our simulation studies were not without limitations: First, the data-generating model which we used in our simulation study requires the specification of a large number of parameters, which translates to an extremely large number of simulation conditions for which these models can and potentially should be investigated. In fact, the nonparametric nature of these models makes it harder to establish a simulation design which allows for a comprehensive, yet efficient investigation. Furthermore, a simulation study is not suited to determine the conditions under which nonparametric mixture models are useful, since in practical applications, their usefulness critically depends on the interpretability of the component results. In this respect, nonparametric mixture models might serve as a complement to generalised linear mixed models when either the assumption of a normal random-effects distribution is unlikely to be fulfilled or when the components are of theoretical interest.

In conclusion, we encourage utilising the flexibility of generalised linear mixed models and nonparametric mixture models in rare events meta-analyses both when conducting a meta-analysis and in future research on meta-analytical models.

## Supplementary Information

Below is the link to the electronic supplementary material.

Supplementary file 1 (pdf 768 KB)
