# Supplementary material for: Estimation of Effect Heterogeneity in Rare Events Meta-Analysis
Source: Psychometrika. 2022 Feb 8;87(3):1081–102. doi: 10.1007/s11336-021-09835-5 (PMC9433364; doi:10.1007/s11336-021-09835-5)
Supplement: Supplementary file 1 — (pdf 768 KB) [file 11336_2021_9835_MOESM1_ESM.pdf]

SUPPLEMENT: ESTIMATION OF EFFECT HETEROGENEITY IN RARE EVENTS META-ANALYSIS

Heinz Holling, Katrin Jansen, Walailuck Böhning, Dankmar Böhning, Susan Martin and Patarawan Sangnawakij

In this supplement, we provide additional material for the article "Estimation of effect heterogeneity in rare events meta-analysis". In the first section, we present tables of quantiles of the empirical distribution of $\hat{\tau}^2$ from the simulation study which was described in the main article. In the second section, we describe and evaluate an additional simulation study where we assess the performance of the finite mixture models described in the main article, namely the log-linear and the logistic mixture model with and without effect heterogeneity, for a larger number of simulation conditions.

Contents

S1 Quantiles of the empirical distribution of $\hat{\tau}^2$ . . . S2

S2 Additional simulation study . . . S5

S2.1 Simulation conditions . . . S5
S2.2 Data generation . . . S7
S2.3 Model fitting . . . S7
S2.4 Performance measures . . . S7
S2.5 Simulation results . . . S8
S2.5.1 Model selection . . . S8
S2.5.2 Estimation of $\bar{\beta}$ . . . S10
S2.5.3 Estimation of τ^2 . . . S11
S2.6 Estimation of β_s . . . S13
S2.7 Conclusion . . . S13

S1. Quantiles of the empirical distribution of $\hat{\tau}^2$

Table S1 displays minimum, maximum and different quantiles of the empirical distribution of $\hat{\tau}^2$ of the log-linear mixture models with heterogeneous effects and 2 or 3 components (i.e., $S = 2$ or $S = 3$), while Table S2 displays the same figures for the logistic mixture models with heterogeneous effects and 2 or 3 components.

TABLE S1.
Quantiles of the empirical distribution of $\hat{\tau}^2$ of the log-linear mixture model

Condition	S	min	q10	q20	q30	q40	q50	q60	q70	q80	q90	max
1	2	0.0000	0.0009	0.0040	0.0093	0.0186	0.0300	0.0493	0.0763	0.1173	0.2026	112.1637
	3	0.0000	0.0058	0.0240	0.0506	0.0853	0.1384	0.2150	0.3482	0.6992	44.9656	1288.8058
2	2	0.0000	0.0010	0.0038	0.0075	0.0119	0.0173	0.0230	0.0302	0.0412	0.0579	0.2056
	3	0.0000	0.0056	0.0136	0.0230	0.0328	0.0447	0.0631	0.0892	0.1410	0.2566	56.8143
3	2	0.0000	0.0014	0.0043	0.0077	0.0113	0.0150	0.0195	0.0251	0.0324	0.0450	0.1310
	3	0.0000	0.0034	0.0083	0.0128	0.0173	0.0229	0.0287	0.0367	0.0464	0.0649	0.2328
4	2	0.0015	0.0090	0.0112	0.0133	0.0149	0.0166	0.0184	0.0204	0.0231	0.0270	0.0523
	3	0.0015	0.0104	0.0130	0.0154	0.0173	0.0193	0.0216	0.0245	0.0281	0.0337	0.0946
5	2	0.0000	0.0006	0.0029	0.0074	0.0135	0.0224	0.0354	0.0576	0.0912	0.1611	136.4584
	3	0.0000	0.0049	0.0188	0.0408	0.0719	0.1174	0.1918	0.3430	0.7610	32.7423	2904.3220
6	2	0.0000	0.0001	0.0005	0.0011	0.0020	0.0034	0.0055	0.0082	0.0125	0.0208	0.1059
	3	0.0000	0.0012	0.0042	0.0085	0.0147	0.0233	0.0379	0.0660	0.1277	0.2652	34.6683
7	2	0.0000	0.0001	0.0003	0.0007	0.0012	0.0020	0.0032	0.0050	0.0077	0.0131	0.1669
	3	0.0000	0.0002	0.0009	0.0021	0.0038	0.0062	0.0097	0.0149	0.0243	0.0437	0.1925
8	2	0.0000	0.0000	0.0000	0.0001	0.0002	0.0003	0.0005	0.0008	0.0012	0.0020	0.0102
	3	0.0000	0.0001	0.0002	0.0005	0.0008	0.0014	0.0023	0.0037	0.0072	0.0147	0.0701

TABLE S2.
Quantiles of the empirical distribution of $\hat{\tau}^2$ of the logistic mixture model

Condition	S	min	q10	q20	q30	q40	q50	q60	q70	q80	q90	max
1	2	0.0000	0.0016	0.0069	0.0162	0.0309	0.0483	0.0729	0.1099	0.1638	0.2665	55.5233
	3	0.0000	0.0138	0.0454	0.0870	0.1348	0.1936	0.2822	0.4202	0.7695	29.5040	149.7924
2	2	0.0000	0.0052	0.0127	0.0201	0.0283	0.0371	0.0464	0.0581	0.0736	0.0982	0.2676
	3	0.0000	0.0190	0.0347	0.0484	0.0646	0.0837	0.1059	0.1412	0.1987	0.3301	28.4596
3	2	0.0000	0.0066	0.0136	0.0201	0.0263	0.0335	0.0404	0.0491	0.0599	0.0783	0.2257
	3	0.0000	0.0108	0.0193	0.0270	0.0346	0.0433	0.0521	0.0625	0.0756	0.0957	0.3197
4	2	0.0077	0.0237	0.0278	0.0309	0.0339	0.0366	0.0398	0.0431	0.0471	0.0537	0.0867
	3	0.0083	0.0266	0.0308	0.0345	0.0379	0.0411	0.0444	0.0484	0.0535	0.0606	0.1057
5	2	0.0000	0.0007	0.0037	0.0088	0.0160	0.0271	0.0428	0.0664	0.1024	0.1768	128.5602
	3	0.0000	0.0088	0.0300	0.0600	0.0984	0.1549	0.2327	0.3735	0.7799	26.4567	157.4469
6	2	0.0000	0.0002	0.0006	0.0013	0.0024	0.0040	0.0063	0.0097	0.0152	0.0258	0.1527
	3	0.0000	0.0031	0.0081	0.0154	0.0254	0.0385	0.0580	0.0898	0.1646	0.3413	40.5588
7	2	0.0000	0.0001	0.0003	0.0008	0.0015	0.0025	0.0039	0.0059	0.0092	0.0153	0.1134
	3	0.0000	0.0003	0.0012	0.0028	0.0049	0.0080	0.0120	0.0178	0.0278	0.0486	0.3337
8	2	0.0000	0.0000	0.0001	0.0001	0.0002	0.0004	0.0006	0.0010	0.0014	0.0024	0.0158
	3	0.0000	0.0001	0.0004	0.0008	0.0014	0.0023	0.0037	0.0058	0.0099	0.0179	0.0652

S2. Additional simulation study

This section is structured as follows: First, we describe the design of the simulation study which was conducted and the performance measures which were used. Then, we evaluate the performance of the models investigated. Finally, we formulate a brief conclusion. The simulation study which we describe here was implemented in R (R Core Team, 2020) and run on the computing cluster PALMA II (https://www.uni-muenster.de/ZIV/Technik/Server/HPC.html) at the University of Münster. Computations were parallelized using the doParallel Package (Microsoft Corporation & Weston, 2020).

S2.1. Simulation conditions

S2.1 Simulation conditions

We investigated a total number of 216 simulation conditions. In 144 of these conditions, we generated the data from two components (i.e., $S = 2$). In the remaining 72 conditions, we generated the data from three components (i.e, $S = 3$). Different conditions were distinguished by the number of studies k, the control group sample size n_0, the component weights q_s ($s \in \{1, ..., S\}$), the component baseline probabilities $p_{0,s}$, and the true value of τ^2. The values of these parameters which were examined in the simulation study are displayed in Table S3. The values of k were inspired by a review of systematic reviews by Moher et al. (2007), and roughly reflect the 25% quantile, the median and the 75% quantile of their sample of Non-Cochrane reviews. The values of n_0, 50 and 500, were selected since $n_0 = 50$ mirrors the median number of subjects per group from a review of meta-analyses from the *Cochrane Database of Systematic Reviews* by Turner et al. (2012), while $n_0 = 500$ mirros the median number of subjects per group of the Non-Cochrane sample from the above-cited review by Moher et al. (2007). The values of τ^2 reflect conditions without and with moderate heterogeneity. Specifically, the value $\tau^2 = 0.36$ was obtained from a reanalysis of Cochrane reviews of adverse events conducted by Beisemann et al. (2020).

We only considered balanced groups, i.e., $n_1 = n_0$. Furthermore, we set $\bar{\beta} = 0$ for all conditions. For the simulation conditions with $S = 2$, the component probability of the second component can be obtained as $q_2 = 1 - q_1$. The values for β_1 and β_2 can then be obtained by the following equations:

$$\beta_1 = \bar{\beta} + \sqrt{(q_2/q_1)}\tau \quad (1)$$

and

Table S3.
Simulation parameters

Parameter	Values (conditions with $S = 2$)	Values (conditions with $S = 3$)
k	15, 25, 40	15, 25, 40
n_0	50, 500	50, 500
τ^2	0, 0.36	0, 0.36
q_s	$q_1 \in \{0.3, 0.5, 0.7\}$	$q_1 = q_2 = q_3 = 1/3$
$p_{0,s}$	$(p_{0,1}, p_{0,2}) \in$ $\{(0.05, 0.1), (0.1, 0.05),$ $(0.05, 0.2), (0.2, 0.05)\}$	$(p_{0,1}, p_{0,2}, p_{0,3}) \in$ $\{(0.05, 0.1, 0.2), (0.05, 0.2, 0.1),$ $(0.1, 0.05, 0.2), (0.1, 0.2, 0.05),$ $(0.2, 0.05, 0.1), (0.2, 0.1, 0.05)\}$

$$\beta_2 = \bar{\beta} - \sqrt{(q_1/q_2)}\tau \quad (2)$$

These are obtained as solutions to the equations

$$\bar{\beta} = \sum_{s=1}^{S} q_s \beta_s \quad (3)$$

and

$$\tau^2 = \sum_{s=1}^{S} q_s(\bar{\beta} - \beta_s)^2. \quad (4)$$

Note that the same equations with reversed signs (i.e., $\tilde{\beta}_1 = \bar{\beta} - \sqrt{(q_2/q_1)}\tau$ and $\tilde{\beta}_2 = \bar{\beta} + \sqrt{(q_1/q_2)}\tau$) would also be solutions to these equations. However, these alternative solutions are incorporated via the simulation design by (i) including the permutation of each pair $(p_{0,1}, p_{0,2})$ and (ii) including $\tilde{q_1} = 1 - q_1$ in the simulation design for each value of q_1.

For the simulation conditions with three components, there are no unique solutions for equations 3 and 4. Thus, we set $\beta_2 = 0$ and chose β_1 and β_3 such that equations 3 and 4 were fulfilled. In conditions with $\tau^2 = 0$, $\beta_1 = \beta_2 = \beta_3 = 0$. In conditions with $\tau^2 = 0.36$, $\beta_1 \approx 0.7348$ and $\beta_3 \approx -0.7348$.

S2.2. Data generation

S2.2 Data generation

For each condition, 5500 replications were generated. In each replication, meta-analytic data were generated with the following procedure:

First, the class s of each study was sampled: For conditions with $S = 2$, the class was sampled from a Bernoulli-distribution $\text{Bern}(q_1)$. For conditions with $S = 3$, the class was sampled from a multinomial distribution with parameters $n = 1$ and $(p_1, p_2, p_3) = (q_1, q_2, q_3)$. Then, two separate data sets were generated, one for the simulation study of log-linear mixture models, and one for the simulation study of logistic mixture models. The generation of two separate data sets was necessary to ensure that for both models, true heterogeneity matched the value of τ^2 of the respective simulation condition. Thus, for the first data set, the event probability in the treatment group was calculated based on the relative risk (RR), such that $p_{1,s} = \exp(\beta_s) \cdot p_{0,s}$. For the second data set, $p_{1,s}$ was calculated based on the odds ratio (OR), such that $p_{1,s} = y/(1 + y)$, with $y = (exp(\beta_s)\ p_{0,s})/(1 - p_{0,s})$. Finally, for each data set, the observations for each group within each study were drawn from a $\text{Bin}(n_j, p_{j,s})$ distribution, where j is the group index (with $j = 0$: control group, $j = 1$: treatment group).

S2.3. Model fitting

S2.3 Model fitting

Just like for the simulation study described in the main article, we fitted log-linear and logistic mixture models with and without effect heterogeneity. For conditions with $S = 2$, models with $S = 1$, $S = 2$ and $S = 3$ components were fitted. Thus, in total five different models were fitted to each data set for these conditions (since models with and without effect heterogeneity with $S = 1$ are identical). For conditions with $S = 3$, models with $S = 4$ were fitted in addition to the models with up to three components, such that in total seven different models were fitted to each data set for these conditions. As described above, log-linear mixture models were fitted to the first data set, while logistic mixture models were fitted to the second data set. All models were fitted with the `flexmix` package (Grün and Leisch, 2008) using the `stepFlexmix` function with `nrep = 10`.

S2.4. Performance measures

S2.4 Performance measures

Performance was evaluated in terms of model selection and parameter estimation. All performance measures were calculated separately for log-linear and logistic mixture models. With regard to model selection, we first assessed the percentage of simulation replications in which the correct model was favoured by the Akaike Information Criterion (AIC) or the Bayesian Information Criterion (BIC) among all models which were fitted (see section "Model fitting"). Second, we calculated the percentage of simulation replications in which each

specific model (of the five models examined) was favoured by the AIC or BIC, respectively. This was done to further examine whether model selection criteria rather failed to recognize effect homogeneity/heterogeneity or rather selected the wrong number of components in simulation replications in which a misspecified model was favoured. To keep the supplement succinct, only the results with regard to the percentage of simulation replications in which the correct model was selected will be illustrated by figures. In addition, we briefly summarize the results with regard to which models were selected erroneously in the cases in which model selection failed. Please note that the full results can be obtained from the corresponding author on request.

Parameter estimation was evaluated in terms of mean bias, median bias, and the standard deviation of $\hat{\bar{\beta}}$ and $\hat{\tau}^2$. In addition, we visually inspected histograms of the estimates of β_s for $s = 1, ..., S$ for all simulation conditions. We will present a representative selection of these histograms in the final part of the results section. Further results on these within-component parameter estimates can be obtained from the corresponding author on request.

S2.5. Simulation results

S2.5 Simulation results

Before the simulation results were calculated, we excluded simulation replications in which one of the following warnings had occurred: "glm.fit: fitted probabilities numerically 0 or 1 occurred", "glm.fit: algorithm did not converge". For the log-linear model, no simulation replications had to be excluded. For the logistic model, the number of simulation replications which were excluded ranged from 0 to 653 across all 216 conditions.

S2.5.1. Model selection

Figure S1 shows the percentage of simulation replications in which the correct model was selected for conditions with $S = 2$. Results are displayed separately for the log-linear (red line) and the logistic model (blue line), and for both information criteria (AIC: solid line, BIC: dashed line). In the following, we briefly outline the most important results:

- In conditions with large sample sizes ($n_0 = 500$),
  - both information criteria performed well in terms of selecting the correct model, irrespective of the number of studies, the values of $p_{0,j}$ and the value of τ^2.
  - model selection performance was best for those conditions in which (i) the components were more separated in terms of the difference between $p_{0,1}$ and $p_{0,2}$ or (ii) effects were truly heterogeneous (i.e., $\tau^2 = 0.36$).
  - the BIC performed slightly better than the AIC in selecting the correct model.

- In conditions with small sample sizes ($n_0 = 50$),
  - model selection performance was severely impaired in many conditions, in particular for small numbers of studies, homogeneous effects ($\tau^2 = 0$) and when components were less separated in terms of the difference between $p_{0,1}$ and $p_{0,2}$.
  - model selection performance only reached a satisfactory level when components were more separated in terms of the difference between $p_{0,1}$ and $p_{0,2}$.
  - the AIC yielded better results in terms of selecting the correct model.
- In conditions with large sample sizes and in conditions in which the components were more separated in terms of the difference between $p_{0,1}$ and $p_{0,2}$, either a model with two components and heterogeneous effects or a model with three components and homogeneous effects was often chosen in the few trials in which the correct model was not selected (results not shown).
- In conditions with small sample sizes, small numbers of studies and when components were less separated in terms of the difference between $p_{0,1}$ and $p_{0,2}$, a model with one component was often selected instead of the correct model (results not shown).

Figure S2 shows the percentage of simulation replications in which the correct model was selected for conditions with $S = 3$. Results are displayed separately for the log-linear (red line) and the logistic model (blue line), and for both information criteria (AIC: solid line, BIC: dashed line). These were the most important results:

- In conditions with large sample sizes ($n_0 = 500$),
  - model selection performance was good for the majority of simulation conditions. The correct model was usually selected in about 75% of simulation replications, irrespective of the number of studies.
  - in simulation replications in which the correct model was not selected, a model with four components which was correctly specified in terms of effect heterogeneity was often selected erroneously. When effects were truly homogeneous (i.e., $\tau^2 = 0$), the AIC also sometimes favoured a model with three components but with heterogeneous effects (results not shown).
  - Usually, performance was better in conditions with true heterogeneity ($\tau^2 = 0.36$), with the exception of conditions with $p_{0,1} = 0.2$, $p_{0,2} = 0.05$, and $p_{0,3} = 0.1$, in which the correct model was only selected in around 50% of simulation replications. For these conditions, we found that a model with four components was often selected erroneously.
  - the BIC usually performed slightly better than the AIC in selecting the correct model.

- In conditions with small sample sizes ($n_0 = 50$),
  - model selection performance was usually unsatisfactory. Although performance improved with larger numbers of studies, the percentage of simulation replications in which the correct model was favoured was often below 50% even for conditions with $k = 40$.
  - often, a model with two components was favoured by the AIC and BIC instead of the correct model (results not shown).
  - the AIC usually performed notably better in selecting the correct model than the BIC, but still did not achieve a satisfactory selection performance in most conditions.

S2.5.2. Estimation of $\bar{\beta}$

Figures S3 to S8 illustrate the results with regard to the estimation of $\hat{\bar{\beta}}$ for conditions with $S = 2$. Figures S3-S5 depict mean bias, median bias and SD($\hat{\bar{\beta}}$) for the log-linear mixture models, respectively. Figures S6-S8 depict mean bias, median bias and SD($\hat{\bar{\beta}}$) for the logistic mixture models, respectively. Since results of log-linear and logistic mixture models were similar, we will describe them simultaneously. The most important results were the following:

- In conditions with homogeneous effects ($\tau^2 = 0$),
  - bias was negligible for all models investigated.
  - standard deviations of $\hat{\bar{\beta}}$ were small for all models without effect heterogeneity, and also for models with effect heterogeneity in conditions with large sample sizes (i.e., $n_0 = 500$).
  - standard deviations were larger for models with effect heterogeneity in conditions with small sample sizes (i.e., $n_0 = 50$), in particular in conditions with small numbers of studies or when components were less separated in terms of the difference between $p_{0,1}$ and $p_{0,2}$. Standard deviations were notably larger for the model with three components than for the model with two components.

- In conditions with heterogeneous effects ($\tau^2 = 0.36$),
  - the correct model (i.e., a model with two components and with effect heterogeneity) usually performed well in terms of bias. In some conditions with small numbers of studies, the log-linear model yielded a slight negative mean bias (compare subfigures F and G of Figure S2).
  - models without effect heterogeneity sometimes yielded a pronounced and systematic bias (as can be seen in subfigures F, G and H of Figures S2, S3, S5 and S6).

- the log-linear model with effect heterogeneity and three components sometimes had a notable mean bias in particular for small numbers of studies (see for example subfigures F and H of Figure S2). However, its median bias was negligible.
- results with regard to SD($\hat{\hat{\beta}}$) were similar to the results which were described for conditions with homogeneous effects (see above).

Figures S9 to S14 illustrate the results with regard to the estimation of $\hat{\hat{\beta}}$ for conditions with $S = 3$. Figures S9-S11 depict mean bias, median bias and SD($\hat{\hat{\beta}}$) for the log-linear mixture models, respectively. Figures S12-S14 depict mean bias, median bias and SD($\hat{\hat{\beta}}$) for the logistic mixture models, respectively. Again, results of log-linear and logistic mixture models were similar, and will thus be described simultaneously. These were the most important results:

- In conditions with homogeneous effects ($\tau^2 = 0$),
  - bias was negligible for all models investigated.
  - SD($\hat{\beta}$) was small for all models without effect heterogeneity across simulation conditions, and also for models with effect heterogeneity in conditions with $n_0 = 500$.
  - SD($\hat{\beta}$) was larger for models with effect heterogeneity in conditions with $n_0 = 50$, in particular for the log-linear model, and increased for models with a larger number of components and simulation conditions with smaller numbers of studies.
- In conditions with heterogeneous effects ($\tau^2 = 0.36$),
  - models with effect heterogeneity usually yielded a bias close to zero, in particular for conditions with larger numbers of studies. In one condition, the model with $S = 2$ and with effect heterogeneity yielded a systematic mean and median bias (see subfigure L of Figures S9 and S10). However, the correct model always performed well in terms of both mean and median bias.
  - models without effect heterogeneity often showed a systematic bias which did not decline for larger numbers of studies.
  - the findings with regard to SD($\hat{\beta}$) were similar as for conditions with homogeneous effects.

S2.5.3. Estimation of τ^2

Figures S15 to S17 show the results with regard to mean bias of $\hat{\tau}^2$, median bias of $\hat{\tau}^2$, and SD($\hat{\tau}^2$) for conditions with $S = 2$. Results are displayed separately for log-linear mixture models (red lines) and logistic mixture models (blue lines) with two components (solid lines)

and three components (dashed lines). Note that all models depicted in these figures are models with heterogeneous effects, since $\hat{\tau}^2 = 0$ for all models with homogeneous effects. The main results are the following:

- In conditions with large sample sizes ($n_0 = 500$),
  - bias of $\hat{\tau}^2$ was negligible for all models.
  - SD($\hat{\tau}^2$) was small for all models.
- In conditions with small sample sizes ($n_0 = 50$),
  - both mean and median bias were usually small for models with two components. In conditions in which the components were less separted in terms of the difference between $p_{0,1}$ and $p_{0,2}$, there was sometimes a small positive bias for small numbers of studies, in particular when effects were truly homogeneous.
  - larger biases were typically observed for models with three components, and in particular for the log-linear model. Generally, bias was larger for smaller numbers of studies.
  - SD($\hat{\tau}^2$) was often notably larger than in conditions with large sample sizes. This affected in particular those conditions in which components were less separated in terms of the difference between $p_{0,1}$ and $p_{0,2}$ and conditions with small numbers of studies. SD($\hat{\tau}^2$) was larger for models with three components than for models with two components, and was particularly large for the log-linear models.

Figures S18 to S20 show the results with regard to mean bias of $\hat{\tau}^2$, median bias of $\hat{\tau}^2$, and SD($\hat{\tau}^2$) for conditions with $S = 3$. Results are displayed separately for log-linear mixture models (red lines) and logistic mixture models (blue lines) with two components (solid lines) and three components (dashed lines). These are the main results:

- In conditions with large sample sizes ($n_0 = 500$),
  - bias of $\hat{\tau}^2$ was negligible for all models in conditions with homogeneous effects.
  - models with $S = 2$ sometimes yielded a pronounced and systematic negative bias in conditions with heterogeneous effects, irrespective of the number of studies.
  - SD($\hat{\tau}^2$) was small for all models.
- In conditions with small sample sizes ($n_0 = 50$),
  - models with larger numbers of components (i.e., $S = 3$ and $S = 4$) - in particular the log-linear mixture models - often yielded a pronounced mean bias in conditions with

smaller numbers of studies. Median bias of these models was notably smaller than mean bias, which raises the suspicion that mean bias was affected by outliers.

- large values of SD($\hat{\tau}^2$) were obtained for models with $S = 3$ and $S = 4$, in particular in conditions with smaller numbers of studies. Particularly large standard deviations were obtained from the log-linear mixture models, and similar to mean bias, these might have been affected by outliers.

S2.6. Estimation of β_s

S2.6 Estimation of β_s

Figure S21 displays histograms of $\hat{\beta}_1$ and $\hat{\beta}_2$ estimated by a log-linear mixture model with two components and with effect heterogeneity for a selection of simulation conditions with two components. The parameter configuration of the respective simulation condition is denoted in the titles of the subfigures. Note that since the component order is not well-defined in nonparametric mixture models, the mapping of component estimates to $\hat{\beta}_1$ and $\hat{\beta}_2$ was obtained by ordering the estimates and then denoting the smallest estimate $\hat{\beta}_1$ and the largest estimate $\hat{\beta}_2$. The figures show that for large sample sizes within studies, the component effects were estimated with a mean bias of almost zero and small variances. For smaller sample sizes, the variances were notably larger and estimates sometimes had a large mean bias, in particular when components were less well separated in terms of the difference between $p_{0,1}$ $p_{0,2}$. Similar results were obtained for conditions with different numbers of studies, different orders of $(p_{0,1}, p_{0,2})$ and for different values of q_1 as well as for the logistic mixture model.

Figure S22 shows histograms of $\hat{\beta}_1$, $\hat{\beta}_2$ and $\hat{\beta}_3$ estimated by a log-linear model with three components and with effect heterogeneity for a selection of simulation conditions with three components. The parameter configuration of the respective simulation condition is denoted in the titles of the subfigures. The mapping of component estimates to $\hat{\beta}_1$, $\hat{\beta}_2$ and $\hat{\beta}_3$ was obtained in an analogous way as for the results from the simulation with $S = 2$. Similar to the results for the simulation with $S = 2$, we see that component estimates were almost unbiased and had small variances for large sample sizes within studies. In contrast, variances were much larger for conditions with small sample sizes and estimates often had a notable mean bias. Similar results were obtained for conditions with different numbers of studies and different orders of $(p_{0,1}, p_{0,2}, p_{0,3})$ as well as for the logistic mixture model.

S2.7. Conclusion

S2.7 Conclusion

The following limitations of our simulation study must be considered:

- We only considered simulation conditions with a relatively large number of studies.

Meta-analyses of rare events from the *Cochrane Database of Systematic Reviews* are typically based on a smaller number of studies. Note that our results might not be generalisable to smaller numbers of studies.

- We only considered simulation conditions with two or three components. Results might not be generalizable to larger numbers of components.
- We did not consider alternative models, such as generalized linear mixed models, as competitors in our simulation.
- We did not investigate simulation scenarios in which all models were misspecified.

We formulate the following conclusions of our simulation study:

- For both log-linear and logistic mixture models, a good model selection performance can be achieved with both the AIC and the BIC as long a (i) sample sizes within studies are large or (ii) components are well separated in terms of their baseline event probabilities. If none of this is the case, a good selection performance might still be achieved if the number of studies is very large, at least when there are only two underlying components. When it is expected that there are more than two components, we strongly recommend to use the AIC and BIC for model selection only if sample sizes within studies are fairly large. By "good selection performance", we mean that the correct model was selected in a large percentage of simulation replications among (i) models which are misspecified in terms of the number of components, (ii) models which are misspecified in terms of their assumption regarding effect heterogeneity and (iii) models which are misspecified in both ways.
- Regarding the estimation of a pooled effect ($\bar{\beta}$), both log-linear and logistic mixture models yield almost unbiased estimates with small standard deviations when there are two underlying components and (i) effects are truly homogeneous or (ii) the correct model is selected. For three components, we obtained similar results, but note that larger numbers of studies or large sample sizes within studies might be required to achieve almost unbiased estimates when effects are truly heterogeneous.
- Regarding the estimation of heterogeneity (τ^2), almost unbiased estimates with small standard deviations can be obtained from log-linear and logistic mixture models as long as the correct model is selected and either sample sizes within studies are large or the number of studies is large.
- As long as sample sizes within studies are large, one can expect to obtain estimates of component effect sizes which are almost unbiased.

References

Beisemann, M., Doebler, P. and Holling, H. (2020). Comparison of random-effects meta-analysis models for the relative risk in the case of rare events: A simulation study. *Biometrical Journal, 62*(7). doi:10.1002/bimj.201900379

Grün, B. and Leisch, F. (2008). FlexMix Version 2: Finite mixtures with concomitant variables and varying and constant parameters *Journal of Statistical Software, 28*(4), 1-35. doi:10.18637/jss.v028.i04

Microsoft Corporation and Weston, S. (2020). doParallel: Foreach Parallel Adaptor for the 'parallel' Package. R package version 1.0.16. https://CRAN.R-project.org/package=doParallel

Moher, D., Tetzlaff, J., Tricco, A. C., Sampson, M. and Altman, D. G. (2007). Epidemiology and Reporting Characteristics of Systematic Reviews. *Plos Medicine, 4*(3), e78. doi:10.1371/journal.pmed.0040078

R Core Team (2020). R: A language and environment for statistical computing. R Foundation for Statistical Computing, Vienna, Austria. URL https://www.R-project.org/.

Turner, R. M., Davey, J., Clarke, M. J., Thompson, S. G. and Higgins, J. P. T. (2012). Predicting the extent of heterogeneity in meta-analysis, using empirical data from the Cochrane Database of Systematic Reviews. *International Journal of Epidemiology, 41*, 818-827. doi:10.1093/ije/dys041

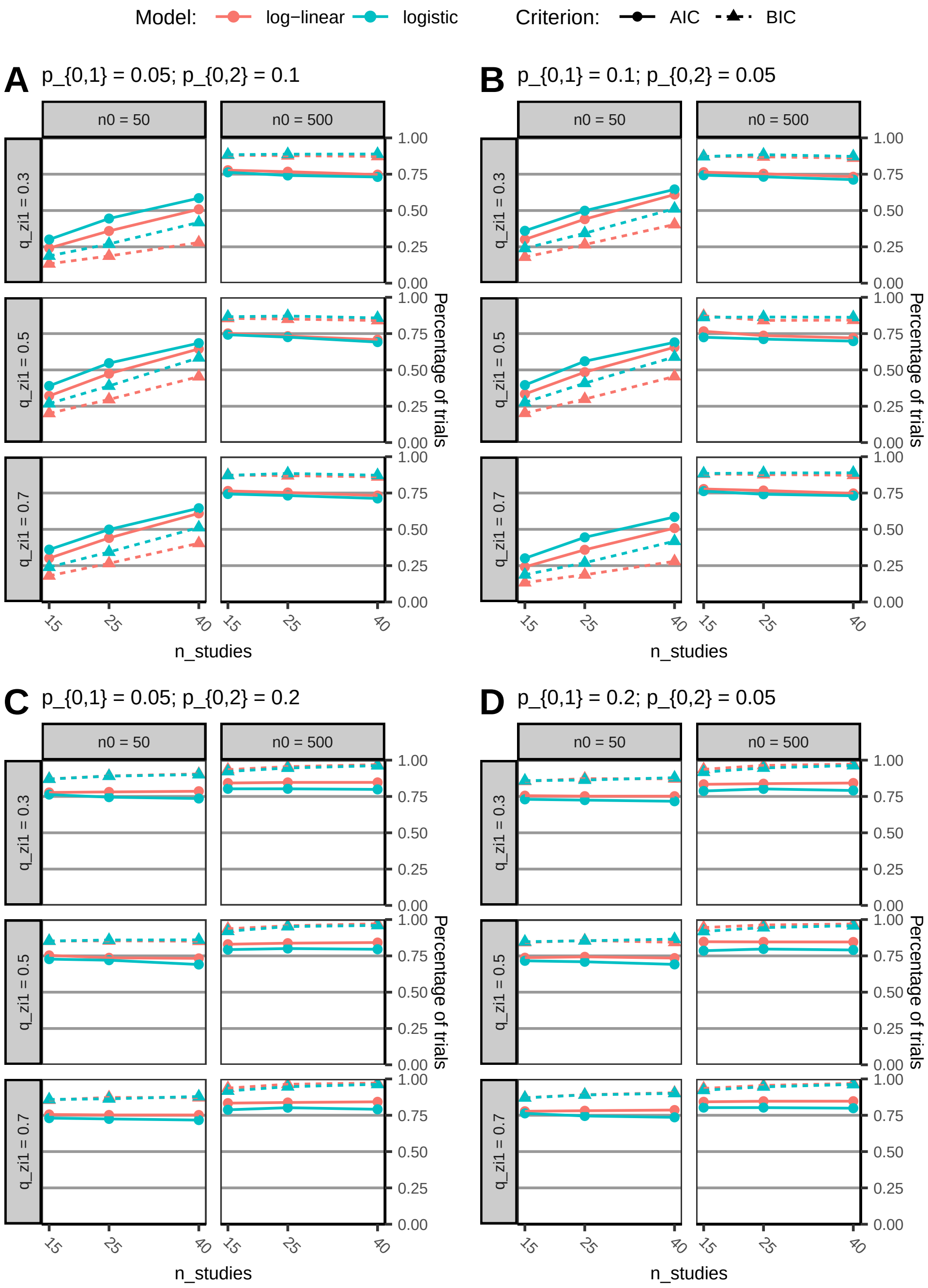

Figure S1.

Percentage of simulation replications in which the correct model was favoured by the AIC (solid line) or BIC (dashed line) for conditions with $S = 2$. Results for the log-linear model are depicted in red, results for the logistic model are depicted in blue. A-D: Simulation conditions with $\tau^2 = 0$ (homogeneous effects), E-H: Simulation conditions with $\tau^2 = 0.36$ (heterogeneous effects).

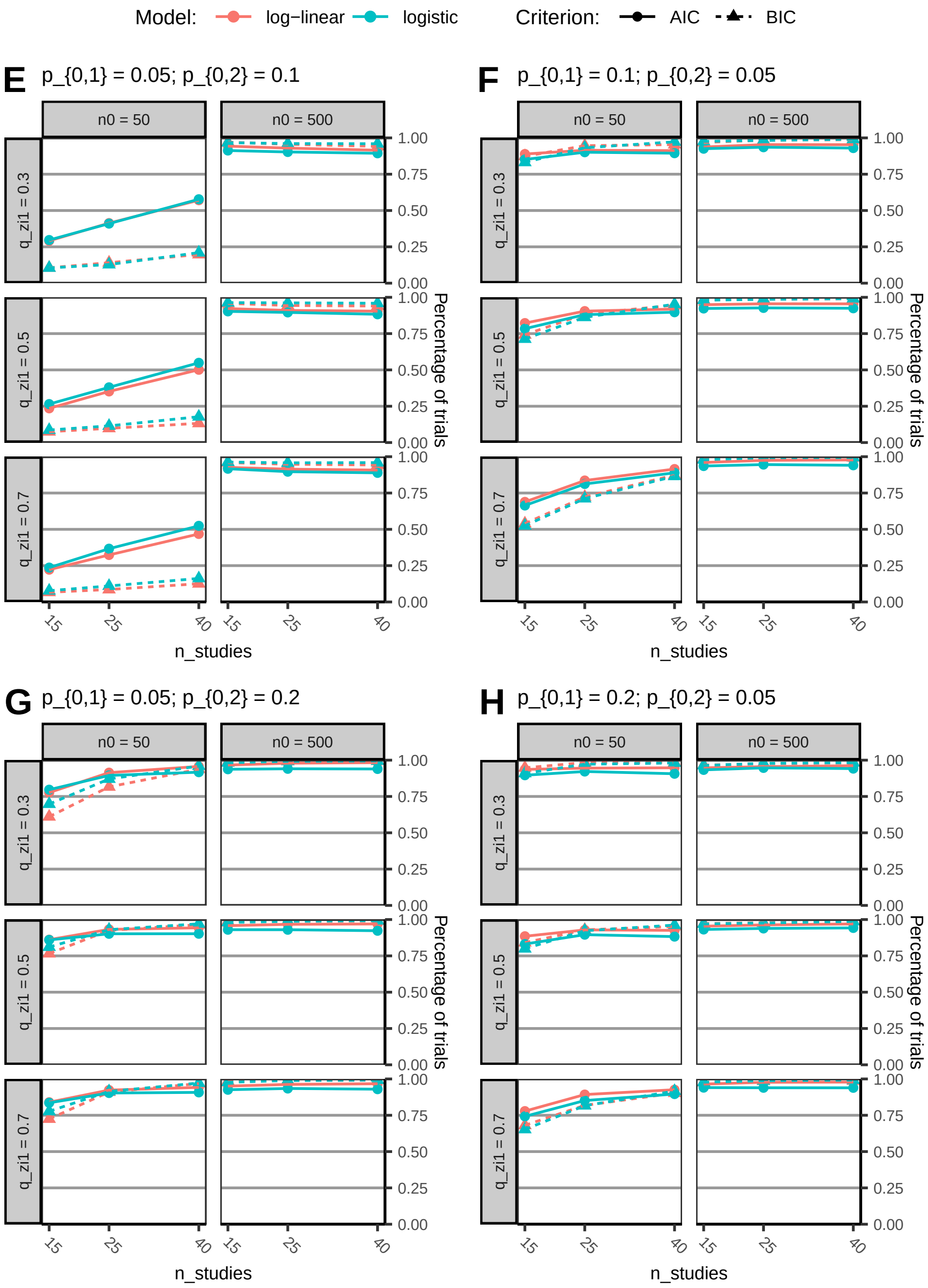

Figure S1.
(continued) Percentage of simulation replications in which the correct model was favoured by the AIC (solid line) or BIC (dashed line) for conditions with $S = 2$. Results for the log-linear model are depicted in red, results for the logistic model are depicted in blue. A-D: Simulation conditions with $\tau^2 = 0$ (homogeneous effects), E-H: Simulation conditions with $\tau^2 = 0.36$ (heterogeneous effects).

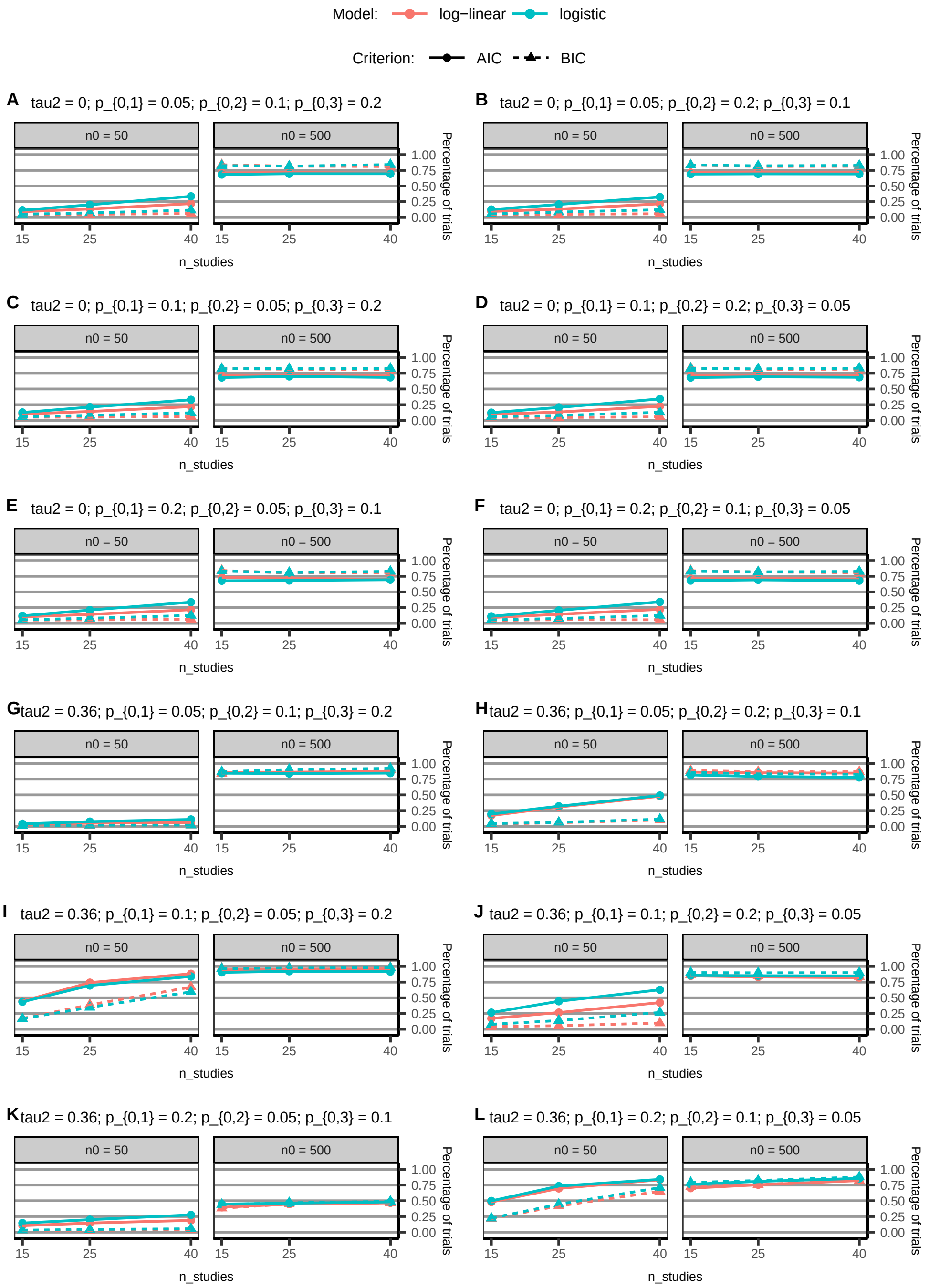

FIGURE S2.
Percentage of simulation replications in which the correct model was favoured by the AIC (solid line) or BIC (dashed line) for conditions with $S = 3$. Results for the log-linear model are depicted in red, results for the logistic model are depicted in blue. A-F: Simulation conditions with $\tau^2 = 0$ (homogeneous effects), G-L: Simulation conditions with $\tau^2 = 0.36$ (heterogeneous effects).

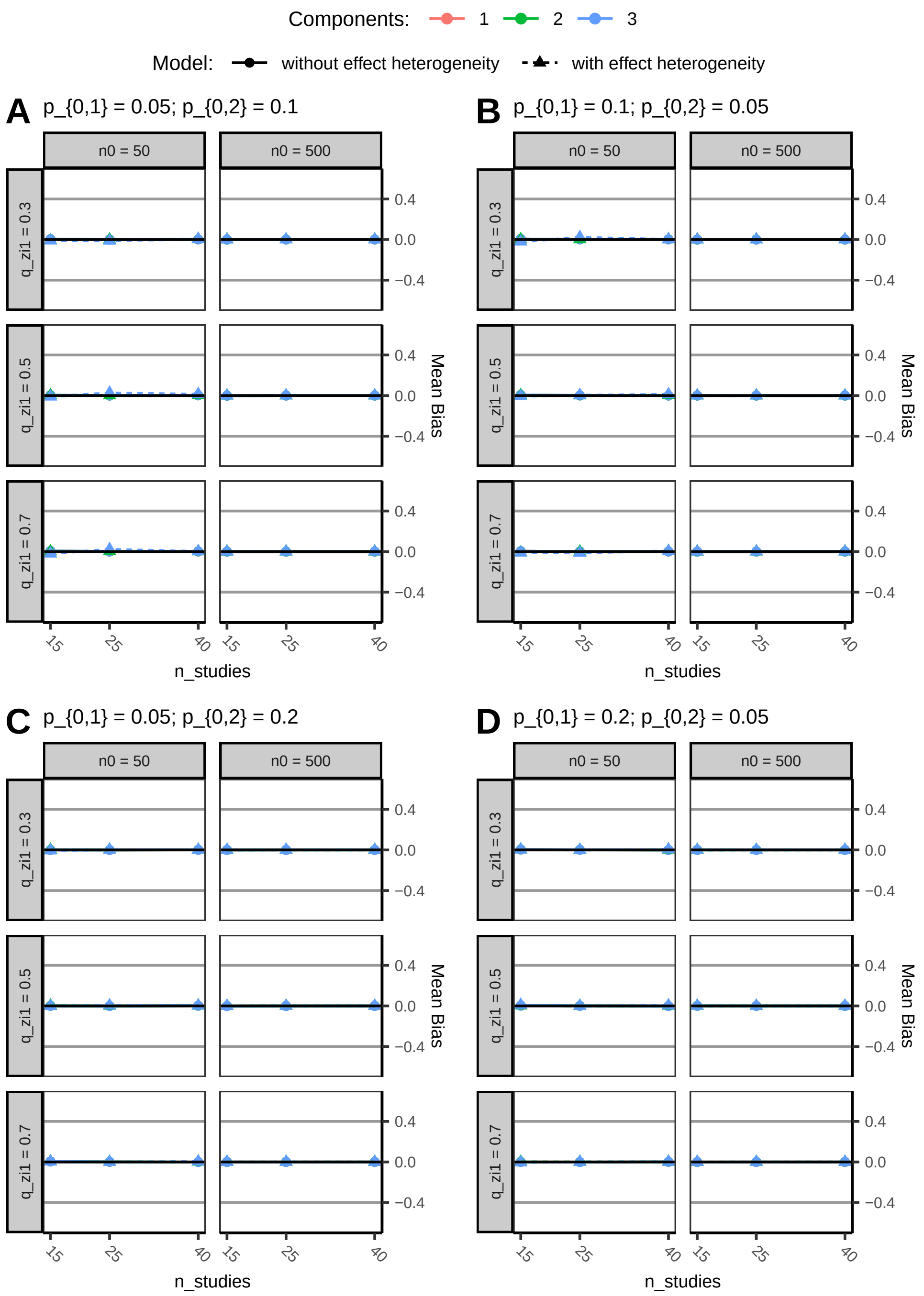

FIGURE S3.
Mean Bias of $\hat{\hat{\beta}}$ for each log-linear mixture model for conditions with $S = 2$. The number of components of the respective model is colour-coded. Models with identical slopes are depicted with solid lines. Models with varying slope are depicted with dashed lines. A-D: Simulation conditions with $\tau^2 = 0$ (homogeneous effects), E-H: Simulation conditions with $\tau^2 = 0.36$ (heterogeneous effects).

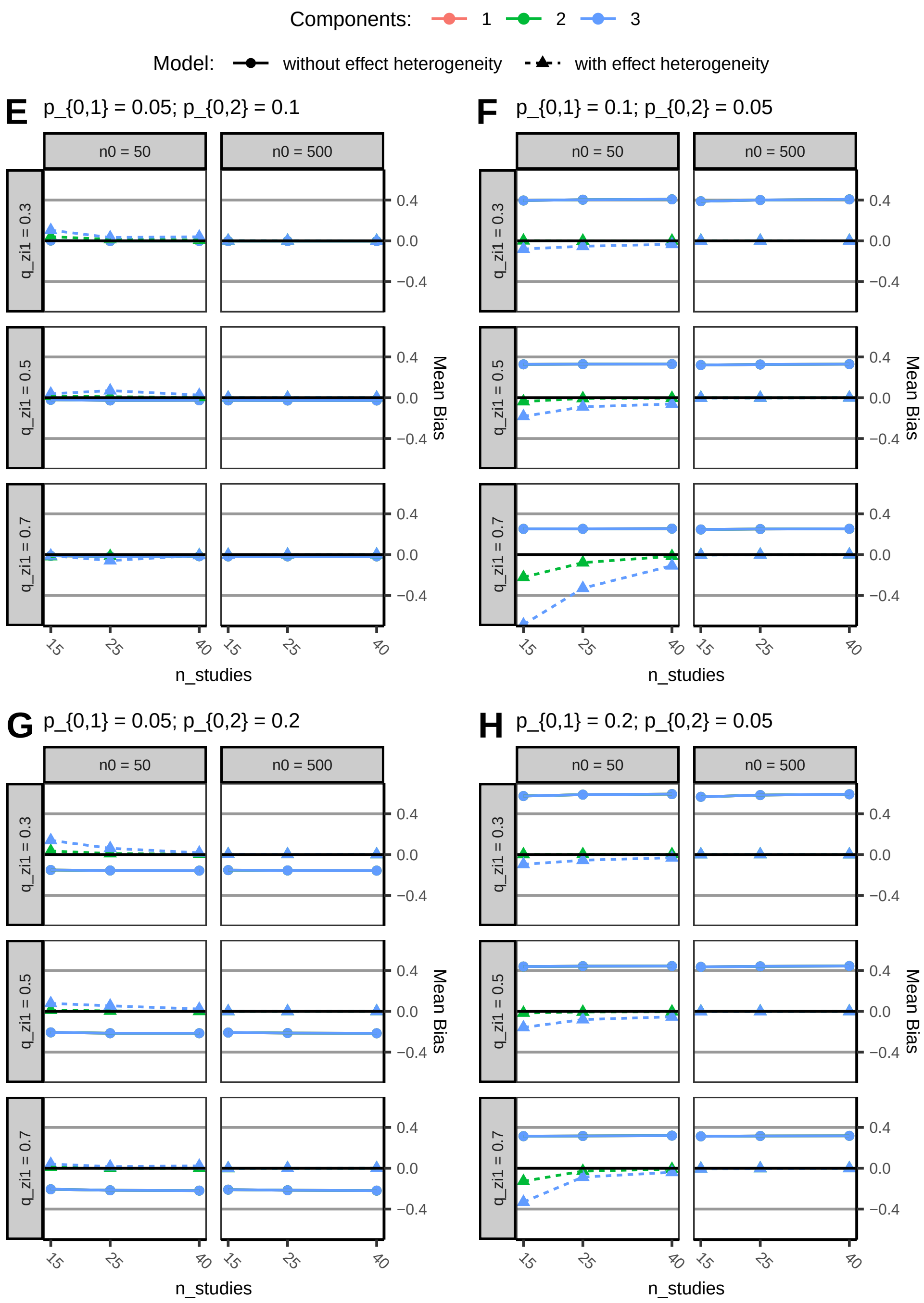

FIGURE S3.
(continued) Mean Bias of $\hat{\hat{\beta}}$ for each log-linear mixture model for conditions with $S = 2$. The number of components of the respective model is colour-coded. Models with identical slopes are depicted with solid lines. Models with varying slope are depicted with dashed lines. A-D: Simulation conditions with $\tau^2 = 0$ (homogeneous effects), E-H: Simulation conditions with $\tau^2 = 0.36$ (heterogeneous effects).

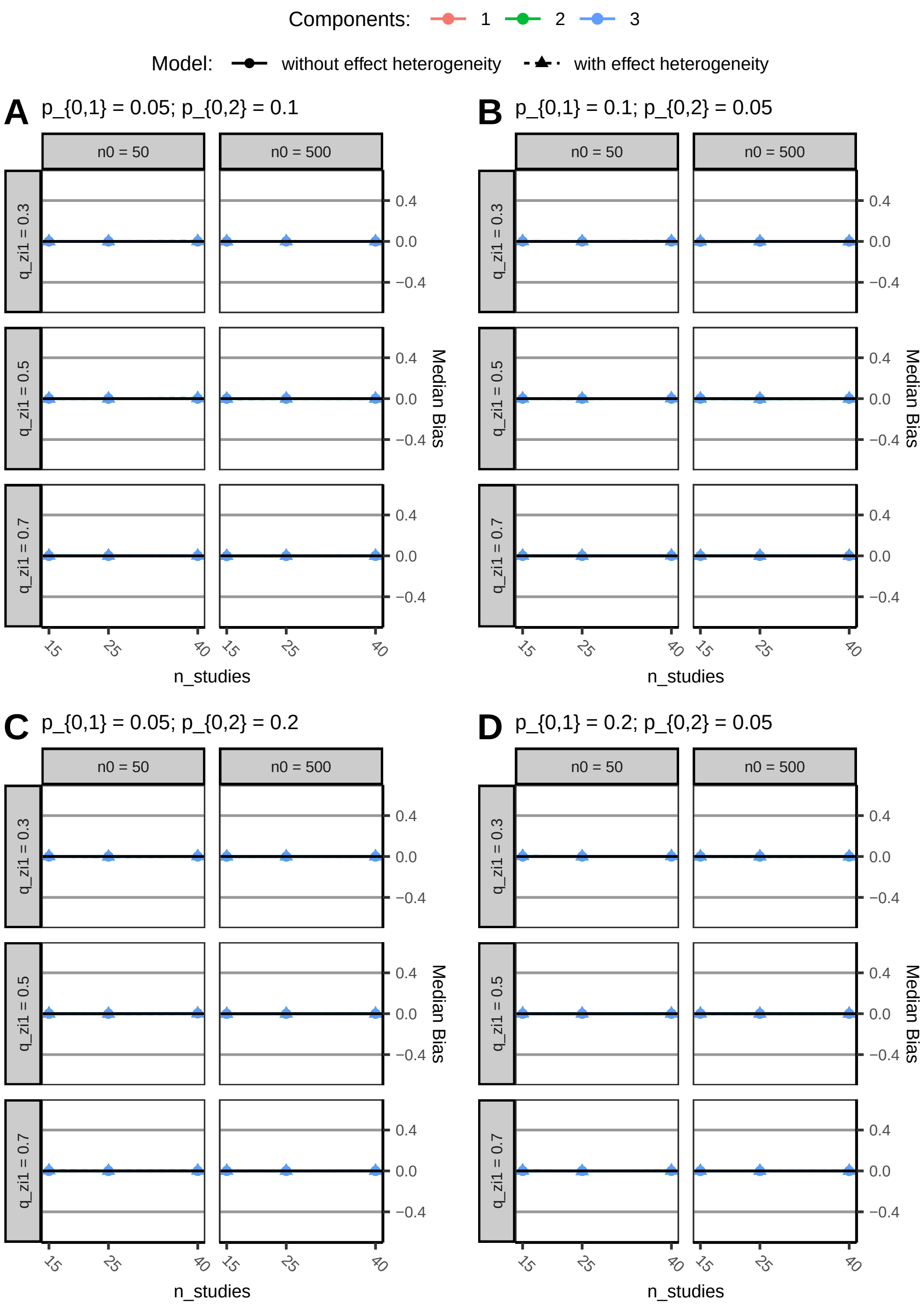

FIGURE S4.
Median Bias of $\hat{\hat{\beta}}$ for each log-linear mixture model for conditions with $S = 2$. The number of components of the respective model is colour-coded. Models with identical slopes are depicted with solid lines. Models with varying slope are depicted with dashed lines. A-D: Simulation conditions with $\tau^2 = 0$ (homogeneous effects), E-H: Simulation conditions with $\tau^2 = 0.36$ (heterogeneous effects).

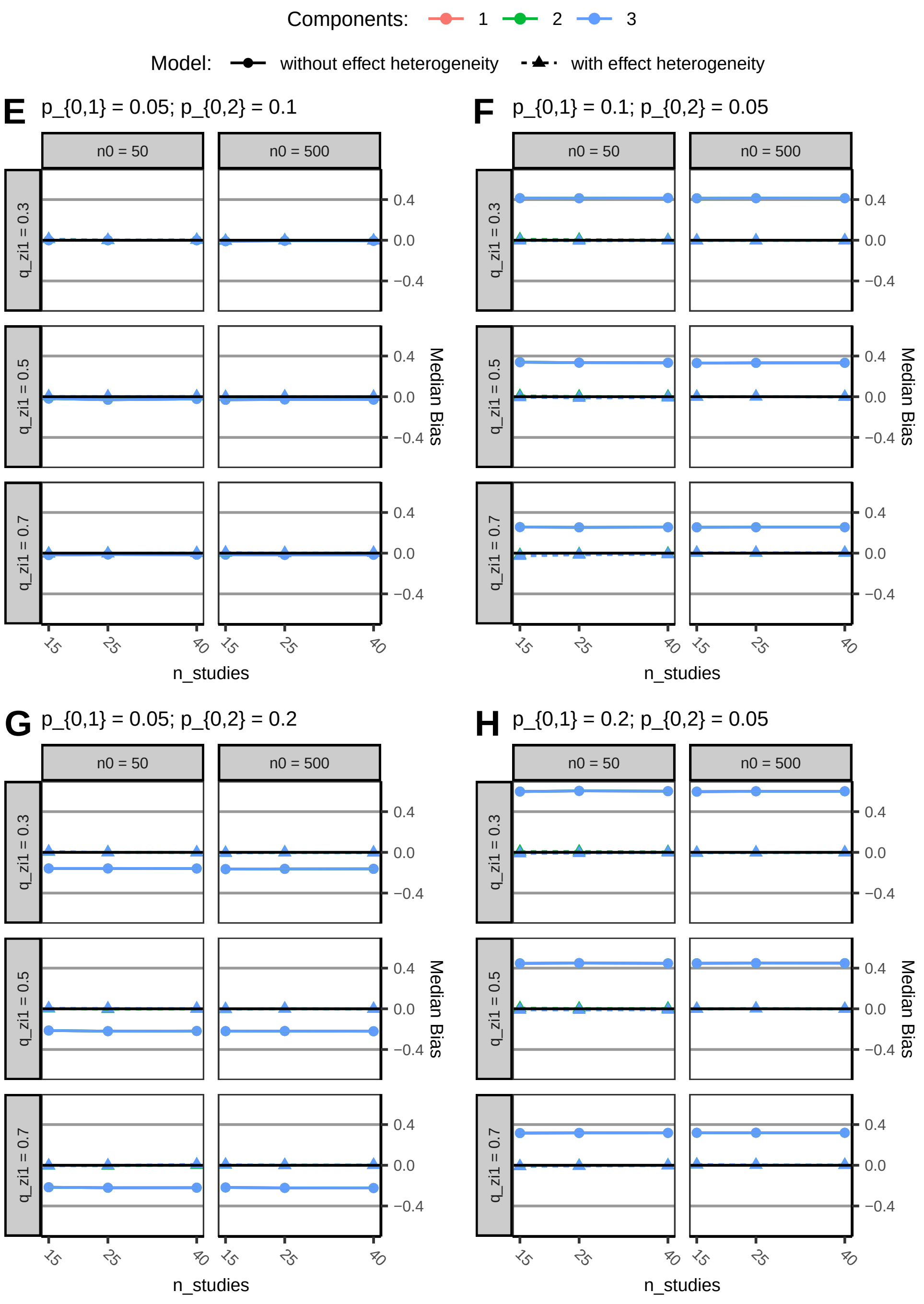

Figure S4.
(continued) Median Bias of $\hat{\bar{\beta}}$ for each log-linear mixture model for conditions with $S = 2$. The number of components of the respective model is colour-coded. Models with identical slopes are depicted with solid lines. Models with varying slope are depicted with dashed lines. A-D: Simulation conditions with $\tau^2 = 0$ (homogeneous effects), E-H: Simulation conditions with $\tau^2 = 0.36$ (heterogeneous effects).

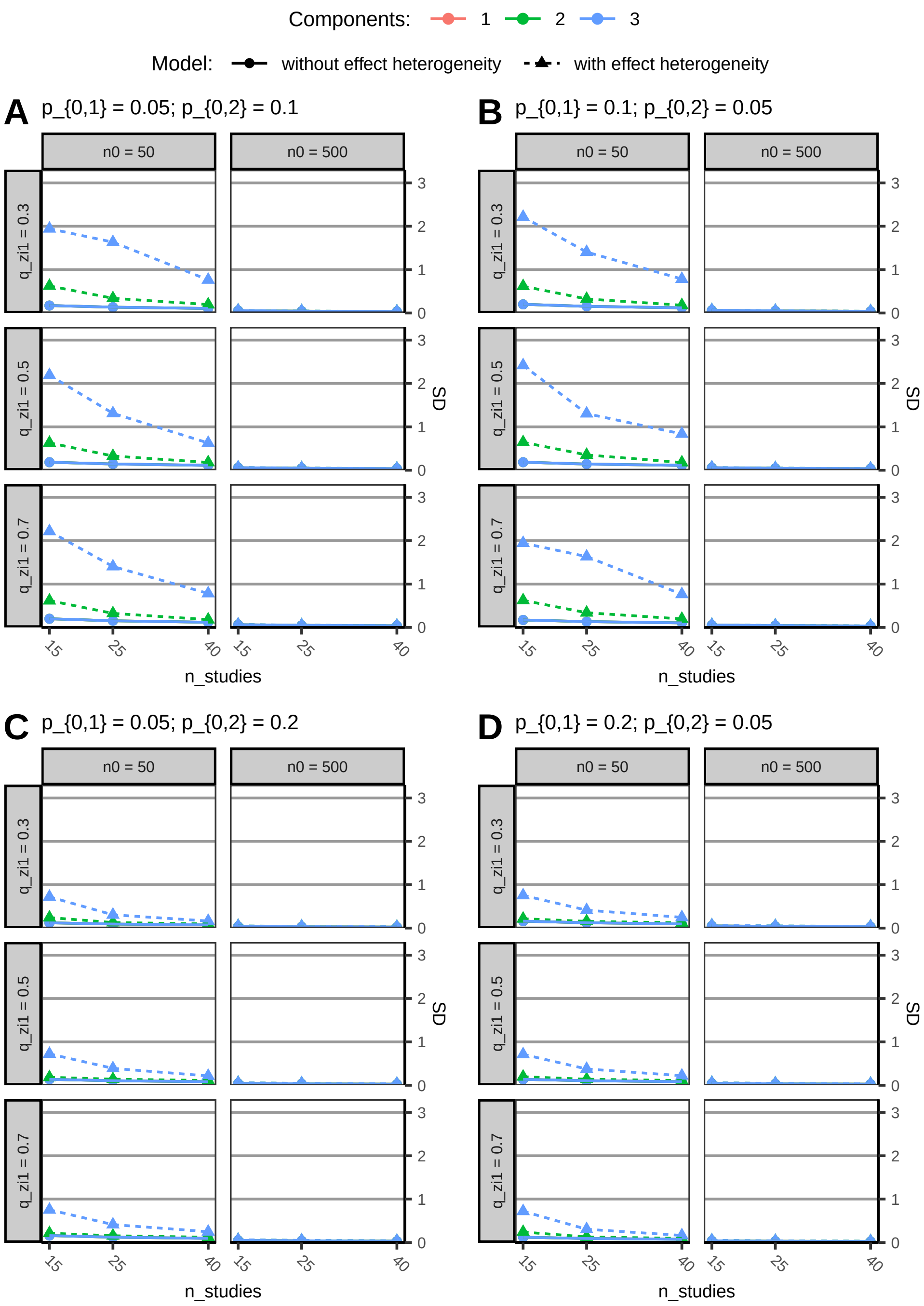

FIGURE S5.
Standard deviation of $\hat{\hat{\beta}}$ for each log-linear mixture model for conditions with $S = 2$. The number of components of the respective model is colour-coded. Models with identical slopes are depicted with solid lines. Models with varying slope are depicted with dashed lines. A-D: Simulation conditions with $\tau^2 = 0$ (homogeneous effects), E-H: Simulation conditions with $\tau^2 = 0.36$ (heterogeneous effects).

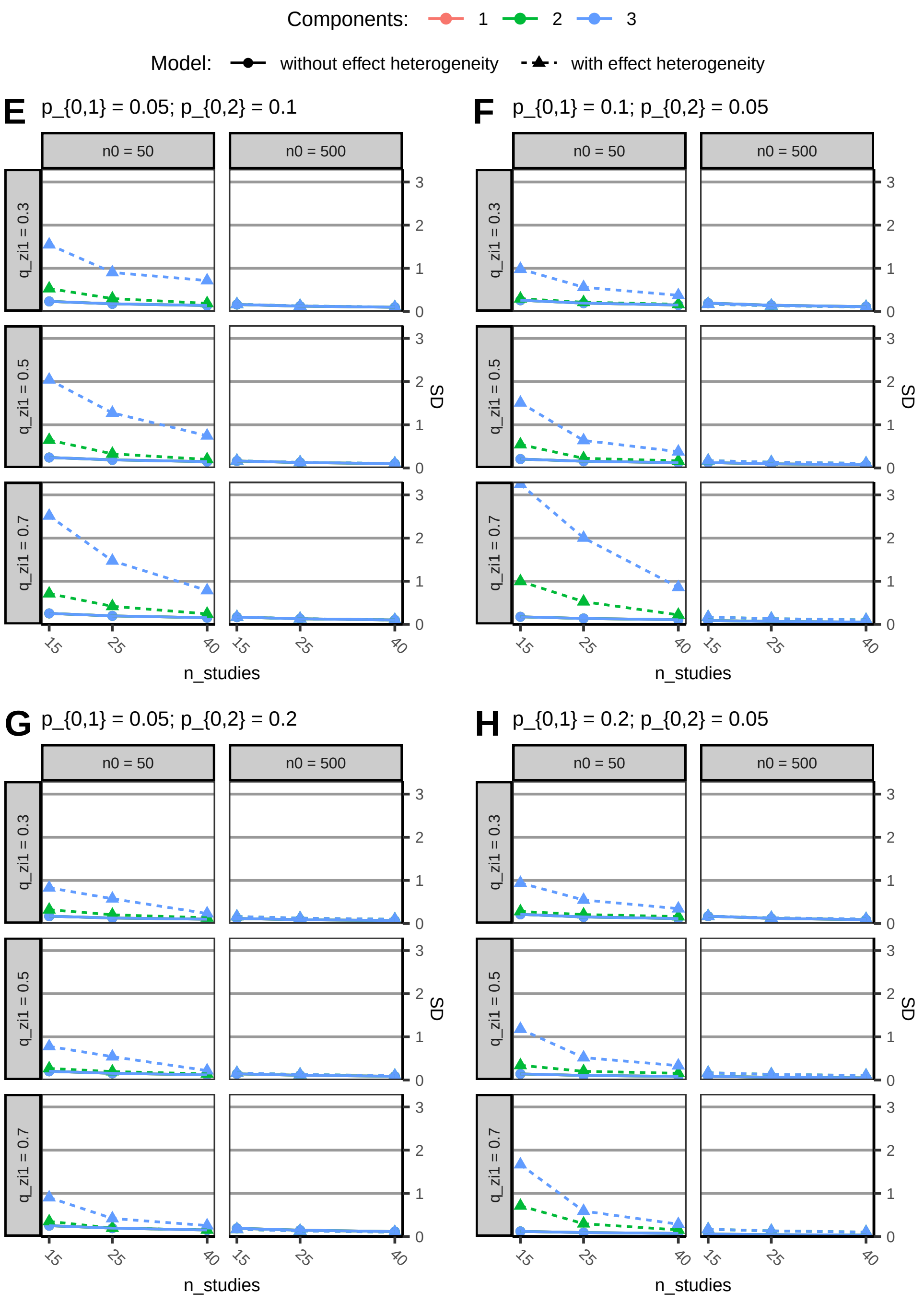

Figure S5.
(continued) Standard deviation of $\hat{\hat{\beta}}$ for each log-linear mixture model for conditions with $S = 2$. The number of components of the respective model is colour-coded. Models with identical slopes are depicted with solid lines. Models with varying slope are depicted with dashed lines. A-D: Simulation conditions with $\tau^2 = 0$ (homogeneous effects), E-H: Simulation conditions with $\tau^2 = 0.36$ (heterogeneous effects).

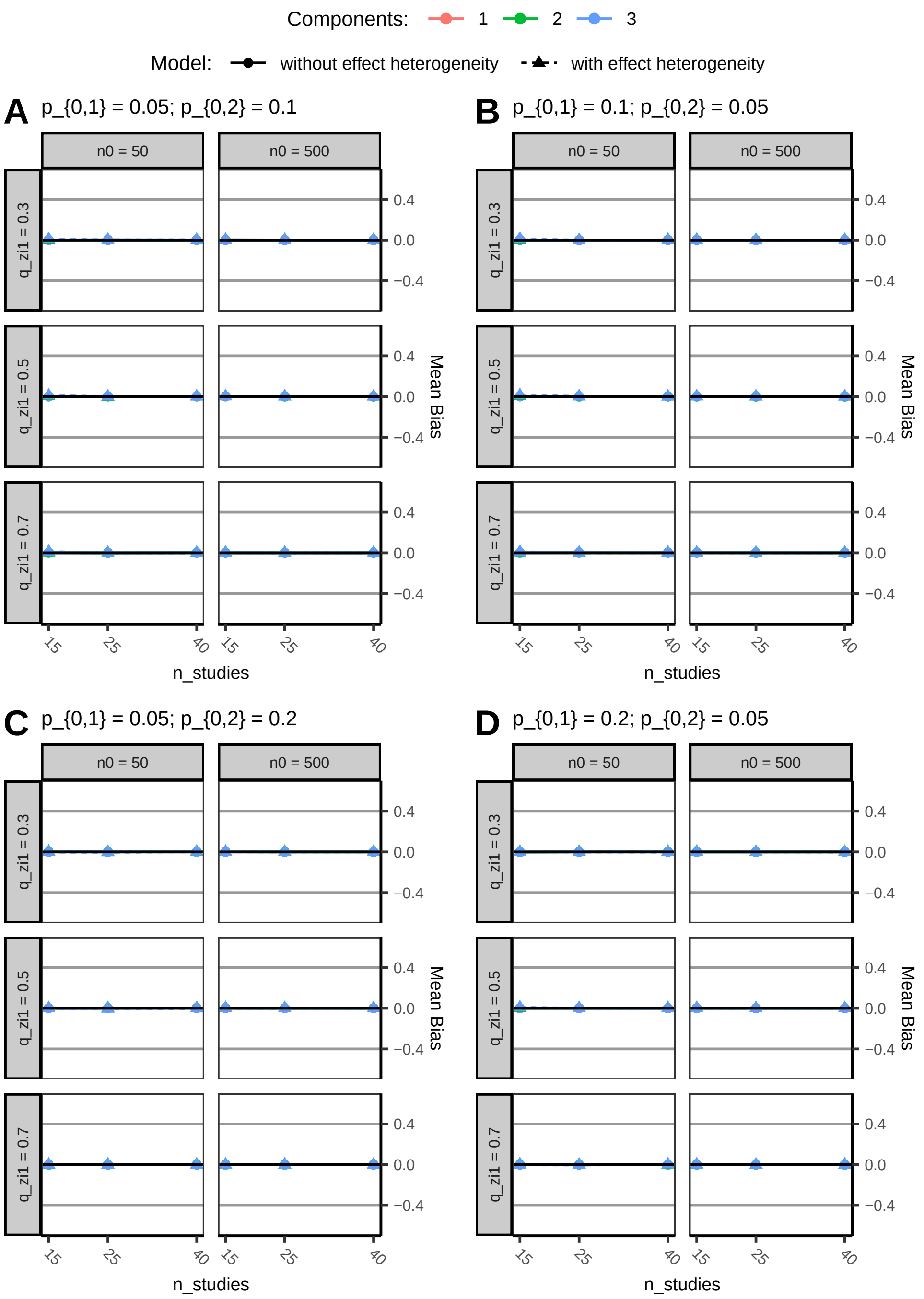

FIGURE S6.
Mean Bias of $\hat{\hat{\beta}}$ for each logistic mixture model for conditions with $S = 2$. The number of components of the respective model is colour-coded. Models with identical slopes are depicted with solid lines. Models with varying slope are depicted with dashed lines. A-D: Simulation conditions with $\tau^2 = 0$ (homogeneous effects), E-H: Simulation conditions with $\tau^2 = 0.36$ (heterogeneous effects).

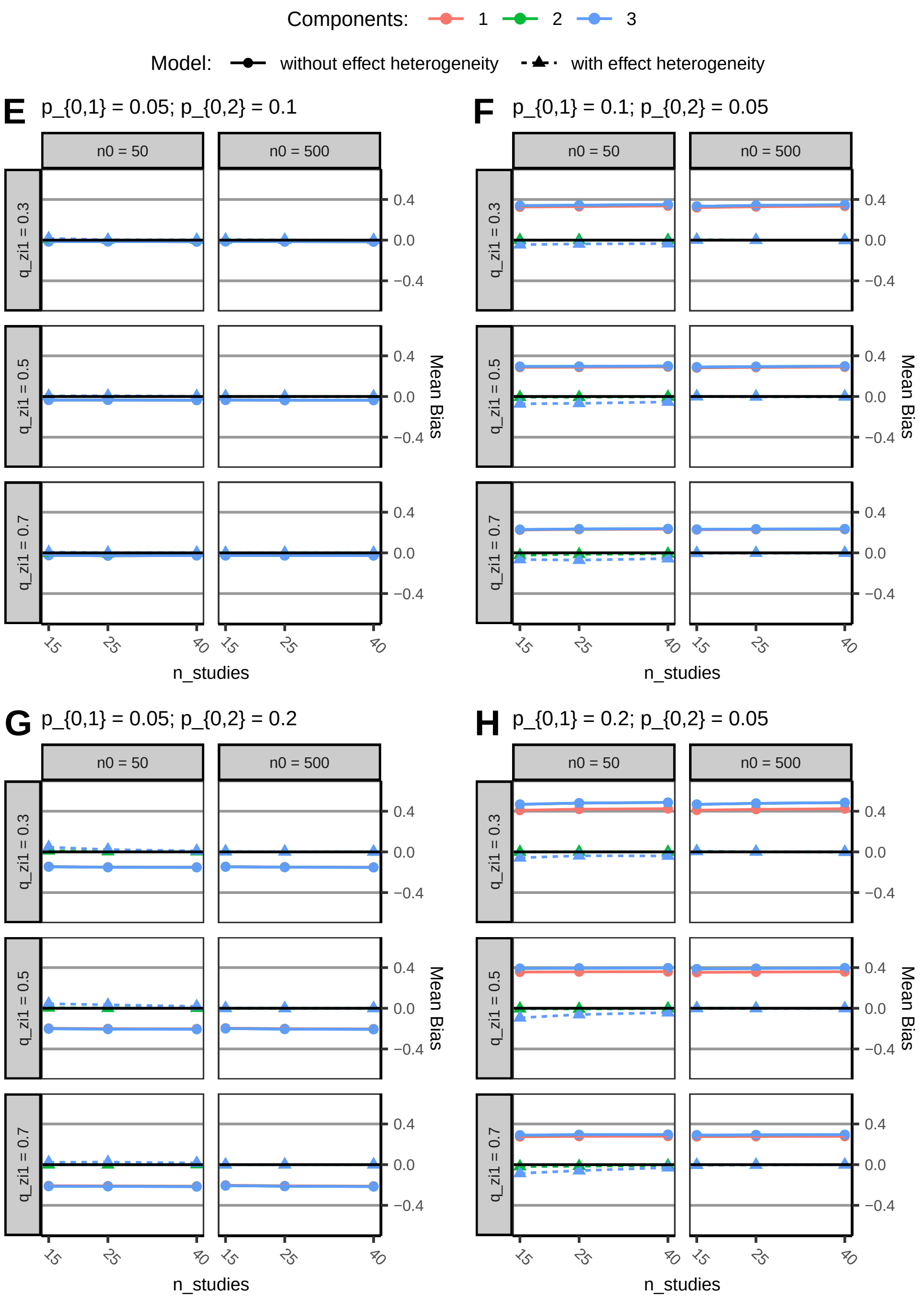

FIGURE S6.
(continued) Mean Bias of $\hat{\hat{\beta}}$ for each logistic mixture model for conditions with $S = 2$. The number of components of the respective model is colour-coded. Models with identical slopes are depicted with solid lines. Models with varying slope are depicted with dashed lines. A-D: Simulation conditions with $\tau^2 = 0$ (homogeneous effects), E-H: Simulation conditions with $\tau^2 = 0.36$ (heterogeneous effects).

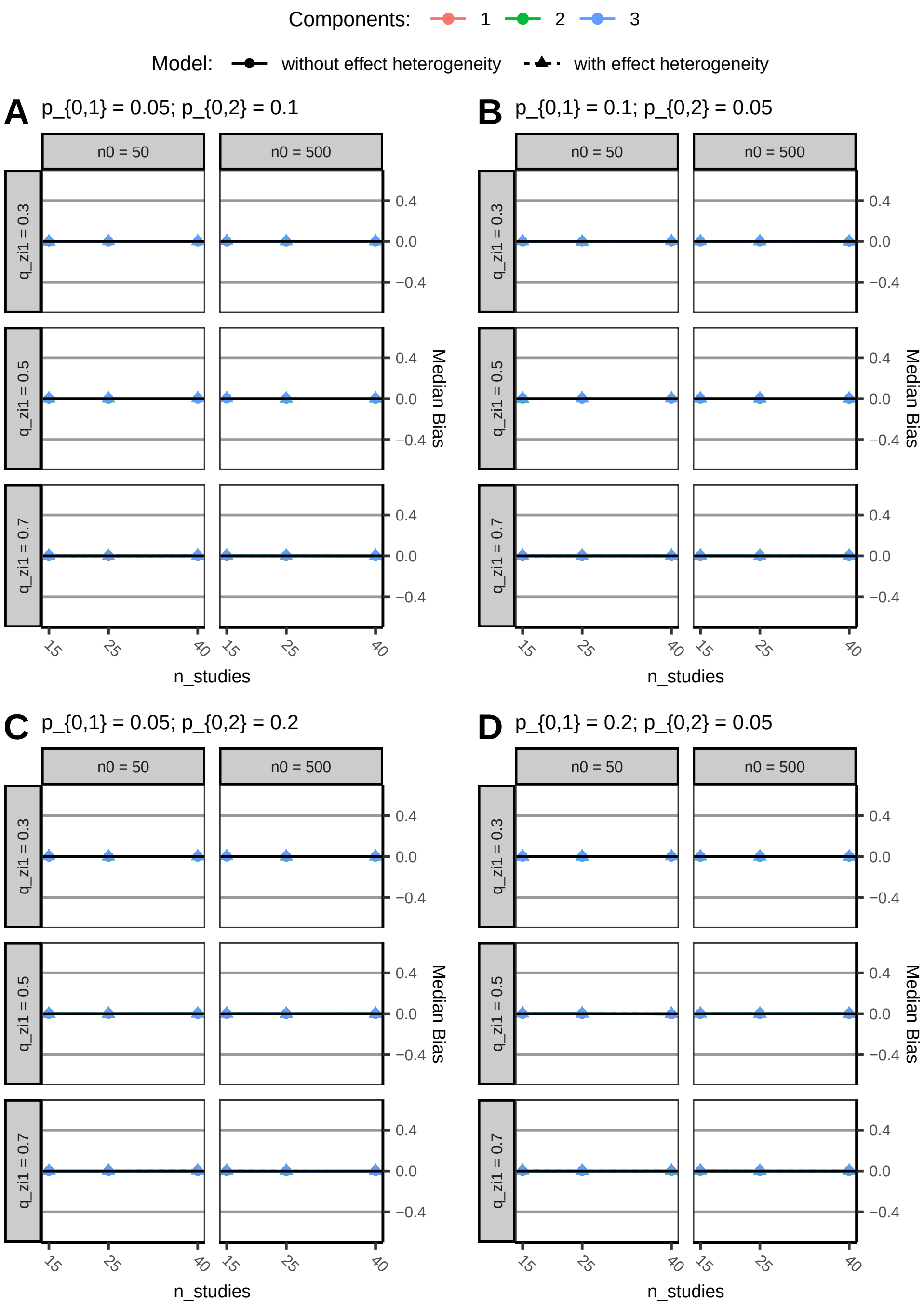

FIGURE S7.
Median Bias of $\hat{\hat{\beta}}$ for each logistic mixture model for conditions with $S = 2$. The number of components of the respective model is colour-coded. Models with identical slopes are depicted with solid lines. Models with varying slope are depicted with dashed lines. A-D: Simulation conditions with $\tau^2 = 0$ (homogeneous effects), E-H: Simulation conditions with $\tau^2 = 0.36$ (heterogeneous effects).

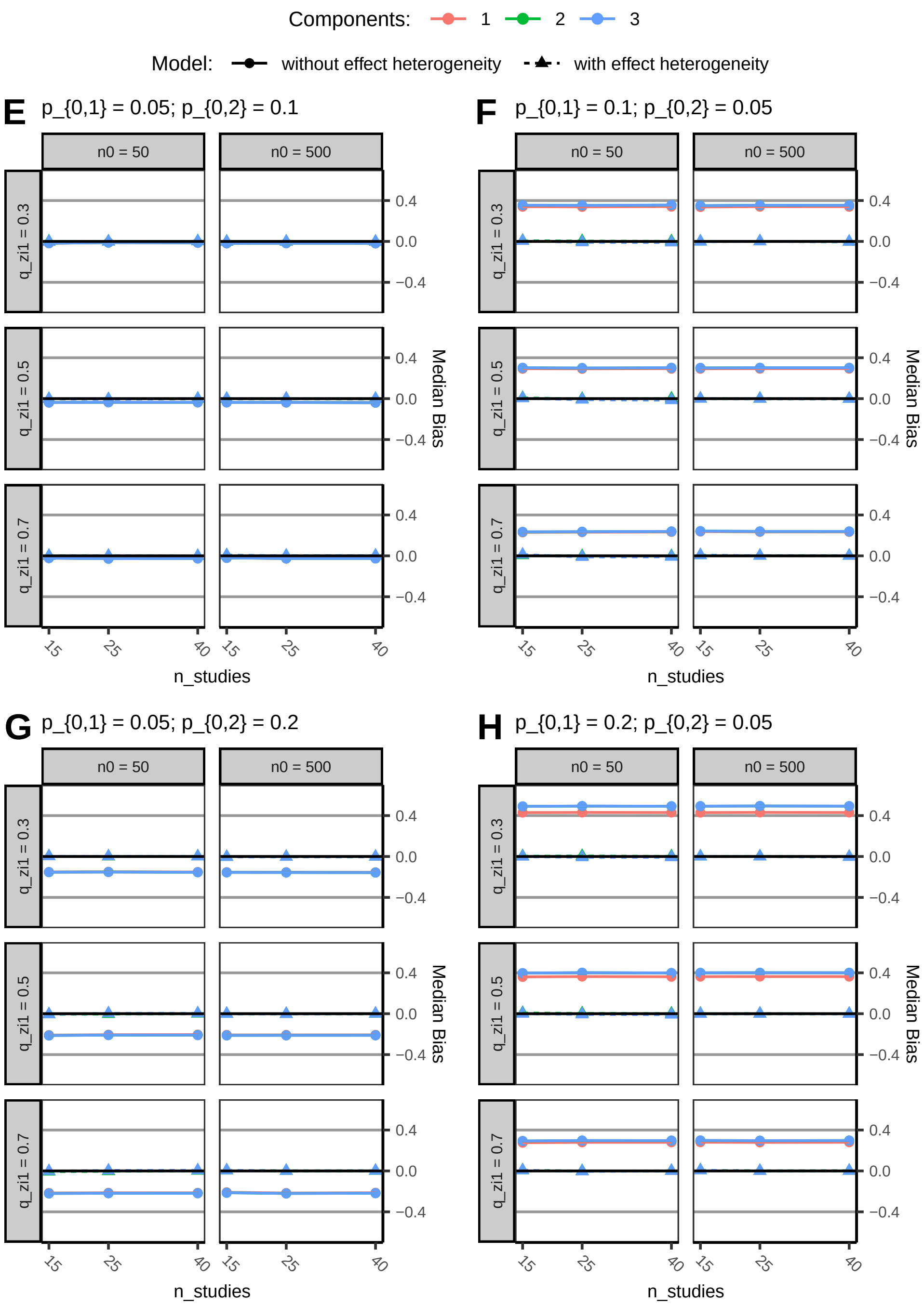

FIGURE S7.

(continued) Median Bias of $\hat{\hat{\beta}}$ for each logistic mixture model for conditions with $S = 2$. The number of components of the respective model is colour-coded. Models with identical slopes are depicted with solid lines. Models with varying slope are depicted with dashed lines. A-D: Simulation conditions with $\tau^2 = 0$ (homogeneous effects), E-H: Simulation conditions with $\tau^2 = 0.36$ (heterogeneous effects).

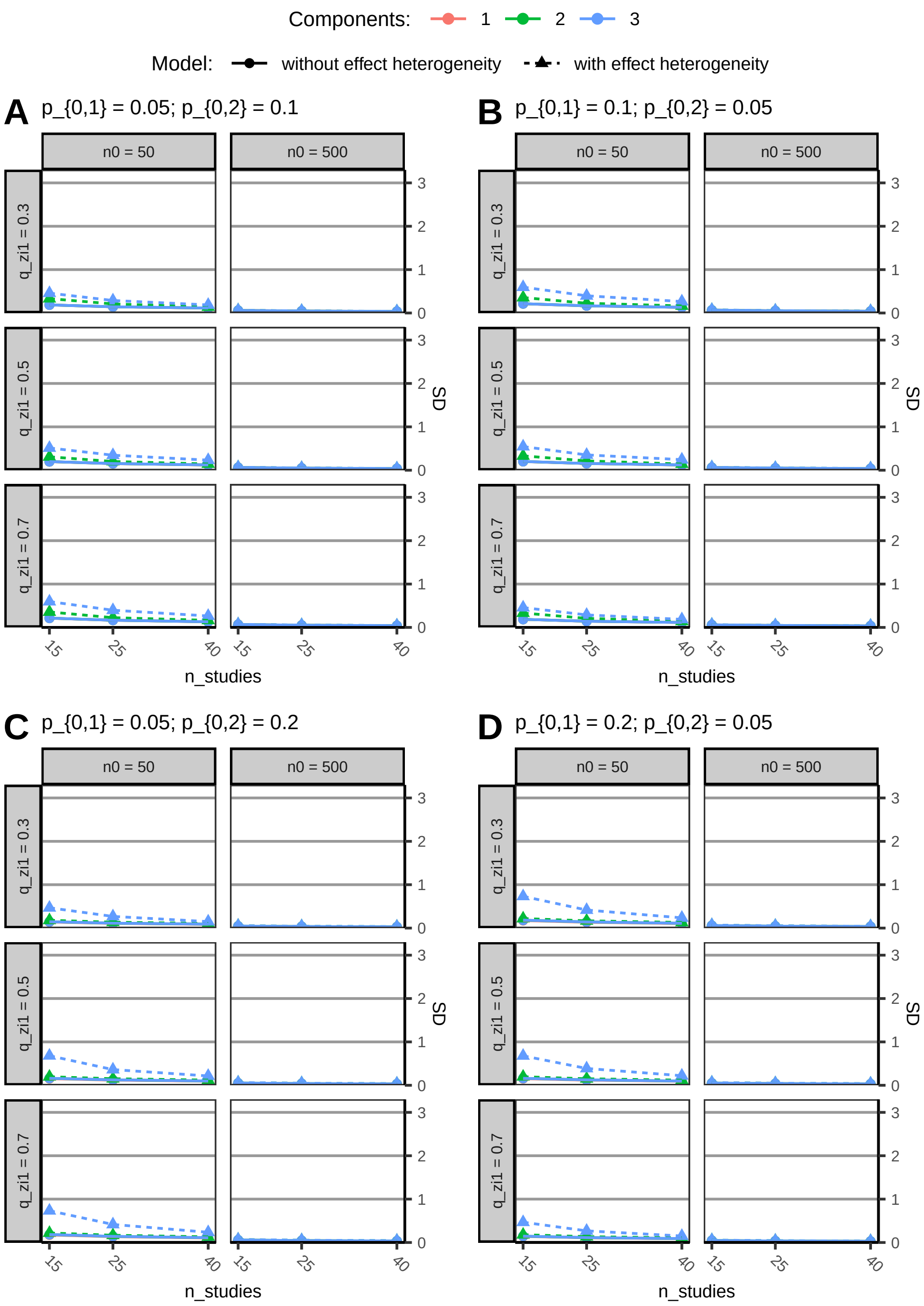

FIGURE S8.
Standard deviation of $\hat{\hat{\beta}}$ for each logistic mixture model for conditions with $S = 2$. The number of components of the respective model is colour-coded. Models with identical slopes are depicted with solid lines. Models with varying slope are depicted with dashed lines. A-D: Simulation conditions with $\tau^2 = 0$ (homogeneous effects), E-H: Simulation conditions with $\tau^2 = 0.36$ (heterogeneous effects).

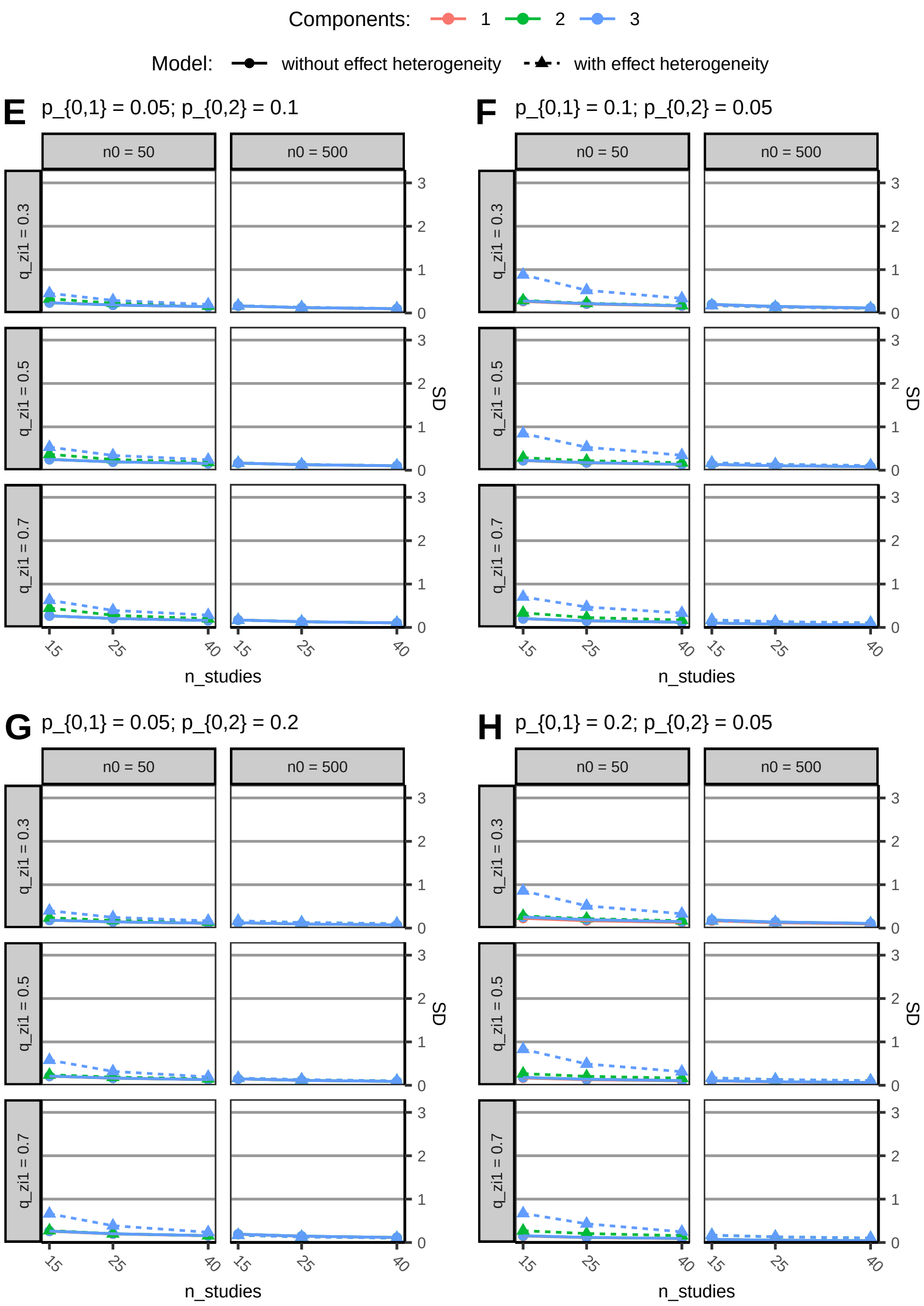

FIGURE S8.
(continued) Standard deviation of $\hat{\hat{\beta}}$ for each logistic mixture model for conditions with $S = 2$. The number of components of the respective model is colour-coded. Models with identical slopes are depicted with solid lines. Models with varying slope are depicted with dashed lines. A-D: Simulation conditions with $\tau^2 = 0$ (homogeneous effects), E-H: Simulation conditions with $\tau^2 = 0.36$ (heterogeneous effects).

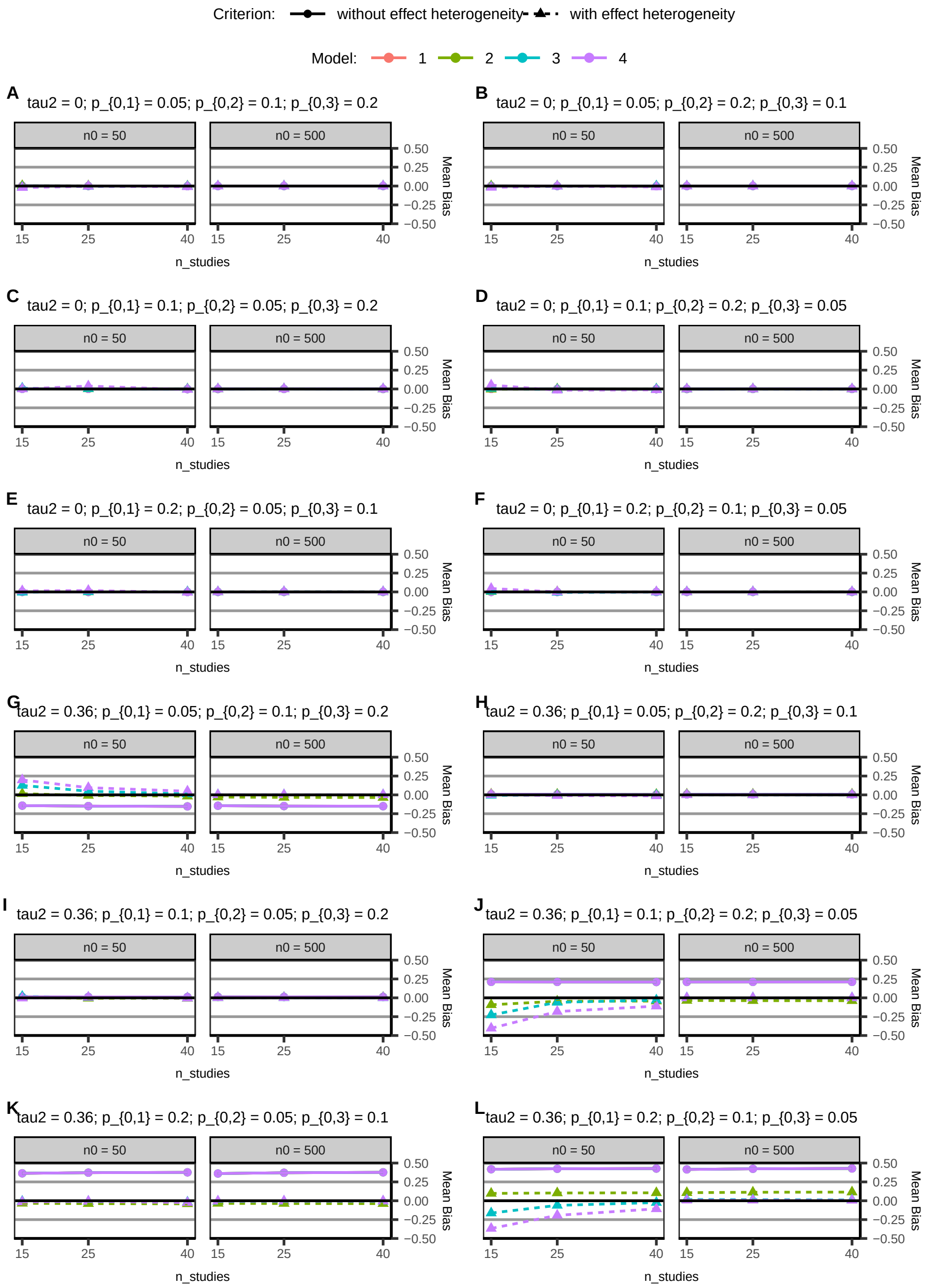

FIGURE S9.
Mean bias of $\hat{\hat{\beta}}$ for each log-linear mixture model for conditions with $S = 3$. The number of components of the respective model is colour-coded. Models with identical slopes are depicted with solid lines. Models with varying slope are depicted with dashed lines. A-F: Simulation conditions with $\tau^2 = 0$ (homogeneous effects), G-L: Simulation conditions with $\tau^2 = 0.36$ (heterogeneous effects).

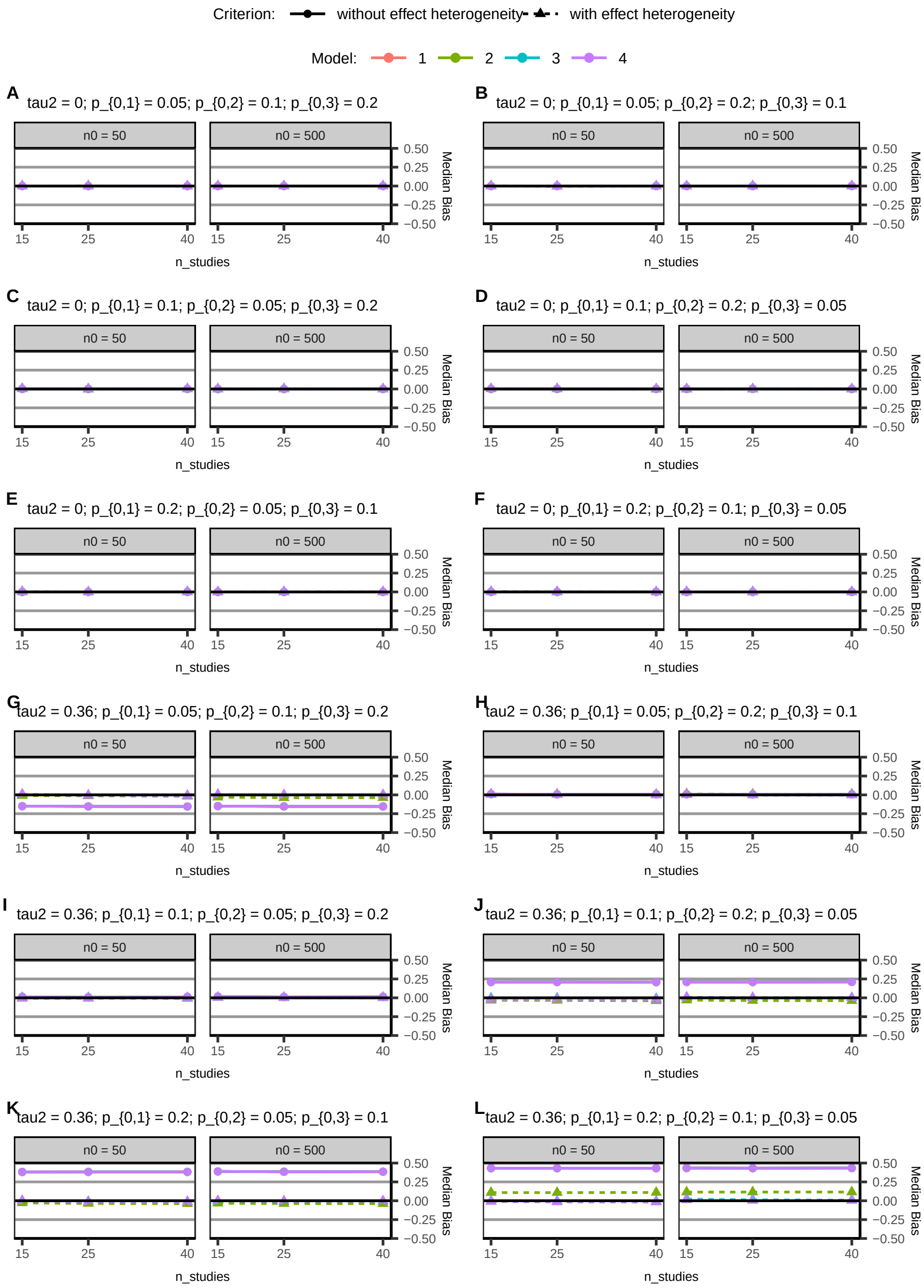

FIGURE S10.

Median bias of $\hat{\hat{\beta}}$ for each log-linear mixture model for conditions with $S = 3$. The number of components of the respective model is colour-coded. Models with identical slopes are depicted with solid lines. Models with varying slope are depicted with dashed lines. A-F: Simulation conditions with $\tau^2 = 0$ (homogeneous effects), G-L: Simulation conditions with $\tau^2 = 0.36$ (heterogeneous effects).

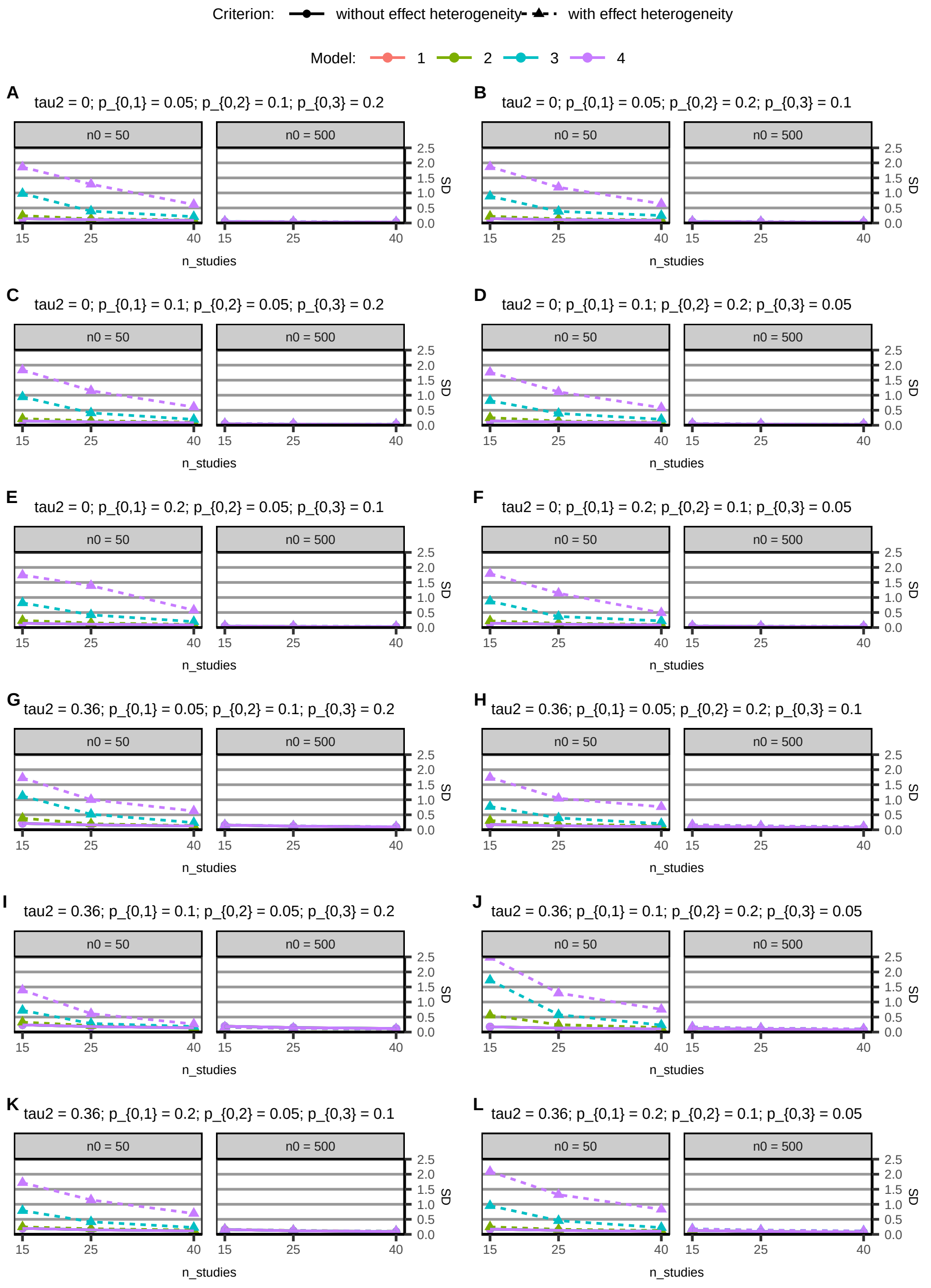

FIGURE S11.
Standard deviation of $\hat{\hat{\beta}}$ for each log-linear mixture model for conditions with $S = 3$. The number of components of the respective model is colour-coded. Models with identical slopes are depicted with solid lines. Models with varying slope are depicted with dashed lines. A-F: Simulation conditions with $\tau^2 = 0$ (homogeneous effects), G-L: Simulation conditions with $\tau^2 = 0.36$ (heterogeneous effects).

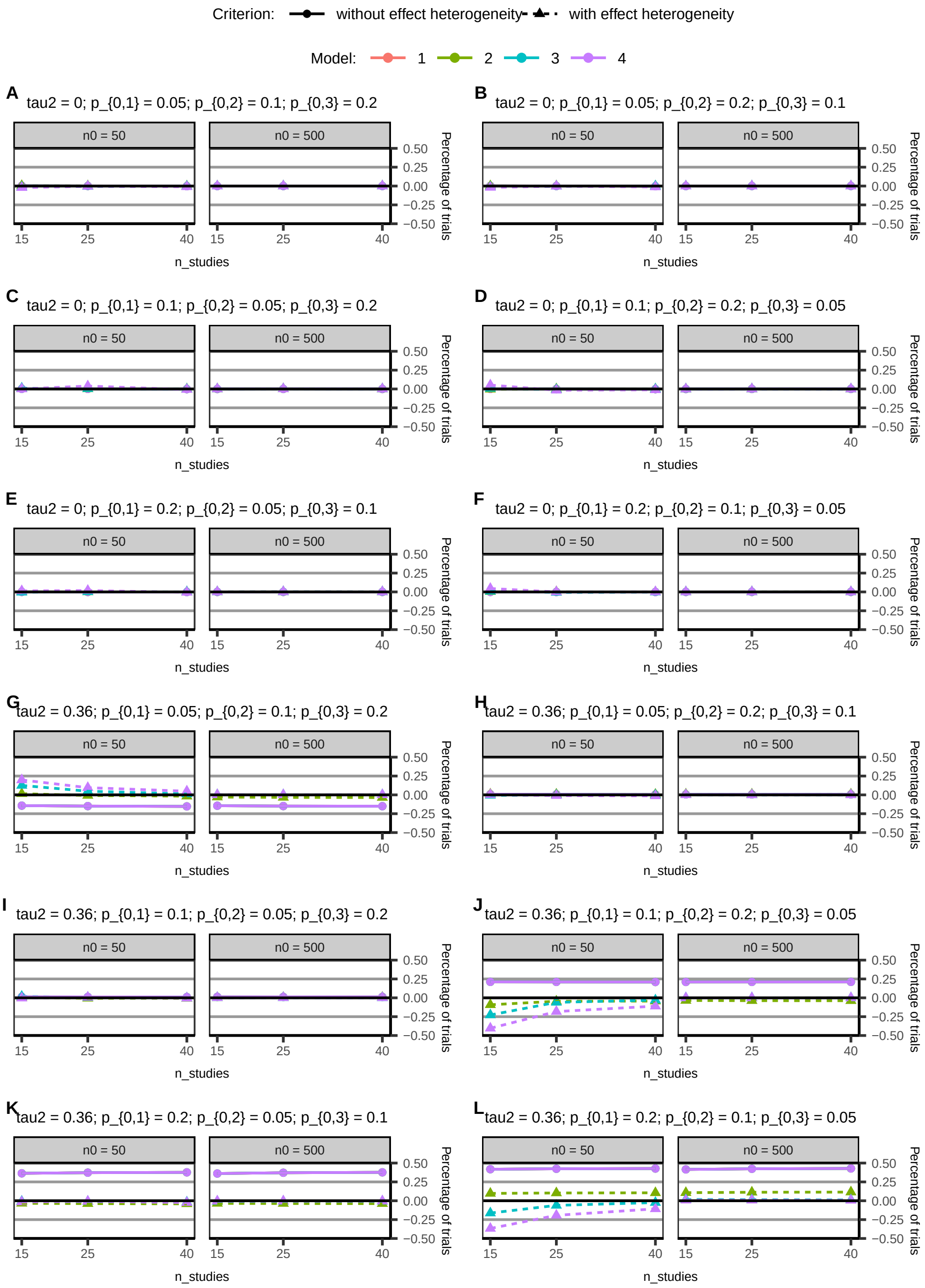

FIGURE S12.
Mean bias of $\hat{\hat{\beta}}$ for each logistic mixture model for conditions with $S = 3$. The number of components of the respective model is colour-coded. Models with identical slopes are depicted with solid lines. Models with varying slope are depicted with dashed lines. A-F: Simulation conditions with $\tau^2 = 0$ (homogeneous effects), G-L: Simulation conditions with $\tau^2 = 0.36$ (heterogeneous effects).

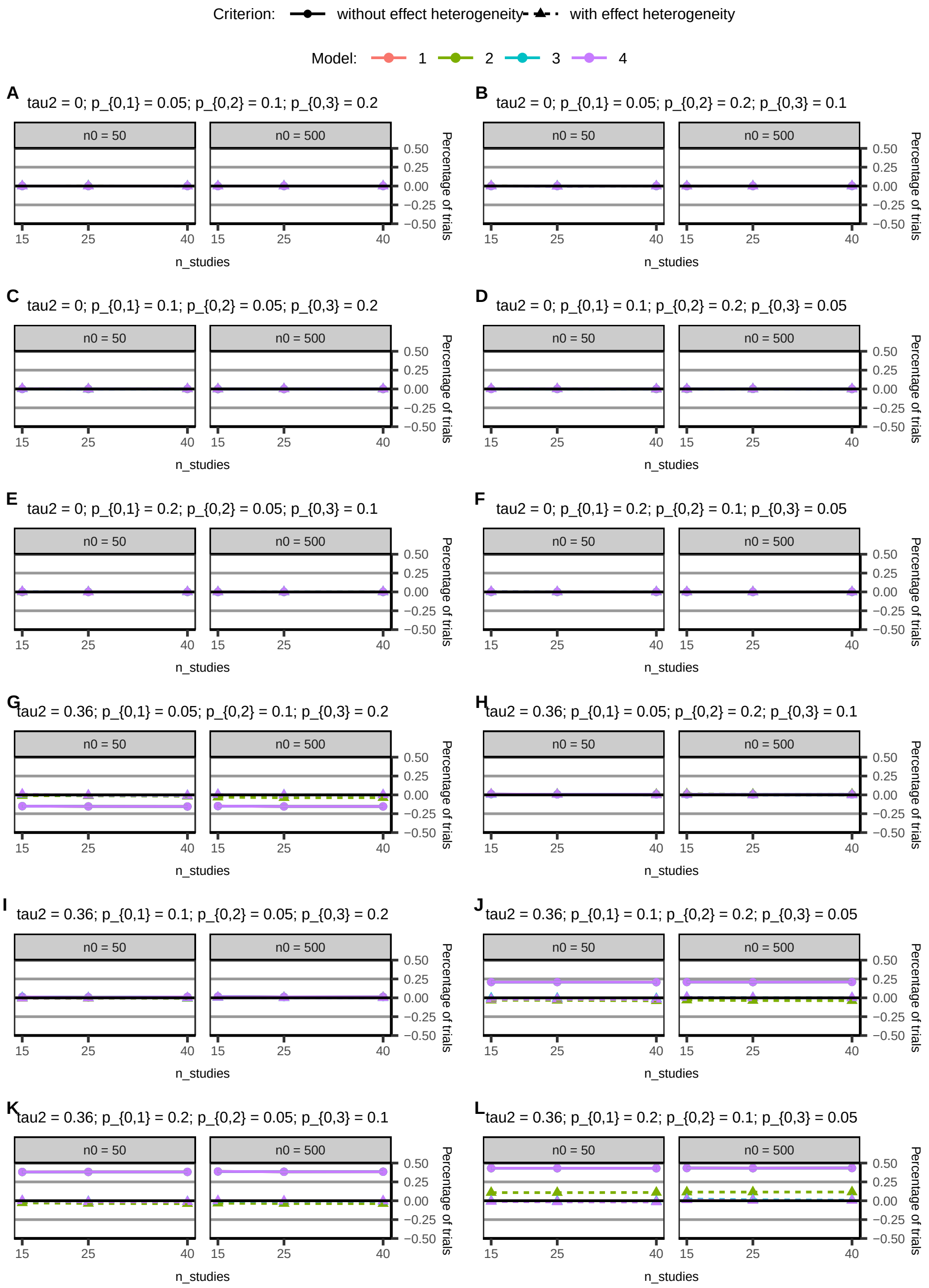

FIGURE S13.
Median bias of $\hat{\hat{\beta}}$ for each logistic mixture model for conditions with $S = 3$. The number of components of the respective model is colour-coded. Models with identical slopes are depicted with solid lines. Models with varying slope are depicted with dashed lines. A-F: Simulation conditions with $\tau^2 = 0$ (homogeneous effects), G-L: Simulation conditions with $\tau^2 = 0.36$ (heterogeneous effects).

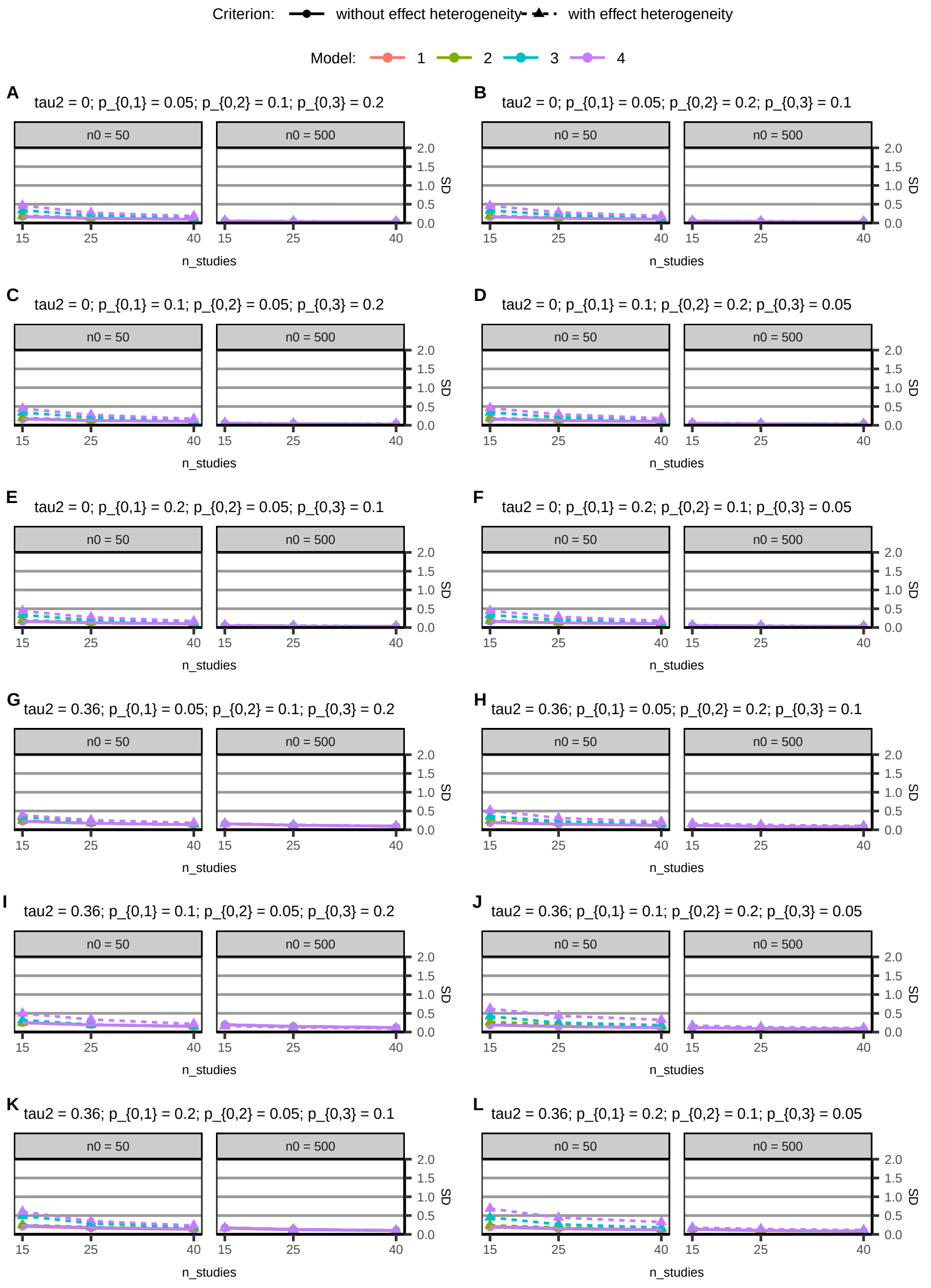

FIGURE S14.

Standard deviation of $\hat{\hat{\beta}}$ for each logistic mixture model for conditions with $S = 3$. The number of components of the respective model is colour-coded. Models with identical slopes are depicted with solid lines. Models with varying slope are depicted with dashed lines. A-F: Simulation conditions with $\tau^2 = 0$ (homogeneous effects), G-L: Simulation conditions with $\tau^2 = 0.36$ (heterogeneous effects).

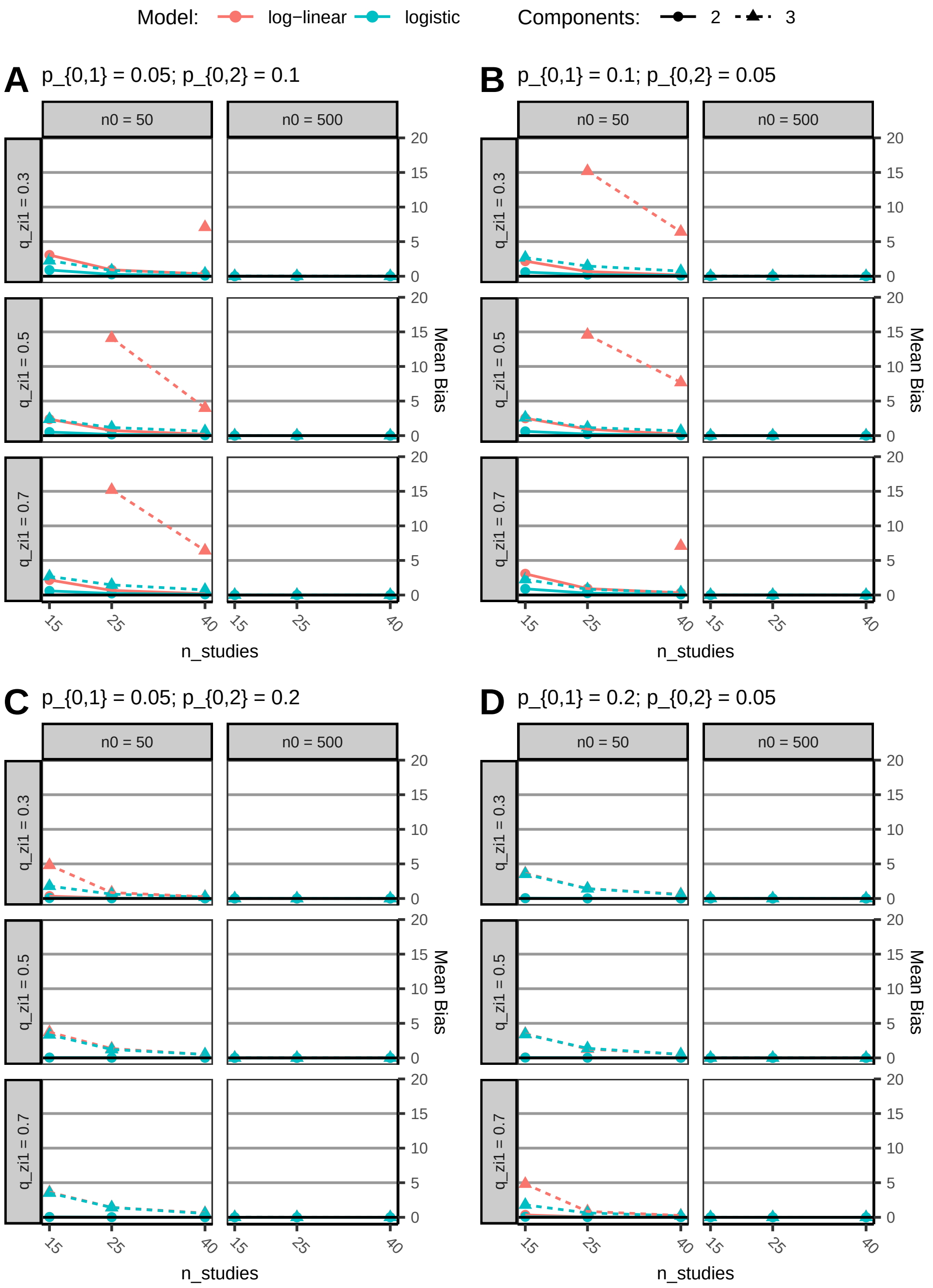

FIGURE S15.

Mean Bias of $\hat{\tau}^2$ for conditions with $S = 2$. Results for the log-linear model are depicted in red, results for the logistic model are depicted in blue. Models with 2 and 3 components are depicted with solid and dashed lines, respectively. A-D: Simulation conditions with $\tau^2 = 0$ (homogeneous effects), E-H: Simulation conditions with $\tau^2 = 0.36$ (heterogeneous effects). Missing values were outside the range of the y-axis.

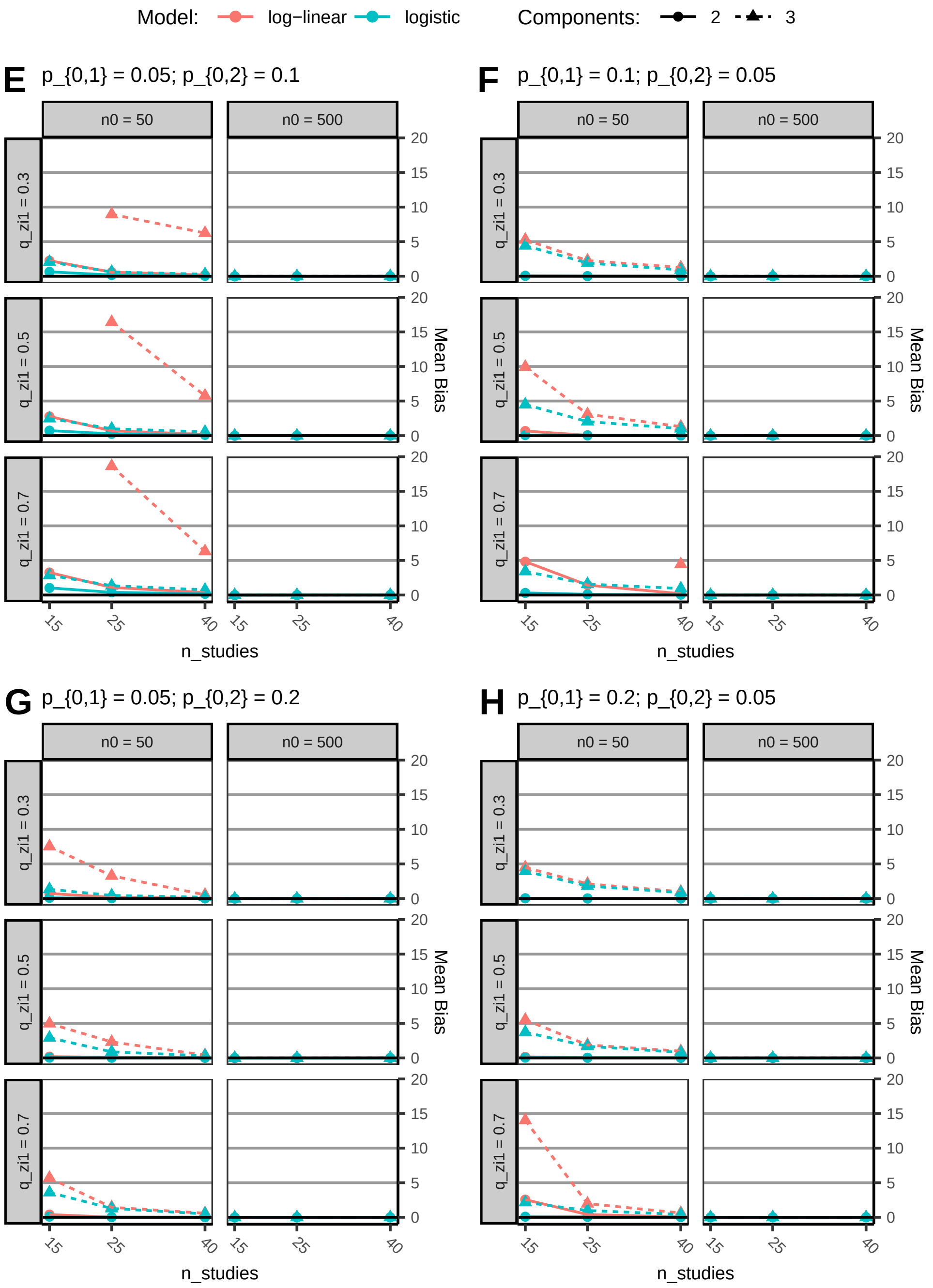

FIGURE S15.
(continued) Mean Bias of $\hat{\tau}^2$ for conditions with $S = 2$. Results for the log-linear model are depicted in red, results for the logistic model are depicted in blue. Models with 2 and 3 components are depicted with solid and dashed lines, respectively. A-D: Simulation conditions with $\tau^2 = 0$ (homogeneous effects), E-H: Simulation conditions with $\tau^2 = 0.36$ (heterogeneous effects). Missing values were outside the range of the y-axis.

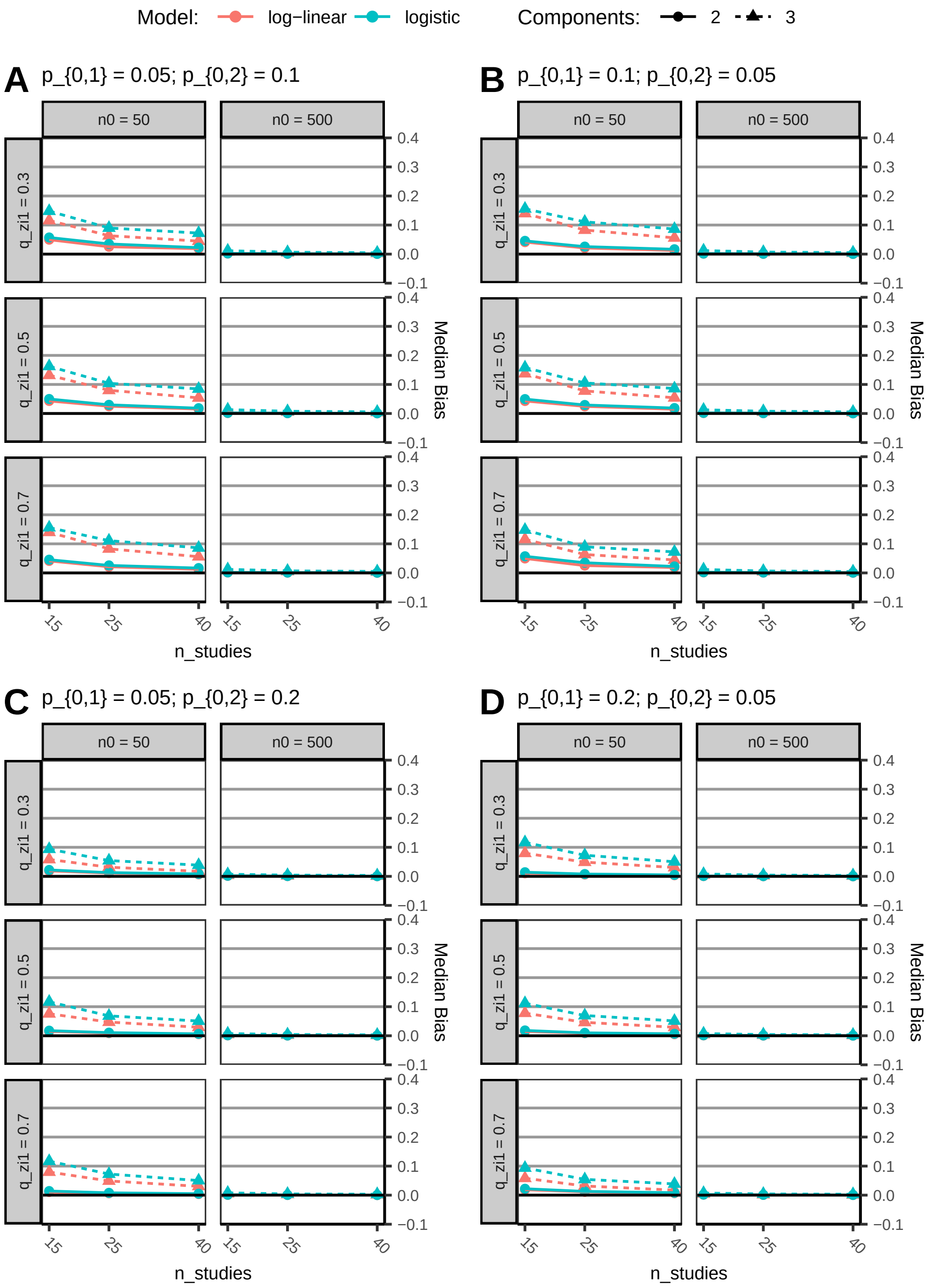

FIGURE S16.
Median Bias of $\hat{\tau}^2$ for conditions with $S = 2$. Results for the log-linear model are depicted in red, results for the logistic model are depicted in blue. Models with 2 and 3 components are depicted with solid and dashed lines, respectively. A-D: Simulation conditions with $\tau^2 = 0$ (homogeneous effects), E-H: Simulation conditions with $\tau^2 = 0.36$ (heterogeneous effects).

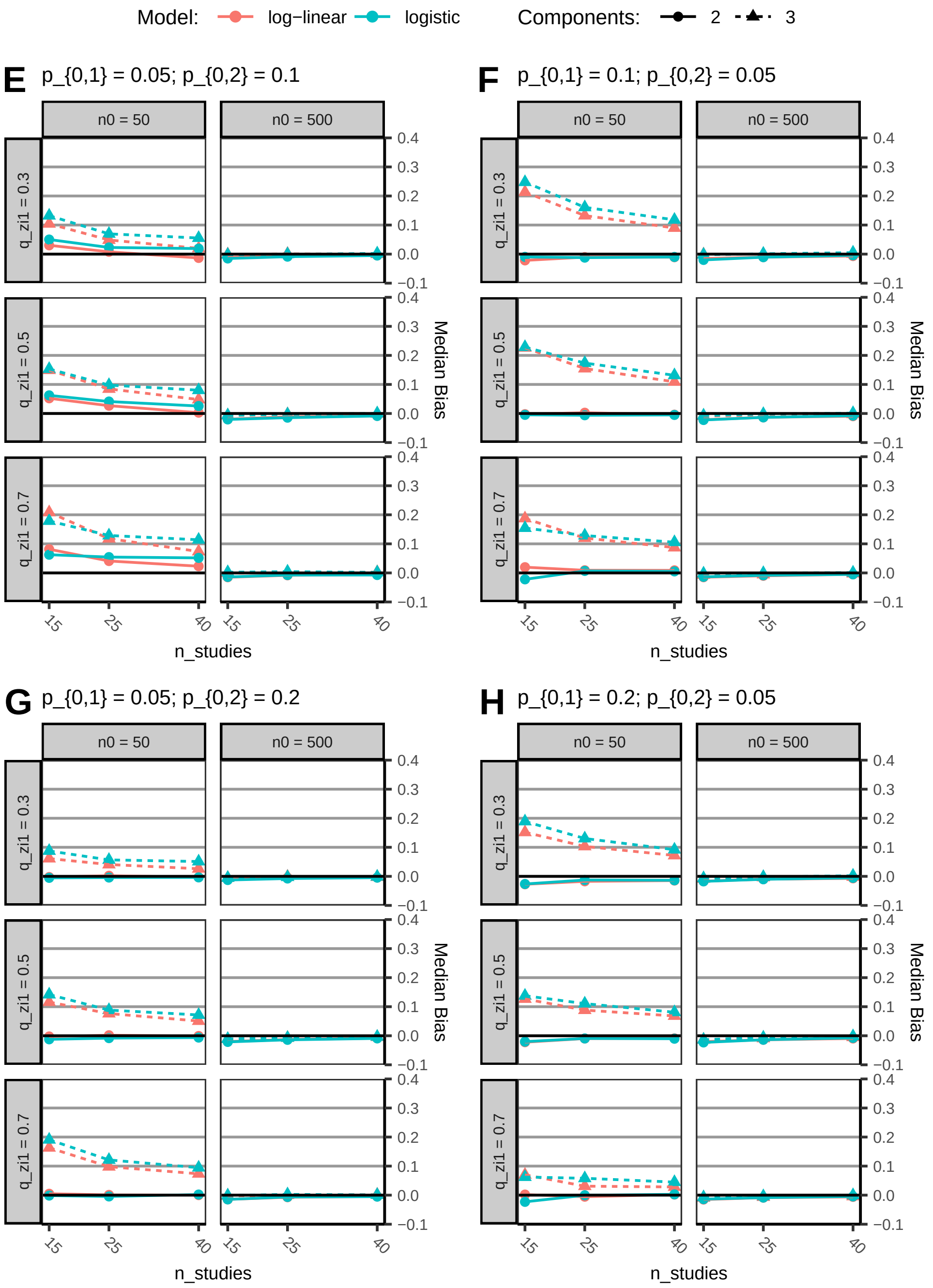

Figure S16.
(continued) Median Bias of $\hat{\tau}^2$ for conditions with $S = 2$. Results for the log-linear model are depicted in red, results for the logistic model are depicted in blue. Models with 2 and 3 components are depicted with solid and dashed lines, respectively. A-D: Simulation conditions with $\tau^2 = 0$ (homogeneous effects), E-H: Simulation conditions with $\tau^2 = 0.36$ (heterogeneous effects).

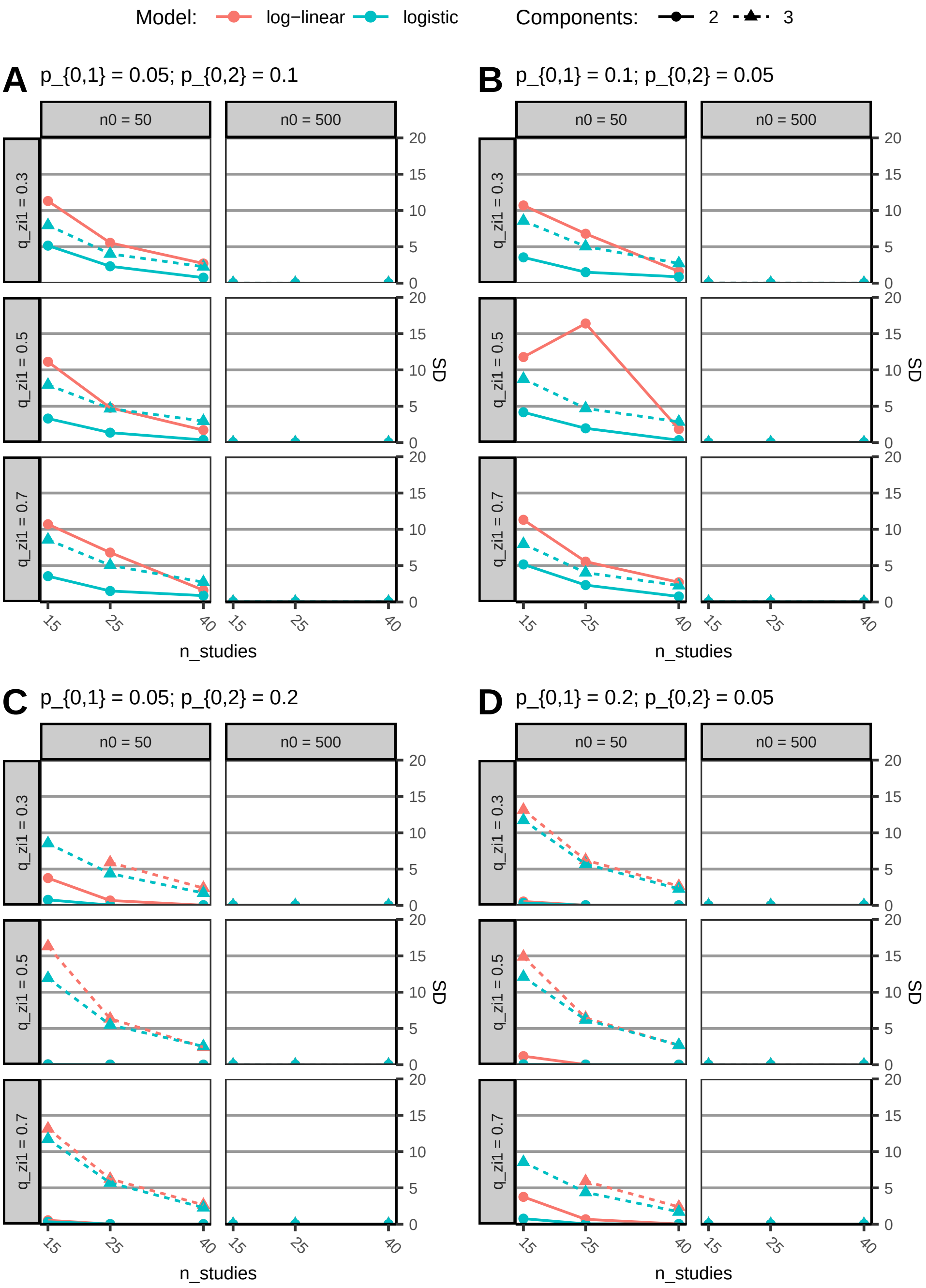

Figure S17.
Standard deviation of $\hat{\tau}^2$ for conditions with $S = 2$. Results for the log-linear model are depicted in red, results for the logistic model are depicted in blue. Models with 2 and 3 components are depicted with solid and dashed lines, respectively. A-D: Simulation conditions with $\tau^2 = 0$ (homogeneous effects), E-H: Simulation conditions with $\tau^2 = 0.36$ (heterogeneous effects). Missing values were outside the range of the y-axis.

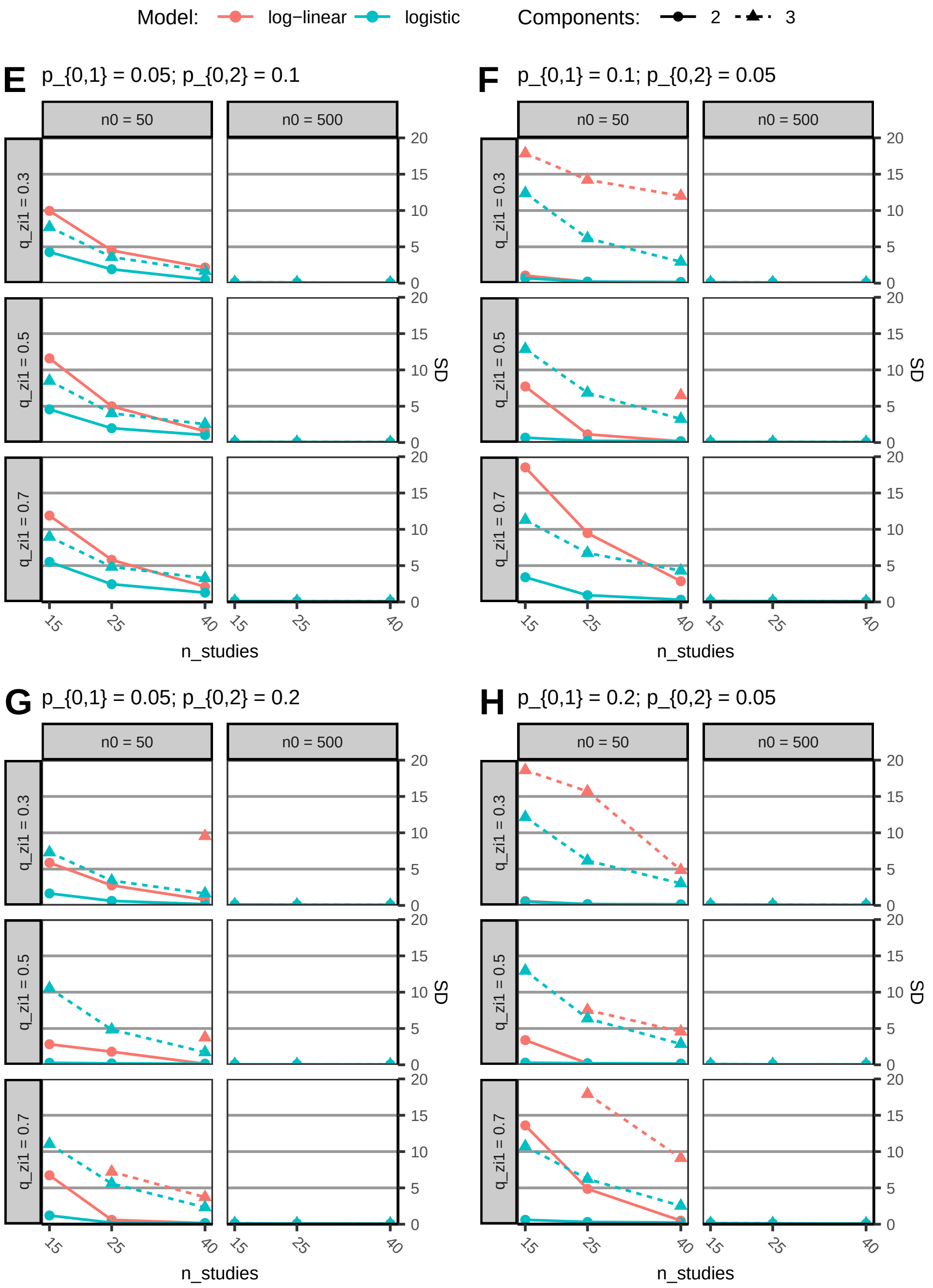

Figure S17.
(continued) Standard deviation of $\hat{\tau}^2$ for conditions with $S = 2$. Results for the log-linear model are depicted in red, results for the logistic model are depicted in blue. Models with 2 and 3 components are depicted with solid and dashed lines, respectively. A-D: Simulation conditions with $\tau^2 = 0$ (homogeneous effects), E-H: Simulation conditions with $\tau^2 = 0.36$ (heterogeneous effects). Missing values were outside the range of the y-axis.

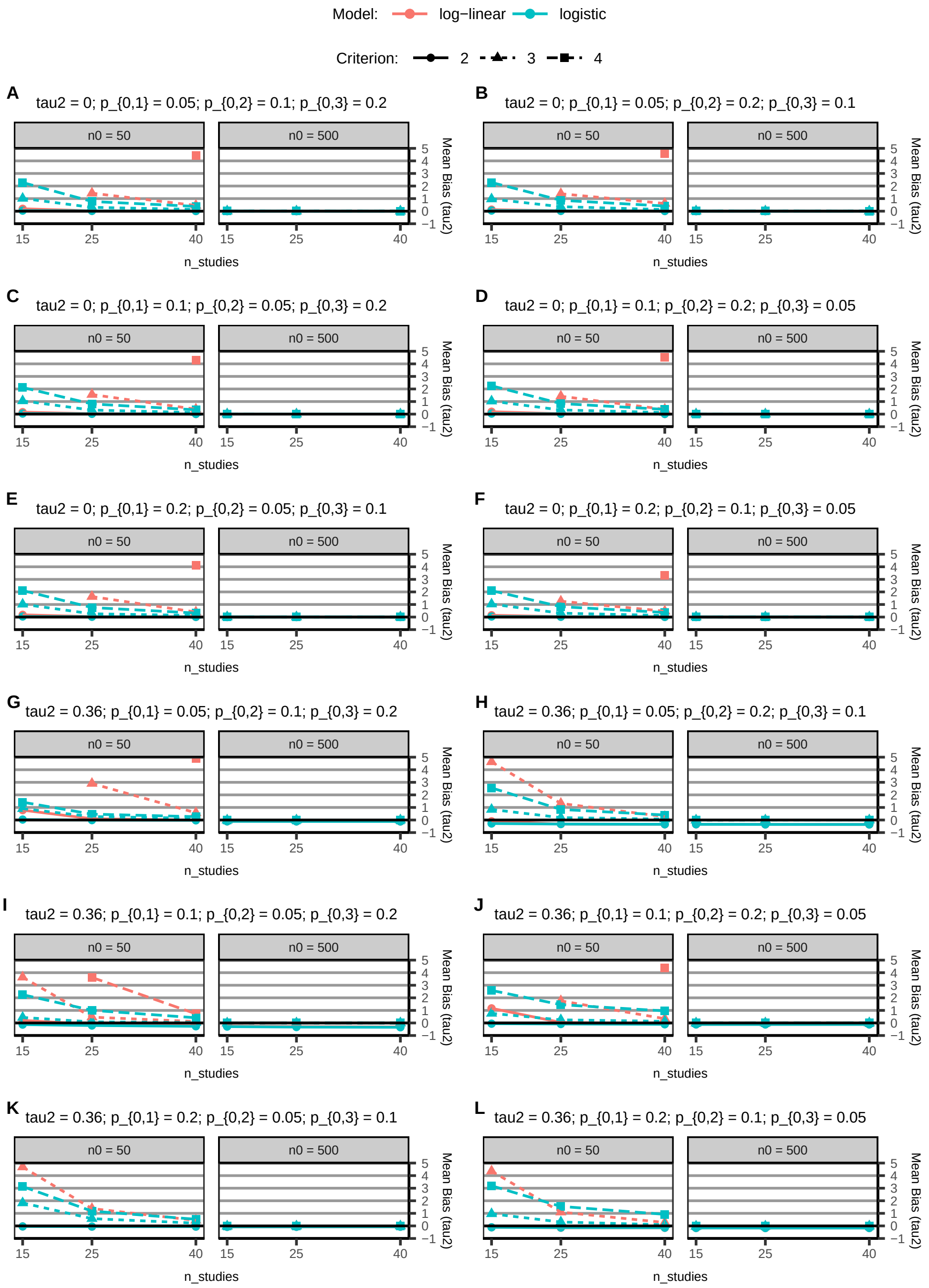

FIGURE S18.

Mean Bias of $\hat{\tau}^2$ for conditions with $S = 3$. Results for the log-linear model are depicted in red, results for the logistic model are depicted in blue. Models with 2, 3 and 4 components are depicted with solid and dashed and long-dashed lines, respectively. A-F: Simulation conditions with $\tau^2 = 0$ (homogeneous effects), G-L: Simulation conditions with $\tau^2 = 0.36$ (heterogeneous effects). Missing values were outside the range of the y-axis.

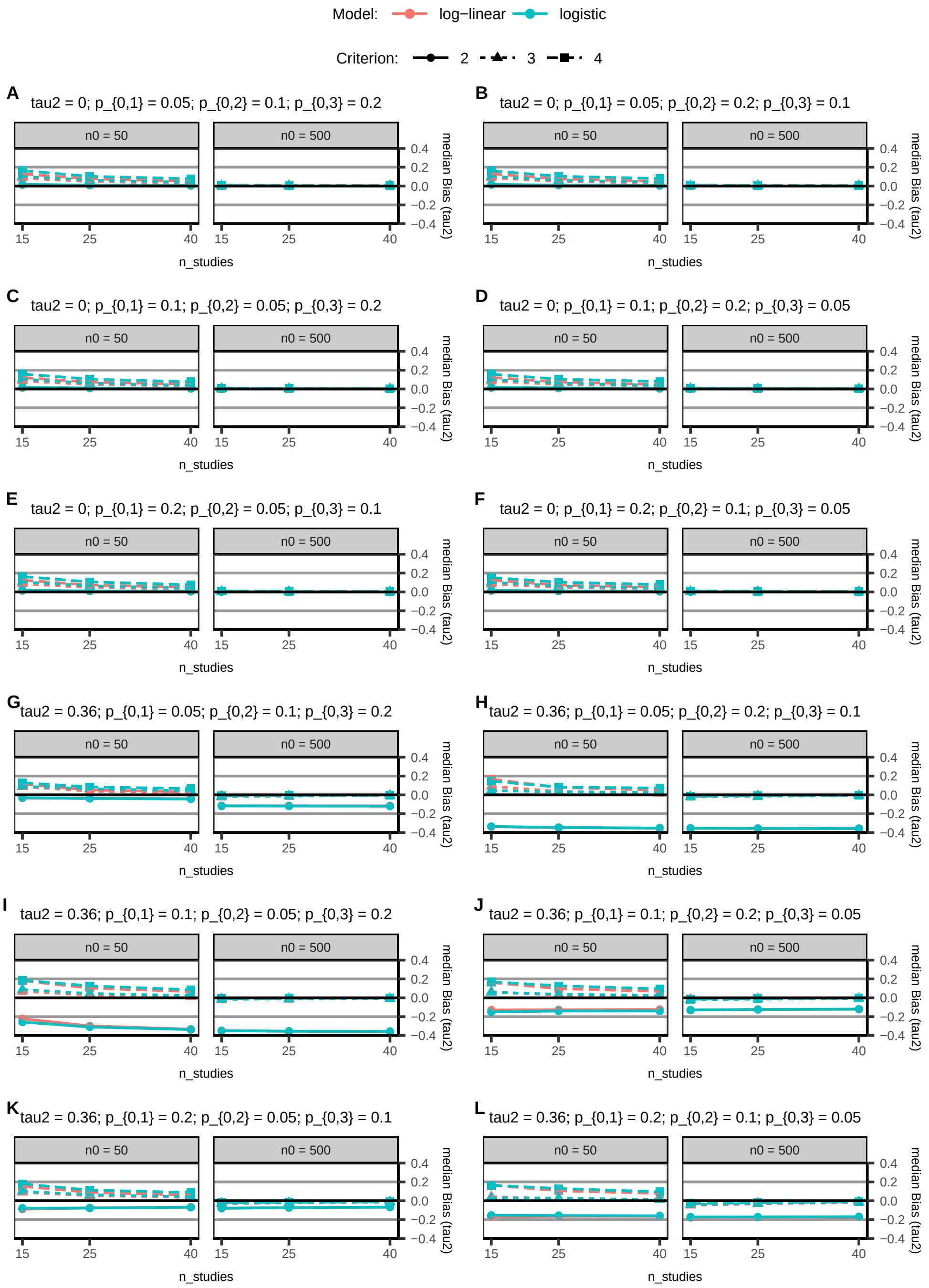

FIGURE S19.

Median Bias of $\hat{\tau}^2$ for conditions with $S = 3$. Results for the log-linear model are depicted in red, results for the logistic model are depicted in blue. Models with 2, 3 and 4 components are depicted with solid and dashed and long-dashed lines, respectively. A-F: Simulation conditions with $\tau^2 = 0$ (homogeneous effects), G-L: Simulation conditions with $\tau^2 = 0.36$ (heterogeneous effects).

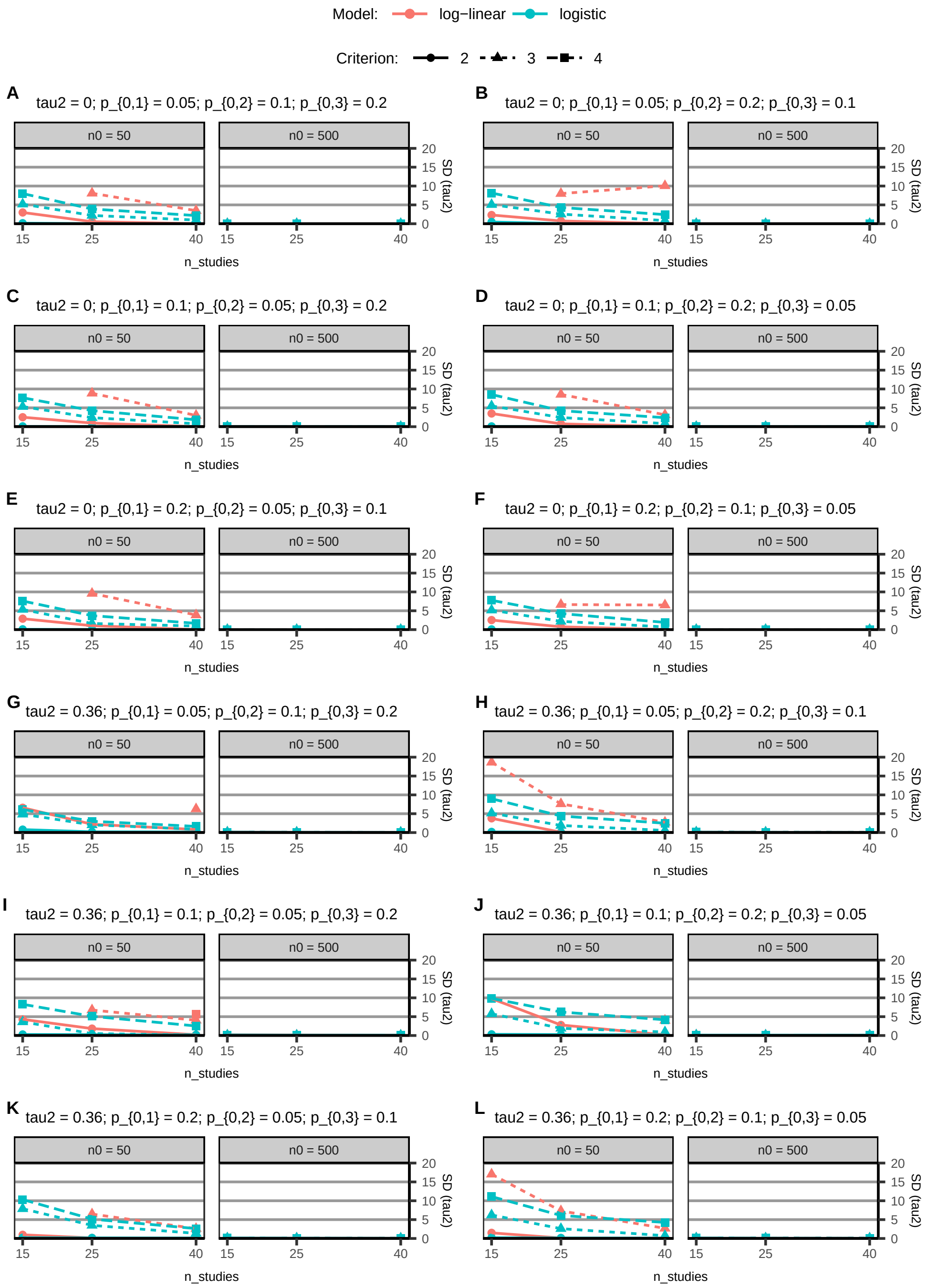

FIGURE S20.

Standard deviation of $\hat{\tau}^2$ for conditions with $S = 3$. Results for the log-linear model are depicted in red, results for the logistic model are depicted in blue. Models with 2, 3 and 4 components are depicted with solid and dashed and long-dashed lines, respectively. A-F: Simulation conditions with $\tau^2 = 0$ (homogeneous effects), G-L: Simulation conditions with $\tau^2 = 0.36$ (heterogeneous effects). Missing values were outside the range of the y-axis.

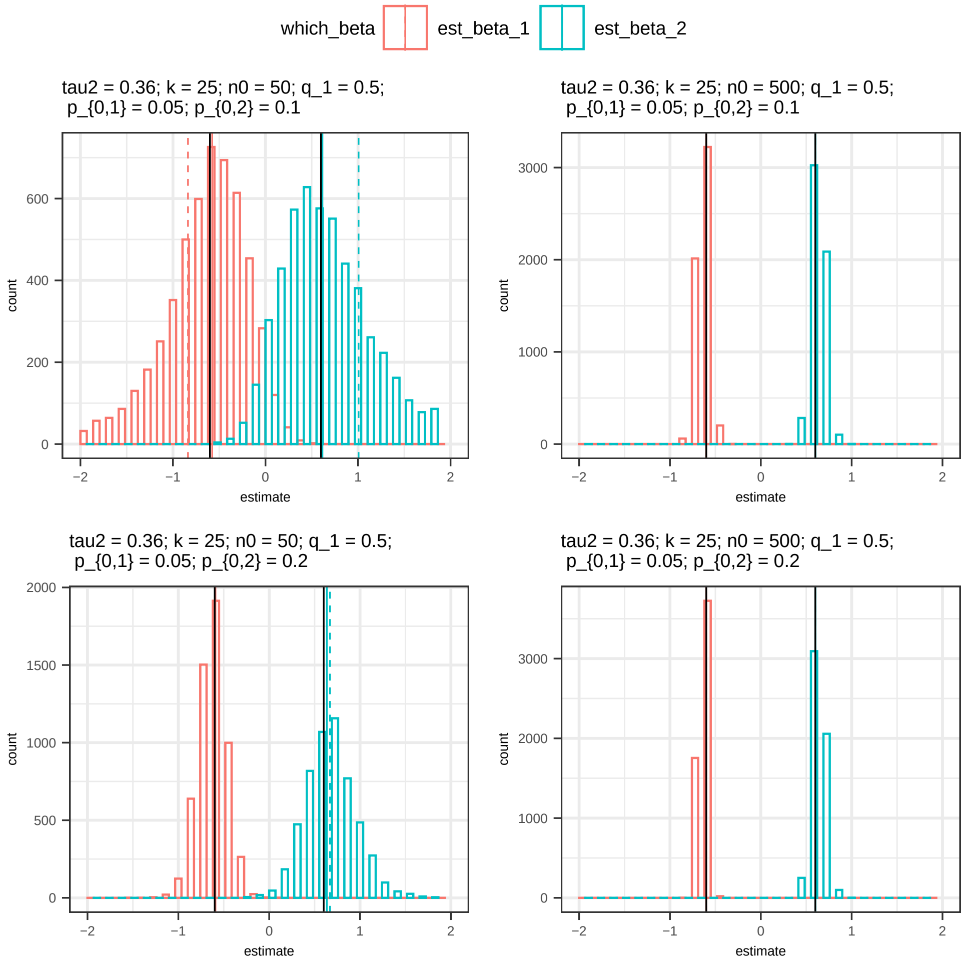

Figure S21.

Histograms of $\hat{\beta}_1$ and $\hat{\beta}_2$ estimated by a log-linear model with two components and with effect heterogeneity for selected simulation conditions of the simulation with $S = 2$. Means (dashed line) and medians (solid line) of $\hat{\beta}_1$, $\hat{\beta}_2$, $\hat{\beta}_3$ are shown in the same colour as the respective histogram.

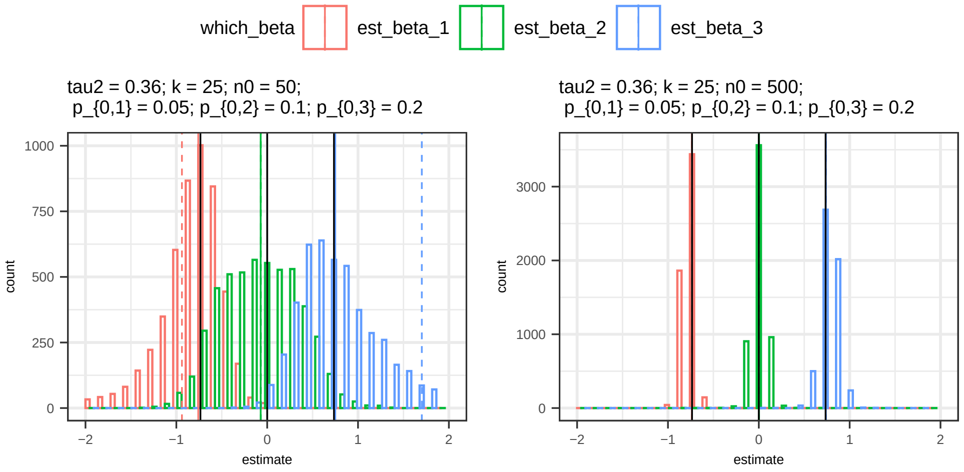

FIGURE S22.
Histograms of $\hat{\beta}_1$, $\hat{\beta}_2$ and $\hat{\beta}_3$ estimated by a log-linear model with three components and with effect heterogeneity for selected simulation conditions of the simulation with $S = 3$. Solid black lines indicate the true values of β_1, β_2 and β_3. Means (dashed line) and medians (solid line) of $\hat{\beta}_1$, $\hat{\beta}_2$, $\hat{\beta}_3$ are shown in the same colour as the respective histogram.
